# Host nucleolin-targeting unique peptide drugs as potent broad-spectrum anti-influenza therapies

**DOI:** 10.1093/pnasnexus/pgag269

**Published:** 2026-09-01

**Authors:** Thu Ha Nguyen, Muhammad Sharif, Jae-Hyun Kim, Yeong-Bin Baek, Mahmoud Soliman, Hyung-Jun Kwon, Seong-Hun Jeong, Ragab Farouk, Shaimaa Aboelmagd, Jae-Ha Ryu, Gyeong Min Kim, Woonsung Na, Don-Kyu Kim, Jeong-Sun Kim, Dong Ju Lee, Jae Il Kim, Kyoung-Oh Cho

**Affiliations:** Host-Directed Antiviral Research Center, Chonnam National University, Gwangju 61186, South Korea; Host-Directed Antiviral Research Center, Chonnam National University, Gwangju 61186, South Korea; School of Life Sciences, Gwangju Institute of Science and Technology, Gwangju 61005, South Korea; Host-Directed Antiviral Research Center, Chonnam National University, Gwangju 61186, South Korea; Department of Pathology and Clinical Pathology, Faculty of Veterinary Medicine, Assiut University, Assiut 71526, Egypt; Host-Directed Antiviral Research Center, Chonnam National University, Gwangju 61186, South Korea; Functional Biomaterials Research Center, Korea Research Institute of Bioscience and Biotechnology, Jeongeup-si, Jeollabuk-do 56212, South Korea; Host-Directed Antiviral Research Center, Chonnam National University, Gwangju 61186, South Korea; Functional Biomaterials Research Center, Korea Research Institute of Bioscience and Biotechnology, Jeongeup-si, Jeollabuk-do 56212, South Korea; Host-Directed Antiviral Research Center, Chonnam National University, Gwangju 61186, South Korea; Host-Directed Antiviral Research Center, Chonnam National University, Gwangju 61186, South Korea; Pilot Plant, HLB PEP, Gwangju Technopark, Gwangju 61008, South Korea; Pilot Plant, HLB PEP, Gwangju Technopark, Gwangju 61008, South Korea; Department of Oral Microbiology and Immunology, and Dental Research Institute, School of Dentistry, Seoul National University, Seoul 03080, South Korea; Host-Directed Antiviral Research Center, Chonnam National University, Gwangju 61186, South Korea; Department of Integrative Food, Bioscience, and Biotechnology, Chonnam National University, Gwangju 61186, South Korea; Host-Directed Antiviral Research Center, Chonnam National University, Gwangju 61186, South Korea; Department of Chemistry, Chonnam National University, Gwangju 61186, South Korea; Host-Directed Antiviral Research Center, Chonnam National University, Gwangju 61186, South Korea; School of Life Sciences, Gwangju Institute of Science and Technology, Gwangju 61005, South Korea; Pilot Plant, HLB PEP, Gwangju Technopark, Gwangju 61008, South Korea; Host-Directed Antiviral Research Center, Chonnam National University, Gwangju 61186, South Korea

**Keywords:** influenza, nucleolin, AGM-380d and AGM-380t, blockage of nucleoprotein trafficking, host-directed antivirals

## Abstract

The persistent threat of influenza pandemics and seasonal outbreaks, coupled with rising resistance to existing antiviral drugs targeting influenza viruses, necessitates the development of new therapeutics. This study investigated the antiviral potential of cell-penetrating peptide–conjugated host nucleolin (NCL)-binding peptide drugs, AGM-380d (dimeric) and AGM-380t (tetrameric), as innovative, broad-spectrum host-directed anti-influenza therapies. Infection with both influenza A and B viruses (IAVs and IBVs) significantly increased NCL expression and colocalization with viral nucleoprotein (NP), a core component of viral ribonucleoprotein complexes, in both in vitro and in vivo settings. The peptide drugs rapidly localized to the nucleolus and specifically bound to NCL. This targeted binding successfully disrupted the interaction between NCL and the NP of IAV and IBV, effectively blocking nuclear NP trafficking and inhibiting the replication of both pandemic and seasonal strains. Importantly, AGM-380d and AGM-380t protected mice from lethal IAV challenge by markedly reducing lung viral replication and pathology. The combination of AGM-380t with oseltamivir resulted in 100% protection from mortality following the deadly IAV challenge. In conclusion, AGM-380d and AGM-380t are highly promising as novel, broad-spectrum host-directed antivirals for the management of seasonal and pandemic influenza.

Significance statement The rapid evolution of influenza viruses and the frequent emergence of drug-resistant strains highlight a major global health challenge. Current antivirals, such as neuraminidase inhibitors, face diminishing efficacy due to mutation-driven resistance, necessitating novel treatment strategies. Host-directed therapies such as AGM-380d and AGM-380t, which target host nucleolin to disrupt viral replication, represent novel approaches with broad-spectrum efficacy against both seasonal and pandemic influenza strains. These therapies not only inhibit viral replication but also enhance survival in lethal infection models, especially when combined with virus-targeting antiviral drugs like oseltamivir. Our research advances antiviral drug development by offering a compelling strategy to overcome drug resistance and improve influenza management worldwide, with significant implications for pandemic preparedness and public health outcomes.

## Introduction

Seasonal influenza, caused by infection with influenza A and B viruses (IAVs and IBVs), is responsible for an estimated 290,000 to 650,000 annual global deaths from respiratory illness (1). Moreover, the introduction of novel IAV strains to largely nonimmune human populations has historically triggered major influenza pandemics, leading to severe worldwide mortality (2). Influenza viruses mutate rapidly during genome replication because their RNA-dependent RNA polymerase lacks proofreading exonuclease activity (3–5). This rapid mutation drives the emergence of new strains, which can lead to drug resistance, including to the M2 channel blocker amantadine and the neuraminidase (NA) inhibitor oseltamivir (6–8). The current concern regarding highly pathogenic avian influenza H5N1 viruses in dairy cattle and farmed mink, and their potential spread to other mammalian species, including humans, underscores the risk of a new pandemic strain emerging (9–12). These evolutionary pressures underscore the urgent need for novel, broad-spectrum antiviral drugs—particularly host-directed antivirals—that can effectively treat both existing mutant and emerging pandemic strains (6–8, 13).

Nucleolin (NCL)—a multifunctional phosphoprotein—is predominantly not only found in the nucleolus but also present in the nucleoplasm, cytoplasm, and cell membrane (14). The tripartite structure of NCL enables interactions with diverse proteins and RNA sequences; its N-terminal domain, rich in acidic stretches, regulates rDNA transcription via several protein–protein interactions. The central globular domain contains four RNA-binding domains, which are crucial for pre-RNA processing. Finally, the C-terminal domain, which has arginine–glycine–glycine repeats, interacts with ribosomal proteins (14, 15). These interactions are linked to various cellular processes, such as ribosome biogenesis, DNA and RNA metabolism, chromatin organization and stability, cytokinesis, angiogenesis, cell proliferation, regulation of apoptosis, stress response, and microRNA processing (14). NCL plays a significant role in several pathological processes (14). Many viruses hijack NCL to complete their life cycle, utilizing it as a viral receptor and entry enhancer (14, 16–20), viral genome replication (21, 22), virus morphogenesis (23), trafficking and egress (24, 25), virus budding (26), and apoptosis (27–31). NCL is predominantly known to not only facilitate viral proliferation but reports also indicate inhibitory effects on viral replication (32). In addition, opposite roles of NCL—either beneficial or detrimental to virus growth—have been documented even within the same virus (25, 32). Nevertheless, targeting NCL could be a potential avenue for developing antiviral drugs (14).

We previously developed the NCL-binding peptide AGM-330 for cancer diagnosis and therapy, which targets cell-surface NCL by binding to its N-terminal region (1–322), with a *K_d_* of 57.7 ± 12 nM (33). To enable intracellular targeting for antiviral application, this study synthesized AGM-380d and AGM-380t by attaching a cell-penetrating peptide (CPP) to the AGM-330 scaffold. This modification allowed them to rapidly penetrate the plasma and nuclear membranes and bind NCL within the nucleolus, significantly improving upon the prototype AGM-330. Given the high mortality of seasonal influenza and the threat of future pandemics, this study aimed to develop and validate the hypothesis that AGM-380d and AGM-380t could inhibit IAV and IBV by targeting NCL. Notably, both AGM-380d and AGM-380t impeded the nuclear-to-cytoplasmic transport of IAV and IBV nucleoproteins (NPs), thereby blocking IAV and IBV replication. In a lethal mouse model, AGM-380d and AGM-380t protected mice from fatal IAV infection by reducing pulmonary viral replication. Moreover, combining AGM-380t with the IAV NA inhibitor oseltamivir provided complete protection against lethal IAV infection in mice. These findings suggest that AGM-380d and AGM-380t show promise as novel therapeutics for IAV and IBV infections.

## Results

### NCL dynamics in response to infection with IAV and IBV

**Figure 1:**
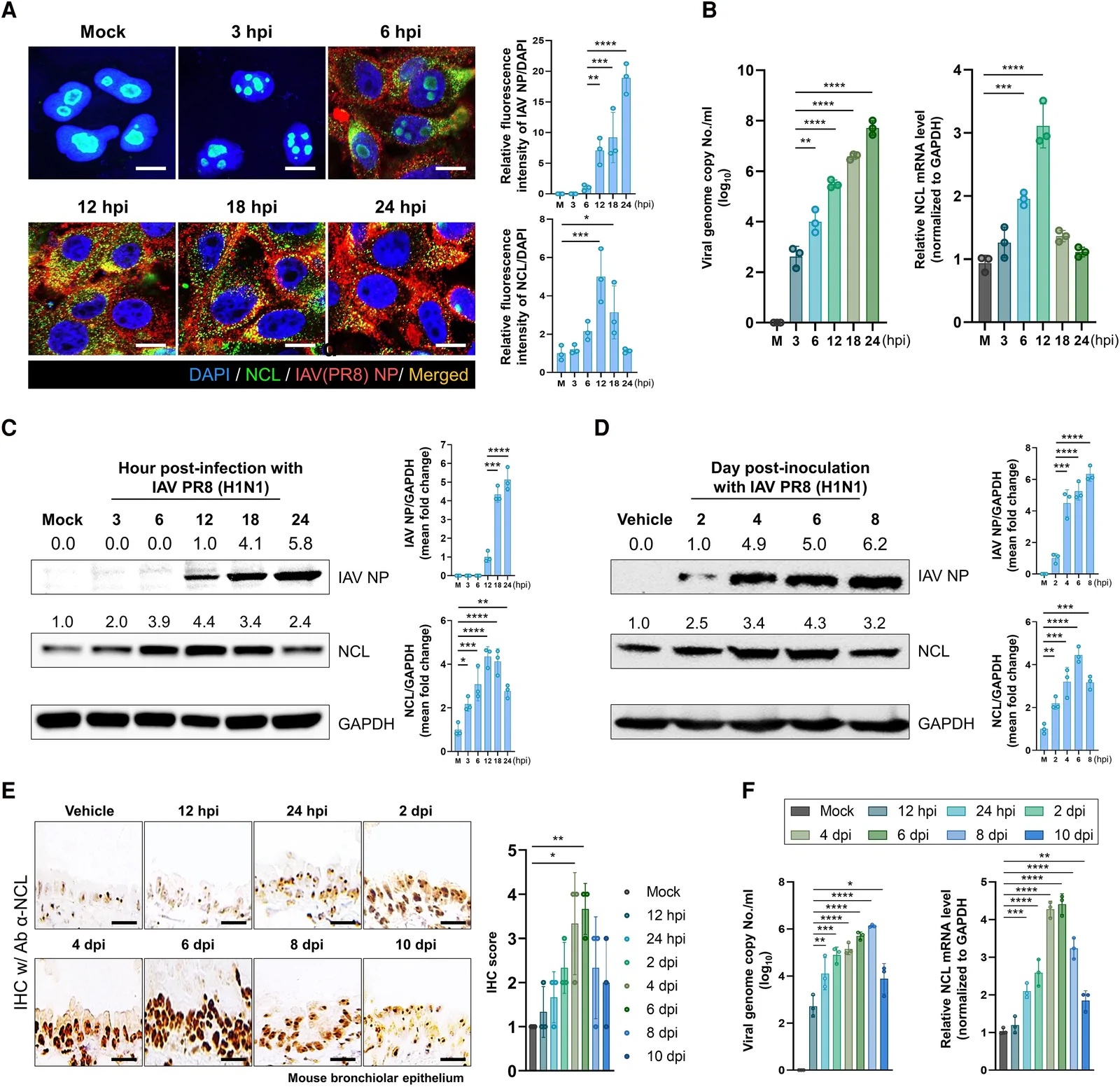
Dynamics of NCL expression during IAV infection, both in vitro and in vivo. A) Representative confocal image (left) and quantification (right) of sequential changes in NCL colocalized with IAV NP in human adenocarcinoma A549 cells infected with the IAV PR8 strain (MOI = 1 FFU) at different time points. The results in each graph are expressed as relative fluorescence intensity, calculated as the mean pixel intensity of positively stained areas in virus-infected groups normalized to the mock-treated, mock-infected control. Bar = 5 µm. B) Graphical representation showing changes in viral genome copy number (left) and NCL mRNA levels (right) at various time points after infecting A549 cells with IAV PR8 strain (MOI = 10 FFU). C) Representative western blot images (left) and quantification (right) of NCL and IAV NP levels after infecting A549 cells with IAV PR8 (MOI = 10 FFU); NCL levels increased up to 12 hpi and then declined. IAV NP levels are also measured to correlate with NCL and viral replication. The intensity of each target protein band is normalized to glyceraldehyde-3-phosphate dehydrogenase (GAPDH) as an internal loading control, and the relative values are presented as mean fold changes. D) Western blot images (left) and graphical representation (right) showing the dynamics of IAV NP and NCL levels at different days postinoculation (dpi) in the lungs of mice infected with 10^3^ PFU of the mouse-adapted IAV PR8 strain. The intensity of each target protein band is normalized to GAPDH as an internal loading control, and the relative values are presented as mean fold changes. E) Representative images (left) and quantification (right) of NCL expression levels in the bronchiolar epithelium of mice infected (*n* = 3) with 10^3^ PFU of the mouse-adapted IAV PR8 strain at different days postinoculation (dpi), detected by immunohistochemistry (IHC) using an antibody against NCL with hematoxylin counterstaining. The results in the graph (right) are expressed as IHC scores, calculated as the mean pixel intensity of positively stained areas in virus-infected groups normalized to the mock-treated, mock-infected control. Bar = 50 µm. F) Graphical representation showing changes in viral genome copy (left) and NCL mRNA levels (right) in lung tissues from mice challenged with 10^3^ PFU of IAV PR8 at various time points. RT-qPCR results (B and F) are normalized to GAPDH, and relative changes in the infected groups are compared with those in mock-infected controls. All data in the graphs are expressed as mean ± SD from three independent experiments. *P < 0.05, **P < 0.01, ***P < 0.001, and ****P < 0.0001; one-way ANOVA with Tukey's correction for multiple comparisons.

In A549 cells infected with different IAV and IBV strains at an multiplicity of infection (MOI) of 1 fluorescence focus unit (FFU) per cell, NCL levels increased up to 12 or 18 h postinfection (hpi) and then declined, while viral genome replication and protein synthesis continued to rise until the end of the experiments (Figs. 1A–C and S1C–F). NCLs colocalized with NPs of IAV and IBV at 12 and 18 hpi, respectively (Figs. 1A and S1A, B). We examined NCL protein and mRNA levels induced by viral infection in normal and tumor cell lines, given their higher expression in tumor cells (33). The level of NCL expression triggered by the IAV PR8 strain varied among normal and tumor cells from different species (Fig. S2A–D). However, the dynamics of NCL after viral infection remained consistent across all cell lines (Fig. S2A–E).

Next, we traced the dynamics of NP, which is also a factor of viral ribonucleoprotein (vRNP) complexes (34). NP entered the nucleus by 2 hpi, transiently colocalized with NCL—a nucleolar marker—at 4 hpi in cells infected with MOI 5 FFU/cell but not MOI 1 FFU/cell, and then relocated to the cytoplasm by 6 hpi in cells infected with both MOI 1 and 5 FFU/cell (Figs. 1A and S3A). To quantify the cytoplasmic and nuclear localization of IAV NP, we harvested A549 cells infected at an MOI of 5 FFU/cell at various time points, isolated the cytoplasm and nucleus, and measured NP levels in each compartment by western blot analysis. Nuclear movement of NP was detected at 2 hpi, with its amount gradually increasing until 6 hpi before declining (Fig. S3B). Conversely, cytoplasmic NP appeared from 2 hpi, and its levels steadily increased to 24 hpi (Fig. S3B). In the bronchiolar epithelial cells from IAV PR8 strain-challenged mice, the levels of NCL increased until day 6 postinoculation (dpi) and then declined (Fig. 1E). NCL protein and mRNA levels in lung samples (Fig. 1D–F) showed similar dynamics in vitro (Fig. 1B and C). In parallel, viral antigen levels and genome copy number increased through 8 dpi and then decreased (Figs. 1F and S3C). Our findings indicate that infection with IAV and IBV, both in vitro and in vivo, induces characteristic NCL dynamics that may interact with the NPs of these viruses (25, 32).

### Characterization of AGM-380d and AGM-380t in association with NCL and NP of IAV and IBV

**Figure 2:**
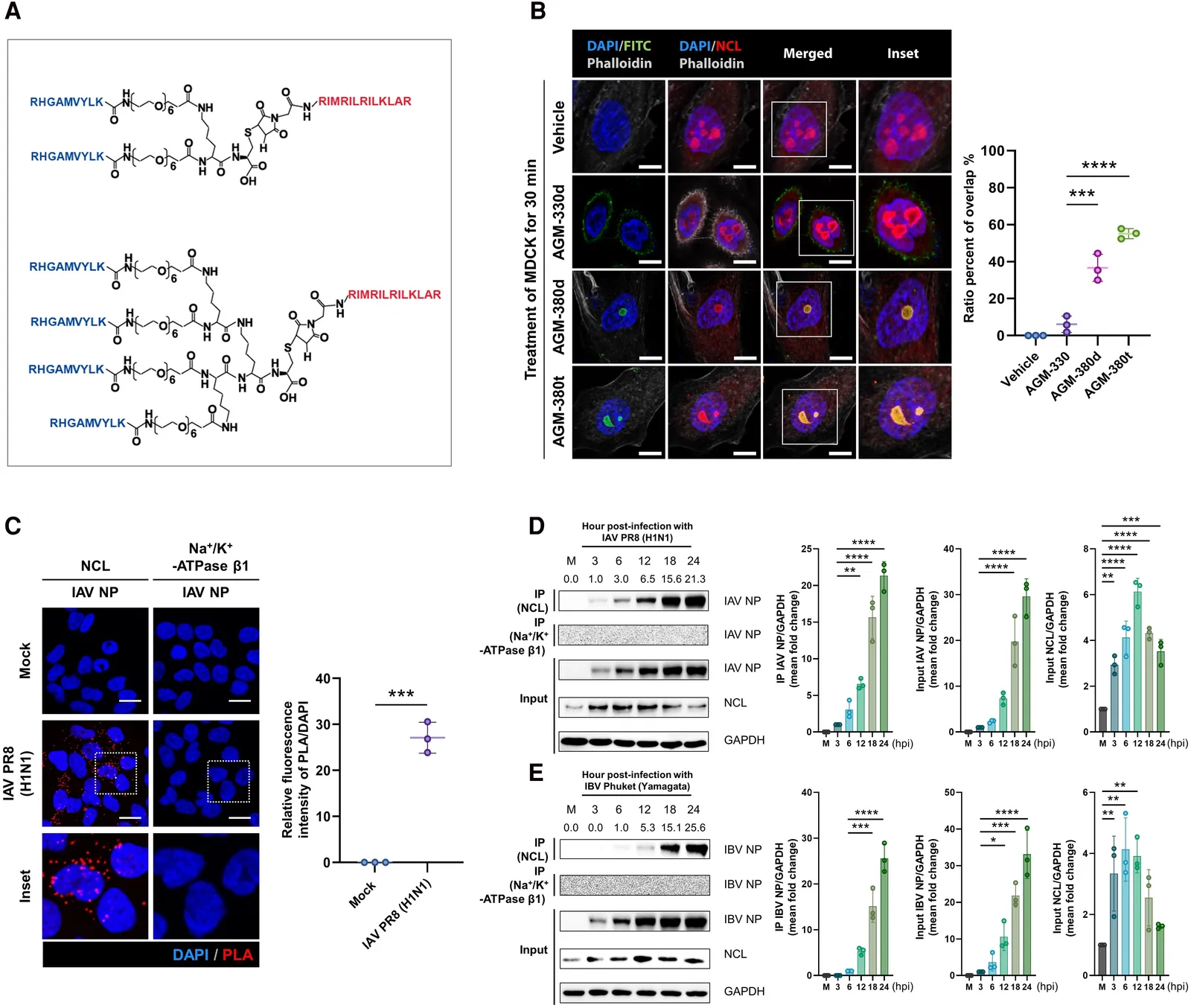
Structure of AGM-380d and AGM-380t and their direct interactions with NCL and NP of IAV and IBV. A) Structure of both AGM-380d and AGM-380t. The blue letters indicate NCL-binding peptides, while the red letters denote CPPs. B) Representative confocal images (left) and quantification (right) of the degree of overlap between intranuclear NCL and FITC-conjugated AGM-330, AGM-380d, or AGM-380t in MDCK cells. Each peptide is used at 2.5 µM and incubated with MDCK cells for 30 min. Actin filaments are stained with Alexa Fluor 488-labeled phalloidin, and nuclear DNA is stained with DAPI. The results in the graph (right) are expressed as the percentage overlap between intranuclear NCL and each FITC-conjugated peptide, calculated as the mean pixel intensity of positively stained areas in virus-infected groups, normalized to the mock-treated, mock-infected control. Scale bar = 5 µm. C) Representative images (left) and quantification (right) of the direct interaction between NCL and the NP of IAV PR8 (H1N1) strain (MOI = 1 FFU) or an irrelevant Na^+^/K^+^-ATPase β1 protein as a negative control, observed 12 h after infection using the Duolink proximity ligation assay. The results in the graph (right) are expressed as relative fluorescent intensity, calculated as the mean pixel intensity of positively stained areas in virus-infected groups normalized to the mock-treated, mock-infected control. Scale bar = 10 µm. D, E) Representative western blot images from immunoprecipitation assays. To determine the interaction between NCL and NP of IAV (D) and IBV (E), A549 cells infected with IAV PR8 (H1N1) or IBV Phuket (Yamagata lineage) strains (MOI = 10 FFU) are harvested at 3, 6, 12, 18, and 18 hpi and immunoprecipitated with an antibody against NCL or with an irrelevant antibody targeting the Na^+^/K^+^-ATPase β1 protein as a negative control. NPs of IAV and IBV, NCL, and GAPDH, either immunoprecipitated or present in each input, are detected using their respective antibodies. The intensity of each target protein band is normalized to GAPDH as an internal loading control, and the relative values are expressed as mean fold changes. All data in the graphs are expressed as mean ± SD from three independent experiments. *P < 0.05, **P < 0.01, ***P < 0.001, and ****P < 0.0001; one-way ANOVA with Tukey's correction for multiple comparisons.

We developed a CPP consisting of 13 amino acids (RIMRILRILKLAR) in linear form, with five cationic residues (Figs. 2A and S4). In our previous study (33), we developed an NCL-targeting peptide, AGM-330, for cancer diagnosis and drug delivery. In this study, we synthesized dimeric AGM-380d and tetrameric AGM-380t by conjugating a CPP to AGM-330d or AGM-330t for potential use in viral disease therapy (Figs. 2A and S4). Remarkably, both dimeric AGM-380d and tetrameric AGM-380t crossed the plasma membrane, reached the nucleus, and mainly colocalized with NCLs in the nucleolus, with some presence in the cytoplasm, indicating their specific NCL binding (Figs. 2B, S5A, and S6A–D). However, fluorescein isothiocyanate (FITC)-conjugated AGM-330d, which lacked CPP conjugation, remained attached to NCL on the cell surface (Figs. 2B and S5A). In addition, silencing of NCL reduced the binding of FITC-conjugated AGM-380d or AGM-380t to NCL (Fig. S6C and D). Given the specific binding affinity of AGM-330 for NCL (33), our data suggest that both AGM-380d and AGM-380t specifically bind to intracellular NCL.

We next examined whether NCL binds to the NPs of IAV and IBV—a key component of vRNP complexes. In addition to observing colocalization of NCL with IAV NPs in confocal images (Figs. 1A and S1A, B), we confirmed its specific binding to NPs of both IAV and IBV through immunoprecipitation and Duolink assays (Figs. 2C, D and S5B–D). In addition, knocking down endogenous NCL inhibited the trafficking of IAV and IBV NPs from the nucleus to the cytoplasm and reduced IAV and IBV genome replication and progeny virus production (Fig. S7). These results suggest that NCL binds to the NP of IAV and IBV, aiding the movement of vRNP complexes from the nucleus to the cytoplasm, where they generate progeny viruses (25).

### Suppression of IAV and IBV replication by AGM-380d and AGM-380t through blocking NP trafficking

**Figure 3:**
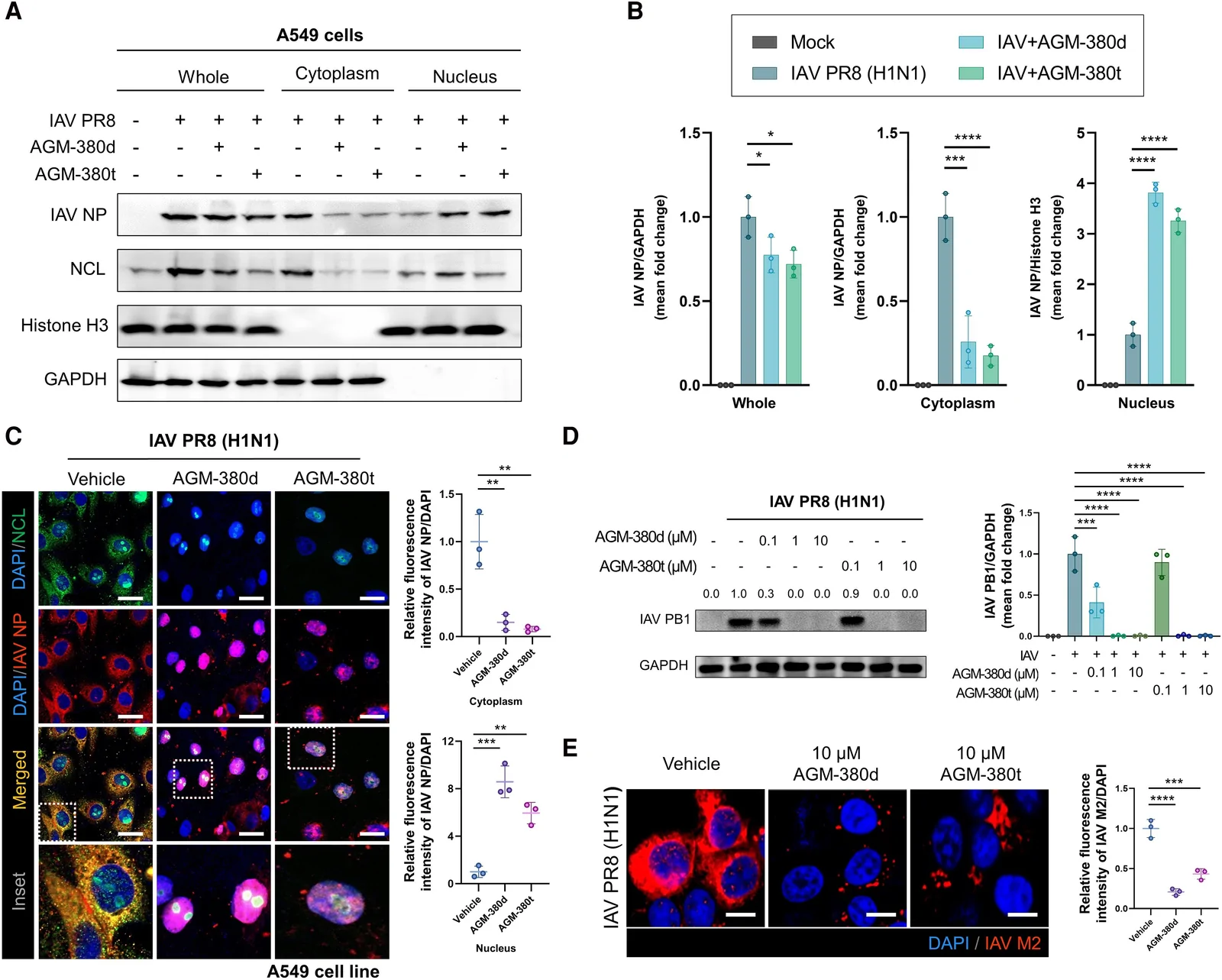
AGM-380d and AGM-380t inhibit IAV replication by blocking the nuclear trafficking of viral NPs. A, B) Representative western blot images and their graphical summaries. A549 cells infected with the IAV PR8 (H1N1) strain (MOI = 1 FFU) are treated with AGM-380d or AGM-380t at 10 µM and harvested 12 hpi. Western blot analysis is used to evaluate the levels of IAV NP and NCL in whole cells and in the nuclear and cytoplasmic fractions. Histone H3 and GAPDH served as loading controls for nuclear and cytoplasmic fractionation. Western blot data for IAV NP protein levels in whole-cell, nuclear, and cytoplasmic fractions are presented as graphs. The levels of IAV NP in the IAV-infected, mock-treated control, IAV-infected, AGM-380d-treated group, and IAV-infected, AGM-380t-treated group are normalized to GAPDH for the whole cell and cytoplasmic fractions, and to histone H3 for the nuclear fraction. In each graph, the reduction in NP protein level from treatment with AGM-380d or AGM-380t is compared with the NP level in IAV-infected, mock-treated controls, and the relative reduction is shown. C) Representative confocal images (left) of IAV NP inside cells and quantification of cytoplasmic (top right) and nuclear IAV NP expression levels (bottom right), with or without treatment with AGM-380d or AGM-380t. Treating cells infected with the IAV PR8 strain with AGM-380d or AGM-380t at 10 µM resulted in NPs remaining in the nucleus, whereas in virus-infected, vehicle-treated controls, NPs were distributed throughout the cytoplasm. The results in the graph (right) are expressed as relative fluorescent intensity, calculated as the mean pixel intensity of positively stained areas in virus-infected groups normalized to the mock-treated, mock-infected control. Bar = 10 µm. D) Representative western blot images and quantification of IAV polymerase basic 1 (PB1) demonstrating the antiviral effects of AGM-380d and AGM-380t. A549 cells infected with IAV PR8 (H1N1) strain (MOI = 1) or mock infected are treated with various concentrations of AGM-380d or AGM-380t as indicated. Inhibitory effects are evaluated using a western blot assay with antibodies specific for IAV PB1. The intensity of each target protein band is normalized to GAPDH as an internal loading control, and the relative values are presented as mean fold changes. E) Representative confocal images demonstrating the antiviral effects of AGM-380d and AGM-380t. A549 cells infected with IAV PR8 (H1N1) strain (MOI = 1) or mock infected are treated with 10 µM of AGM-380d or AGM-380t. Inhibitory effects are evaluated using an immunofluorescence assay with antibodies specific for the IAV matrix-2 ion channel protein (M2). The results in the graph (right) are expressed as relative fluorescent intensity, calculated as the mean pixel intensity of positively stained areas in virus-infected groups normalized to the mock-treated, mock-infected control. Bar = 5 µm. All data in the graphs are expressed as mean ± SD from three independent experiments. *P < 0.05, **P < 0.01, ***P < 0.001, and ****P < 0.0001; one-way ANOVA with Tukey's correction for multiple comparisons.

Next, we investigated whether AGM-380d and AGM-380t inhibit the transport of IAV and IBV NPs by targeting NCL. Treating IAV-infected A549 cells with either AGM-380d or AGM-380t decreased IAV NP levels in the cytoplasmic fraction but increased NP levels in the nuclear fraction compared with the mock-treated control. These results indicated that IAV NPs were retained in the nucleus (Figs. 3A, B and S8A, B). These effects were confirmed through confocal microscopy images (Figs. 3C and S8C). Retaining IAV NPs in the nucleus by treatment with AGM-380d or AGM-380t led to suppressed IAV protein synthesis, genome replication, and virus titers (Figs. 3C–E and S8C–E). Similar effects were observed in IBV-infected cells treated with AGM-380d or AGM-380t (Fig. S9). Furthermore, we evaluated the efficacy of AGM-380d and AGM-380t against IAV strains resistant to conventional viral-protein-targeting drugs. Oseltamivir resistance in pandemic H1N1 viruses is primarily conferred by a histidine-to-tyrosine substitution at residue 275 (the H275Y mutation) in the NA protein (35). Although 20 µM oseltamivir carboxylate significantly inhibited the replication of the susceptible (A/California/04/2009 [H1N1]) strain, it failed to suppress the H275Y-mutant–resistant strain (A/California/07/2009 [H1N1]; Fig. S10). In contrast, 10 µM of either AGM-380d or AGM-380t significantly reduced the viral titers of both strains, demonstrating robust antiviral potency regardless of oseltamivir susceptibility (Fig. S10). Taken together, these findings demonstrate that AGM-380d and AGM-380t exert broad-spectrum antiviral activity against both IAV and IBV—including drug-resistant strains—by specifically blocking the nuclear export of viral NPs.

Next, we assessed the broad-spectrum antiviral effects of AGM-380d and AGM-380t. To determine the selective index (SI) of AGM-380d and AGM-380t for each target virus, we first analyzed dose–response curves for the IC_50_ and CC_50_ against seven targeted RNA viruses. AGM-380d and AGM-380t showed IC_50_ values at lower micromolar concentrations for all seven viruses, ranging from 1.12 ± 0.01 µM (AGM-380d for IAV H9N2) to 9.76 ± 0.98 µM (AGM-380d for PSaV; Figs. S11 and S12). Additionally, the CC_50_ values for AGM-380d and AGM-380t were significantly higher than their IC_50_ values, ranging from 88.7 ± 1.9 µM (AGM-380t for porcine reproductive and respiratory syndrome virus [PRRS] in MARC-14 cells) to 107.5 ± 2.0 µM (AGM-380d for SARS-CoV-2 and porcine epidemic diarrhea virus [PEDV] in Vero E6 cells; Figs. S11 and S12 and Tables S1 and S2). As a result, the calculated SI, obtained by dividing CC_50_ by IC_50_, was high (Figs. S11 and S12 and Tables S1 and S2). These results demonstrate that both AGM-380d and AGM-380t provide broad-spectrum safety and antiviral activity against seasonal and pandemic influenza, as well as various RNA virus infections, at low concentrations.

### Protection of mice from lethal infection of IAV by AGM-380d and AGM-380t

**Figure 4:**
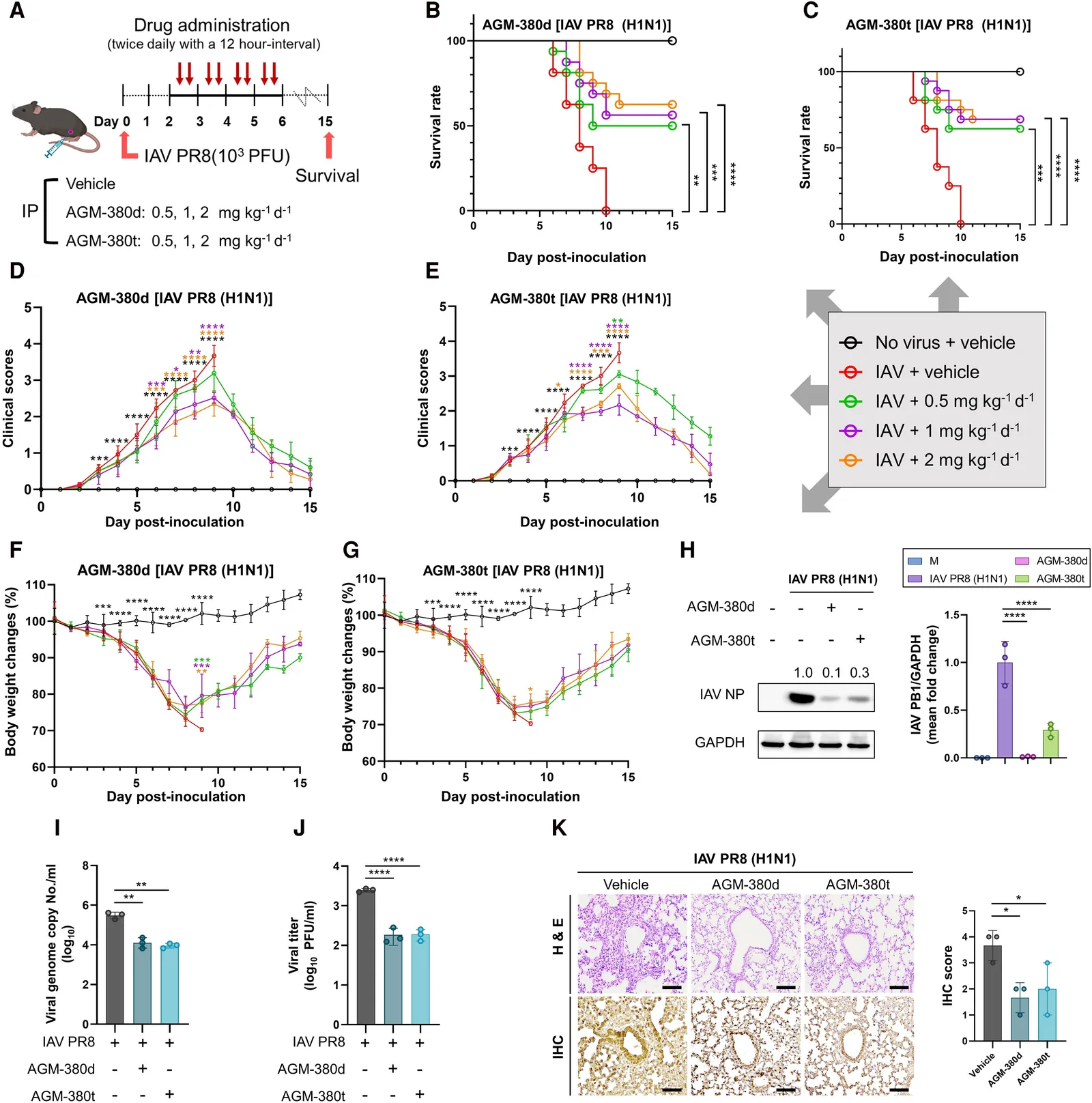
In vivo antiviral effects of AGM-380d and AGM-380t against IAV. A) The administration scheme of AGM-380: Mice (*n* = 16) are challenged with 10^3^ PFU of mouse-adapted IAV PR8 (H1N1) strain, followed by twice-daily administration of AGM-380 for four consecutive days, starting 2 days after the challenge. B, C) Survival rates (shown as percentages) of IAV-challenged mice treated with either AGM-380d (B) or AGM-380t (C). D, E) Daily clinical scores of surviving IAV-challenged mice treated with AGM-380d (D) or AGM-380t (E) at various doses. F, G) Daily body weight of surviving IAV-challenged mice treated with AGM-380d (F) or AGM-380t (G) at different doses. H) Representative western blot images (left) and quantification (right) of IAV NP expression levels in lungs sampled at 6 days postinfection from IAV-challenged mice (*n* = 3) with 10^3^ PFU of the mouse-adapted IAV PR8 strain and treated with AGM-380d or AGM-380t. The intensity of each target protein band is normalized to GAPDH as an internal loading control, and the relative values are presented as mean fold changes. I, J) Graphical representation showing changes in viral genome copy number (I) and titers (J) in lungs sampled at 6 days postinfection from IAV-challenged mice (*n* = 3) with 10^3^ PFU of the mouse-adapted IAV PR8 strain and treated with AGM-380d or AGM-380t. Viral genome copy number determined by RT-qPCR is normalized to GAPDH, and changes in the treated groups are compared with those in the mock-treated control. Viral titers determined by a plaque-forming assay showed relative changes in the infected groups compared with mock-infected controls. K) Representative images showing histological lesions and antigen distribution in lungs sampled at 6 days postinfection from IAV-challenged mice (*n* = 3) with 10^3^ PFU of the mouse-adapted IAV PR8 strain and treated with AGM-380d or AGM-380t. Amelioration of histological lung lesions (upper panels) and inhibition of IAV replication (lower panels) observed with AGM-380d or AGM-380t. The results in the graph (right) are expressed as IHC scores, calculated as the mean pixel intensity of positively stained areas in virus-infected groups normalized to the mock-treated, mock-infected control. Scale bar = 200 µm. Results are expressed as means ± SD. *P < 0.05, **P < 0.01, ***P < 0.001, and ****P < 0.0001; one-way ANOVA with Tukey's correction for multiple comparisons.

The safety of AGM-380d and AGM-380t was assessed by administering the maximum dose (2 mg kg^−1^ day^−1^) into the mouse abdominal cavity. No clinical symptoms, such as weight loss or gross organ abnormalities (liver, lungs, kidneys, heart, and spleen), were observed, even after giving the maximum dose (Figs. S13A and S14). Mice were challenged with 10^3^ PFU of the mouse-adapted IAV PR8 strain and treated with either the vehicle or the varying doses (0.5, 1.0, and 2.0 mg kg^−1^ day^−1^) of AGM-380d or AGM-380t (Fig. 4A). All IAV-challenged, vehicle-treated control mice died within 10 dpi. Treatment with AGM-380d and AGM-380t increased survival rates in a dose-dependent manner, with a maximum of 60% at 2 mg kg^−1^ day^−1^ of AGM-380d and 65% at 1 and 2 mg kg^−1^ day^−1^ of AGM-380t (Fig. 4B and C). Furthermore, following treatment, IAV-challenged mice exhibited a gradual improvement in clinical scores and a reduction in body weight loss (Fig. 4D–G). These findings demonstrate that AGM-380d and AGM-380t protect mice against lethal IAV infection.

Next, we evaluated whether AGM-380d and AGM-380t reduce lung lesions and improve survival by inhibiting pulmonary viral replication. Compared with IAV-challenged control mice, administering AGM-380d or AGM-380t to IAV-infected mice significantly decreased viral protein synthesis, viral genome replication, and progeny production (Fig. 4H–J). We evaluated whether AGM-380d and AGM-380t reduce lung lesions and improve survival by inhibiting pulmonary viral replication. Compared with IAV-challenged control mice, administering AGM-380d or AGM-380t to IAV-infected mice significantly decreased viral protein synthesis, viral genome replication, and progeny production (Fig. 4H–J). Furthermore, to monitor viral clearance kinetics, we analyzed viral shedding over a 15-day period. While the vehicle-treated control group exhibited high viral loads, mice treated with AGM-380d or AGM-380t showed a marked and progressive reduction in both viral genome copy numbers and infectious titers starting from 4 to 15 dpi (Fig. S15).

IAV-challenged control mice exhibited proliferation of type II pneumocytes and interstitial fibroblasts, along with increased presence of alveolar and interstitial macrophages, infiltration of some lymphoid cells and neutrophils into the interstitium, alveolar, and bronchiolar lumens, as well as edema and hyaline membrane formation (Fig. 4K). Treatment with AGM-380d or AGM-380t significantly reduced these IAV-induced lung lesions compared with vehicle treatment (Fig. 4K). Additionally, both treatments significantly decreased the number of IAV antigen-positive cells in the lungs (Fig. 4K). Our results demonstrate that administering AGM-380d or AGM-380t protects mice from lethal IAV infection by suppressing viral replication in the lungs.

### The antiviral synergy of AGM-380d and AGM-380t with IAV NA-targeting oseltamivir

Combining virus-targeting and host-directed antiviral therapy could synergistically improve safety and effectiveness (36–41). Therefore, we compared the safety and survival rates of a combination therapy (2 mg kg^−1^ day^−1^ of AGM-380d or AGM-380t with 2 mg kg^−1^ day^−1^ of IAV NA-targeting oseltamivir) with those of each agent alone (Fig. S16A). Administering each drug alone or together did not cause any abnormal clinical symptoms, including weight loss (Fig. S13B). Administering AGM-380d, AGM-380t, or oseltamivir at 2 mg kg^−1^ day^−1^ to IAV-challenged mice resulted in survival rates of 60, 65, and 40%, respectively (Fig. S16B and C). The combined therapy of 2 mg kg^−1^ day^−1^ AGM-380d and 2 mg kg^−1^ day^−1^ oseltamivir achieved a 90% survival rate among IAV-challenged mice (Fig. S16B). Remarkably, combined therapy of 2 mg kg^−1^ day^−1^ AGM-380t and 2 mg kg^−1^ day^−1^ oseltamivir resulted in a 100% survival rate (Fig. S16C). Using a combination therapy of AGM-380d or AGM-380t with oseltamivir improved IAV-induced clinical scores and body weight loss compared to single-agent treatments (Fig. S16D–G). Moreover, combination therapy resulted in a significant reduction in viral genome replication, progeny production, and IAV-induced lung lesions compared with monotherapy with AGM-380d or AGM-380t (Fig. S16H–J). Our results suggest that combining host-directed AGM-380t with virus-targeted oseltamivir enhances antiviral activity in vivo to a greater extent than either treatment alone (36–41).

## Discussion

Peptides and oligonucleotides are valuable therapeutic agents, but their low transmembrane efficiency often hinders their ability to reach effective intracellular concentrations (42, 43). Addressing this delivery challenge, especially for membrane-impermeable molecules, is crucial, as exemplified by the need to deliver AGM-330. To overcome this hurdle, we engineered novel CPP-conjugated antiviral drugs targeting NCL: the dimeric AGM-380d and the tetrameric AGM-380t (43). Their specific interactions with the N-terminus of NCL inhibited the transport of IAV and IBV NPs from the nucleus to the cytoplasm, demonstrating significant therapeutic potential against IAV and IBV infections (25).

As the efficacy of current influenza therapies is increasingly compromised by the rapid evolution of resistance among circulating strains, developing host-directed therapeutics (HDTs) has become a critical priority (6–8, 13, 44, 45). Our results show that both AGM-380d and AGM-380t confer robust antiviral activity in vitro and in vivo against diverse IAV and IBV strains, including oseltamivir-resistant isolates. Notably, the breadth of activity across multiple strains suggests that these peptides target a fundamental process essential for viral replication that is not readily bypassed by viral mutations. This broad-spectrum efficacy, together with the observed potentiation when co-administered with oseltamivir, supports the designation of AGM-380d and AGM-380t as promising HDTs for both seasonal outbreaks and future pandemic threats. Taken together, these findings indicate that targeting the NCL–NP axis is a viable strategy to overcome the limitations of current antiviral therapies (9–12).

The enhanced antiviral efficacy of the tetrameric AGM-380t compared with the dimeric AGM-380d likely stems from its increased binding avidity toward NCL. Multimerization of host-binding peptides is a proven strategy to strengthen target engagement (46); here, the tetrameric configuration provides more robust interference with NCL–NP trafficking by facilitating higher-order molecular interactions. This is consistent with previous findings (33), in which NCL-binding peptides exhibited nanomolar affinity, suggesting that the increased valency in AGM-380t further stabilizes the peptide–host complex, leading to the observed increase in potency in both in vitro and in vivo models (46).

Although the host protein NCL is evolutionarily conserved and regulated by multiple genes (14, 15), resistance can still develop at low levels following prolonged drug exposure (45). The mechanisms of this resistance may involve the virus using alternative host factors or enhancing its efficiency in response to selective pressure from host-directed drugs (45). To mitigate the emergence of resistance to the NCL-directed AGM-380d and AGM-380t, two primary strategies can be employed (6, 13, 34, 44, 45, 47). One highly effective strategy is combination therapy, pairing AGM-380d or AGM-380t with a virus-targeting antiviral agent. Our findings support this approach: the combination therapy of 2 mg kg^−1^ day^−1^ AGM-380t and 2 mg kg^−1^ day^−1^ oseltamivir provided complete protection against lethal IAV infection in mice. Another strategy is to use antiviral agents that target different host factors (45). This approach reduces the likelihood of viruses switching to a single alternative host factor or becoming more efficient under a single selective pressure, thereby making it significantly harder for resistance to develop (45).

NCL is a multifaceted host factor that plays a vital role at multiple stages of the life cycles of various RNA and DNA viruses (14, 16–32, 47). In this study, we demonstrated that treating IAV- or IBV-infected cells with either AGM-380d or AGM-380t led to a robust, significant reduction in NCL expression levels, accompanied by its nuclear confinement. These findings, consistently observed across both immunofluorescence assays and western blot analyses, suggest that our peptides effectively deplete the functional NCL pool available for viral exploitation. We propose two primary mechanisms for the resulting inhibition of viral replication. First, the marked reduction in NCL abundance likely disrupts the machinery required for the nuclear-to-cytoplasmic trafficking of IAV and IBV NPs, as evidenced by the significant nuclear retention of NP. Second, it is highly plausible that AGM-380d and AGM-380t directly abrogate the physical interaction between NP and NCL, further impeding viral assembly and export. Given that NCL is involved in diverse processes—including serving as an entry receptor (47), modulating late gene expression (31), and influencing nucleolar stress-induced apoptosis (30)—our NP-binding peptides may exert their antiviral effects by interfering with these multiple NCL-mediated pathways. The broad-spectrum efficacy of these peptides against seven different viruses, despite their disparate life cycles, further underscores the fundamental and conserved roles NCL plays across various viral taxa (16–32). While the specific steps hijacked by each virus may differ—ranging from genome replication and morphogenesis to trafficking and egress—their shared reliance on functional NCL makes them highly sensitive to NCL-targeting interventions (14). Furthermore, although our 3-(4,5-dimethylthiazol-2-yl)-2,5-diphenyltetrazolium bromide (MTT) assays showed no significant cytotoxicity, we cannot entirely rule out the induction of subtle nucleolar stress or early-stage apoptosis, which NCL is known to modulate. Whether the antiviral potency of AGM-380d and AGM-380t is partly mediated through the induction of such stress-related pathways remains to be elucidated in future studies. Our ongoing research aims to further dissect how these peptide drug candidates modulate these complex host–viromic interactions to achieve broad-spectrum inhibition.

## Materials and methods

### Cells and viruses

The cells used in this study included human lung carcinoma A549, African green monkey kidney epithelial MARC-145, and human colorectal adenocarcinoma HRT-18G cells cultured in Dulbecco's Modified Eagle Medium (DMEM); African green monkey kidney epithelial Vero E6 and porcine kidney epithelial LLC-PK cells in Eagle's Minimum Essential Medium (EMEM); and African green monkey kidney epithelial MA104 cells in Alpha Minimum Essential Medium (α-MEM). All cell cultures were supplemented with 10% fetal bovine serum, 100 U/mL penicillin, and 100 μg/mL streptomycin, then maintained at 37 °C in a humidified atmosphere with 5% CO_2_. Four strains of IAV with two strains of IBV, as well as six different RNA viruses, were utilized in this study. Detailed information on cell and virus culture, as well as virus titration, is provided in Tables S3–S5.

### Reagents, antibodies, siRNAs, and kits

Details of the reagents, antibodies, siRNAs, and kits used in this study are provided in Tables S6 and S7.

### Peptide synthesis

All peptides were synthesized by HLB Pep (Gwangju, Korea) using solid-phase peptide synthesis and Steglich esterification, respectively. The detailed procedures for peptide synthesis are provided in the SI Appendix.

### Treatment of cells with inhibitory peptides against NCL

The cell lines described above were used to evaluate intracellular levels of NCL and FITC-conjugated AGM-330d, AGM-380d, and AGM-380t, and to analyze individual viral proteins, the colocalization between viral NP and NCL, viral genome copy numbers, and virus titers, as described below. The detailed procedures are provided in the SI Appendix.

### Cytotoxicity assessment

The cytotoxicity of each peptide inhibitor and small molecule was evaluated using the MTT assay. The detailed procedure for this assay is available in the SI Appendix.

### Transfection of siRNA

A549 cells at 70 and 80% confluence were transfected with 1 or 10 nmol of siRNAs targeting NCL using Lipofectamine 3000 transfection reagent in 12-well culture plates or 8-well chamber slides, as previously described (48). For optimal knockdown of the target proteins, a second transfection was performed 24 h after the initial one. Subsequently, the cells were incubated for an additional 24 h. Scrambled siRNA (10 nmol) was transfected into cells using the same procedure as the negative control. Cells were infected with IAV PR8 or IBV Phuket and harvested at 18 or 24 hpi. Subsequent experiments used cell lysate and supernatant.

### Duolink proximity ligation assay

Direct interactions between NCL and the NPs of IAV or IBV were visualized in cells using the Duolink PLA kit (Sigma-Aldrich), as described elsewhere (49, 50). SI Appendix provides detailed procedures.

### Animals

All animal experiments were approved by the Institutional Animal Care and Use Committee (IACUC) of Chonnam National University (approval no. CNU IACUC-YB-2023-20) and were conducted in accordance with the approved guidelines and institutional regulations. All animals were cared for in accordance with current international laws and policies, as outlined in the NIH Guide for the Care and Use of Laboratory Animals (NIH Publication No. 85-23 [1985], revised 1996). The experimental design was designed to minimize both the number of animals used and their suffering. Details about the species, breeds, acclimation, and preparation for the study are provided in the SI Appendix.

### In vivo organ-specific toxicity

The organ-specific toxicity of each inhibitor in mice was evaluated using the methods outlined in Table S8.

### In vivo antiviral activity and pathogenicity

The method for evaluating the antiviral effects of AGM-380d and AGM-380t, alone or with oseltamivir, against IAV infection in a mouse model is described in Tables S9–S14.

### Plaque assay

The method for measuring the IAV viral titer using a plaque assay is described in the SI Appendix.

### Immunofluorescence assay

The NCL, FITC-conjugated AGM-330d, AGM-380d, or AGM-380t, and NPs of IAV and IBV in cultured cells or lung tissues were detected using immunofluorescence assays (50, 51). Detailed procedures are provided in the SI Appendix.

### Western blot analysis

The expression levels of target cellular and viral proteins were evaluated by western blot analysis in cultured cells or in mouse lung tissues (50, 51). The detailed procedure is described in the SI Appendix.

### Quantitative real-time PCR

Viral RNAs and host mRNAs in cultured cells and mouse lungs were quantified using real-time PCR, as detailed in Tables S15.

### Histopathology and immunohistochemistry

The histopathological changes and levels of IAV protein expression in the lungs of experimental mice were evaluated as previously described (52, 53) and as outlined in the SI Appendix.

### Immunoprecipitation assay

To identify direct interactions between NCL and IAV or IBV NPs, an immunoprecipitation assay was performed as previously described (50, 54). SI Appendix includes detailed procedures.

### Statistical analyses and software

Statistical analyses were performed in triplicate using one-way ANOVA with GraphPad Prism software version 10.3.1 (GraphPad Software Inc., La Jolla, CA, United States). *P* values <0.05 were considered statistically significant. Figures were created with Adobe Photoshop 2024 and Prism 10, version 3.1. Immunofluorescence data were analyzed using ZEN 2.6 (blue edition) and ZEN 2.3 SP1 (black edition) software (Carl Zeiss Microscopy, LLC, United States), and NIH ImageJ software (Fiji version) was used for statistical analysis of the images. For pathological analysis, NIH ImageJ software (version 1.48, Java 1.8.0) was used for image processing.

### Illustrations

Animal illustrations in Figs. 4 and S15 were created using BioRender software (https://biorender.com/).

## Supplementary Material

pgag269_Supplementary_Data

## Data Availability

All data are included in the manuscript or Supplementary Material.
