# Supplementary material for: Host nucleolin-targeting unique peptide drugs as potent broad-spectrum anti-influenza therapies
Source: PNAS Nexus. 2026 Sep 1;5(9):pgag269. doi: 10.1093/pnasnexus/pgag269 (PMC13531714; doi:10.1093/pnasnexus/pgag269)
Supplement: Supplementary file 1 [file pgag269_supplementary_data.pdf]

PNAS nexus

Supplementary Information for

Host Nucleolin-Targeting Novel Peptide Drugs as Potent Broad-Spectrum Anti-Influenza Therapies

Thu Ha Nguyen[a†], Muhammad Sharif[a†], Jae-Hyun Kim[b†], Yeong-Bin Baek[a], Mahmoud Soliman[c], Hyung-Jun Kwon[a,d], Seong-Hun Jeong[a,d], Ragab Farouk[a], Shaimaa Aboelmagd[a], Jae-Ha Ryu[e], Gyeong Min Kim[e], Woonsung Na[f], Don-Kyu Kim[a,g], Jeong-Sun Kim[a,h], Dong Ju Lee[a*], Jae Il Kim[b,e*] & Kyoung-Oh Cho[a*]

a. Host-Directed Antiviral Research Center, Chonnam National University, Gwangju 61186, South Korea.
b. School of Life Sciences, Gwangju Institute of Science and Technology, Gwangju 61005, South Korea.
c. Department of Pathology & Clinical Pathology, Faculty of Veterinary Medicine, Assiut University, Assiut, Egypt.
d. Functional Biomaterials Research Center, Korea Research Institute of Bioscience & Biotechnology, Jeongeup-si, Jeollabuk-do 56212, South Korea.
e. Pilot Plant, HLB PEP, Gwangju Technopark, Gwangju 61008, South Korea.
f. Department of Oral Microbiology and Immunology, and Dental Research Institute , School of Dentistry, Seoul National University, Seoul 03080, South Korea.

g. Department of Integrative Food, Bioscience, and Biotechnology, Chonnam National University, Gwangju 61186, South Korea.

h. Department of Chemistry, Chonnam National University, Gwangju 61186, South Korea.

†Thu Ha Nguyen, Muhammad Sharif, and Jae-Hyun Kim contributed equally to this work.

*Corresponding authors: E-mail: choko@jnu.ac.kr; Phone +82-62-530-2845; Fax: +82-62-530-0835, E-mail: jikim@gist.ac.kr; Phone: +82-62-715-2494; Fax: +82-62-715-2484, and E-mail: leedongj@jnu.ac.kr; Phone: +82-62-530-0836; Fax: +82-62-530-0835.

This PDF file includes:

SI Methods

SI Figures S1-S16

SI Tables S1 to S15

SI References

Supplementary Experimental Section

Cells and viruses

Madin-Darby canine kidney (MDCK), Madin-Darby bovine kidney (MDBK), human lung carcinoma A549 and Calu-3, African green monkey kidney epithelial Vero E6, MA104, and MARC-145, porcine kidney epithelial LLC-PK, and human colorectal adenocarcinoma HRT-18G and Caco-2 cells were obtained from the American Type Culture Collection (ATCC, Manassas, VA, USA). A549, MDCK, MDBK, MARC-145, and HRT-18G cells were cultured in Dulbecco's Modified Eagle's Medium (DMEM), Calu-3, Vero E6, and LLC-PK cells in Eagle's Minimum Essential Medium (EMEM), and MA104 cells in α-MEM. All media were supplemented with 10% fetal bovine serum (FBS), 100 U/mL penicillin, and 100 μg/mL streptomycin. In the experiments below, the cells mentioned above were used under contamination-free conditions, such as bacteria and fungi. Each cell and its culture conditions are summarized in the *SI Appendix*, Table S3. Various virus strains were obtained and cultured: four strains of IAV [Puerto Rico/8 (PR8) (H1N1), California/04/2009 (H1N1) (2009 Pandemic H1N1-like), oseltamivir-resistant California/07/2009 (H1N1) mutant [a histidine-to-tyrosine substitution at residue 275 (H275Y)], Brisbane/10/07 (H3N2), and Chicken/Korea/01310/2001 (H9N2)]; two strains of IBV [Austria/1359417/2021 (B/Victoria) and Phuket/3073/2013 (B/Yamagata)]; and strains of SARS-CoV-2 KCDC03, BCoV KWD20, PEDV QIAP1401, PRRSV LYM, RVA NCDV, and PSaV Cowden. The PR8 (H1N1) IAV strain was purchased from ATCC, and chicken/Korea/01310/2001 (H9N2) was kindly provided by Green Cross Veterinary Products. GC Biopharma Hwasun Plant donated the remaining IAV and IBV strains. SARS-CoV-2 and oseltamivir-resistant California/07/2009 (H1N1) strains were kindly supplied by the Korea Disease Control and Prevention Agency (Cheongju, South Korea), while PEDV QIAP1401 and PRRSV LYM strains were obtained from the Animal and Plant Quarantine Agency (Gimcheon, South Korea), and PSaV from Dr. K.O. Chang (Kansas State University). To promote viral growth, each respective medium was supplemented with specific substances: N-tosyl-L-phenylalanine chloromethyl ketone (TPCK)-treated trypsin for IAV, IBV, and SARS-CoV-2; porcine pancreatin for BCoV; porcine pancreatic trypsin for PEDV; crystallized trypsin for BCoV; or glycochenodeoxycholic acid (GCDCA) for PSaV. Detailed information on these substances is provided in the *SI Appendix*, Tables S4 and S5.

Titers of IAV and IBV strains were measured using plaque or cell culture immunofluorescence (CCIF) assays. The results were expressed as plaque-forming units per milliliter (PFU/mL) or fluorescence focus units per milliliter (FFU/mL). SARS-CoV-2 titers were determined using the 50% tissue culture infectious dose ($TCID_{50}$) or the CCIF assay, with a monoclonal antibody (Mab) specific for the SARS-CoV-2 nucleocapsid (N) protein, expressed as $TCID_{50}$/mL and FFU/mL, respectively. PSaV, PRRSV, RVA, PEDV, and BCoV titers were measured using the CCIF assay with Mabs specific for the BCoV spike (S) protein, PEDV nucleoprotein (N), RVA VP6 protein, PRRSV matrix (M) protein, or PSaV VPg protein, and were expressed as FFU/mL.

Reagents, kits, siRNAs, and antibodies

The *SI Appendix*, Table S6 lists the chemicals, kits, and siRNAs used in this study. Porcine pancreatic trypsin, TPCK-treated trypsin, oseltamivir, GCDCA, 3-(4,5-dimethylthiazol-2-yl)-2,5-diphenyl tetrazolium bromide (MTT), and Triton X-100, all purchased from Sigma-Aldrich, along with pancreatin and crystal trypsin from Gibco, were dissolved in PBS (pH 7.4) to prepare stock solutions. For nuclear staining, SlowFade™ Gold Antifade Mountant with 4',6-diamidino-2-phenylindole (DAPI) was obtained from Molecular Probes. For plaque assays, ultrapure agarose was sourced from Thermo Scientific. Kits used in this study include Bicinchoninic acid (BCA) protein assay and NE-PER™ Nuclear and Cytoplasmic Extraction Reagents from Thermo Scientific (Waltham, MA), RNeasy Mini Kit from Qiagen, TOPscript™ cDNA Synthesis Kit from Enzynomics, and Duolink PLA Kit from Sigma-Aldrich. siRNAs and Lipofectamine 3000 from Bioneer and Thermo Scientific, respectively, were used to silence the target gene.

The *SI Appendix*, Table S7 lists various unconjugated primary antibodies for each target protein or virus. MAbs include IAV membrane (M2) protein (Abcam), IAV NP (Abcam), IBV NP protein (Invitrogen, Seoul, South Korea), IAV M2 protein (Abcam), SARS-CoV-2 nucleocapsid (N) protein (Prosci), Nucleolin (NCL) (Cell Signaling), Histone (Abcam), GAPDH (Santa Cruz), β-actin (Thermo Scientific), BCoV spike (S) protein (Native Antigen Company), PEDV nucleoprotein (N) (Medgene Labs), and RVA viral structural protein 6 (VP6) (Median Diagnostic). Conversely, PAbs were used against SARS-CoV-2 S protein (Prosci),

IAV PB1 (Novus Biologicals), IAV hemagglutinin (HA) (Santa Cruz), IAV virion (Viasat), PRRSV matrix (M) protein (Bioss Antibodies), and PSaV viral genome-linked protein (VPg). HRP-conjugated secondary antibodies, including goat anti-rabbit IgG, rabbit anti-goat IgG, and goat anti-mouse IgG, were used for western blot and immunohistochemistry (IHC) applications. Alexa Fluor (AF) 594-conjugated donkey anti-rabbit IgG, AF488-conjugated donkey anti-rabbit IgG, AF594-conjugated goat anti-mouse IgG, AF488-conjugated goat anti-mouse IgG, and AF488 Phalloidin were used for an immunofluorescence assay (IFA).

Peptide synthesis

AGM-330 and a novel CPP (RIMRILRILKLAR) were synthesized using the solid-phase method of Fmoc chemistry, employing various blocking groups to protect amino acids. The synthetic peptide was deprotected and cleaved with reagent K (82.5% TFA, 2.5% 1,2-ethanedithiol, 5% H2O, 5% thioanisole, and 5% phenol, v/v). This was followed by precipitation with diethyl ether. Preparative RP-HPLC (Shimadzu) was performed using a Shimadzu LC-6AD system and a Shimadzu LC-10Avp system equipped with an ODS column (20 × 250 mm).

To synthesize AGM-380d and AGM-380t, each of AGM-330d and AGM-330t was mixed with a 5 mM solution of maleimide-RIMRILRILKLAR in DMF (5 equivalents) and 0.1% v/v N, N-diisopropylethylamine (DIPEA; Duksan). The resulting mixture was stirred at room temperature. After the reaction, the product was purified using RP-HPLC. FITC-conjugated peptides were synthesized with FITC-5-maleimide (Thermo Fisher Scientific) under the same conditions. The structure and purity of the synthetic peptides were confirmed through analytical RP-HPLC and MALDI-TOF mass spectrometry analysis.

Cytotoxicity assessment

The cytotoxicity of each peptide inhibitor against NCL was assessed using an MTT assay according to established protocols (1-3). Each peptide inhibitor was serially diluted twofold with the vehicle and added to the maintenance medium of each cell monolayer. Subsequently, the MTT substrate was applied at a concentration of 0.3 µg/mL, and the absorbance was

measured at 570 nm. Percent cell viability was calculated using the following formula: [(ODsample - ODblank) / (ODcontrol - ODblank)] × 100%. Dose-response curves for cell cytotoxicity were generated, and the half-maximal cytotoxic concentration (CC_{50}) was determined using GraphPad Prism software version 10.3.1.

Duolink proximity ligation assay (DPLA).

In situ interactions between NCL and IAV NP or IBV NP were detected using the Duolink PLA kit (Sigma-Aldrich), following established protocols (4, 5). Briefly, A549 cells grown on 8-well chamber slides were infected with either the IAV PR8 strain or the IBV Phuket strain (MOI = 1). After incubation for 12 hours, the cells were fixed with 4% paraformaldehyde in PBS for 15 minutes and permeabilized with 0.2% Triton X-100 for 10 minutes at 20°C. The cells were then treated with Duolink blocking solution and incubated in a pre-warmed, humidified chamber at 37 °C for 30 minutes. After two washes with Duolink washing buffer A for 5 minutes each, the cells were incubated overnight at 4°C with primary antibodies targeting mouse IAV NP, IBV NP, or NCL, or with a control rabbit antibody against the Na^+/K^+-ATPase β1 protein. After two additional washes with Duolink washing buffer A for 5 minutes each, the cells were incubated with secondary antibodies conjugated to oligonucleotides (PLA probes anti-rabbit MINUS and anti-goat PLUS) for 1 hour at 37°C in a preheated, humidified chamber. Excess PLA probes were washed off with two 5-minute washes in Duolink washing buffer A. The Duolink ligation solution was then applied to the slides for 30 minutes at 37°C in a preheated humidity chamber, followed by two 2-minute washes with Duolink washing buffer A. Next, the Duolink amplification-polymerase solution was added to the slides in a dark, humidified chamber maintained at 37°C for 100 minutes. The slides were washed twice with 1x Duolink washing buffer B for 10 minutes each, followed by a final 1-minute wash with 0.01x Duolink washing buffer B. Finally, the cells were mounted with Duolink in situ mounting medium containing DAPI and observed with an LSM 800 confocal microscope (Carl Zeiss). PLA signals appeared as red fluorescent dots.

Treatment of cells with inhibitory peptides and chemicals

The above cell lines, cultured in 6- or 12-well plates or 8-well chamber slides to reach the

desired confluency, were washed twice with phosphate-buffered saline (PBS, pH 7.4). Next, they were mock-inoculated with medium alone or infected with IAV, IBV, SARS-CoV-2, BCoV, PEDV, RVA, PRRSV, and PSaV at various multiplicities of infection (MOI): 0.1, 1, or 5 FFU per cell. After a 1-hour absorption period for each inoculum, the cells were washed twice with PBS (pH 7.4). Following virus absorption, they were treated with different concentrations (0.1 μM, 1 μM, or 10 μM) of peptide inhibitors—AGM-330, AGM-380d, and AGM-380t. To confirm the binding of FITC-conjugated AGM-380d or AGM-380t to NCL, A549 cells were transfected with siRNA targeting NCL or a scrambled siRNA. Subsequently, they were treated with FITC-conjugated AGM-380d or AGM-380t. The binding of NCL to these conditions was assessed using the IFA described below. These treatments were applied to cells grown in 6- or 12-well plates or 8-well chamber slides at the desired confluency and incubated for the specified time, as outlined in the previous instructions (1). The cells were used to evaluate intracellular levels of NCL, as well as FITC-conjugated AGM-330d, AGM-380d, and AGM-380t, and to analyze individual viral proteins, the interaction between viral NP and NCL, viral genome copy numbers, and virus titers, as described below.

Experimental animals

We used female wild-type (WT) C57BL/6J mice (7 weeks old) obtained from Samtako (Osan, South Korea), as described previously (1). The mice were housed in standard cages with free access to food and water. They were kept in a specific-pathogen-free facility under a 12-hour light/dark cycle at 25°C and 40–55% humidity. After one week of acclimation, the mice were used for the experiments described below.

***In vivo* organ-specific toxicity**

Three groups of three anesthetized mice were treated intraperitoneally with either AGM-380d or AGM-380t in a 50 μL vehicle [10% PEG400 in water (vol/vol)] containing 0.1, 1, or 2 mg/kg/day BID with a 12-hour interval for 4 days straight (*SI Appendix*, Table S8). Gross changes in the lungs, liver, kidneys, heart, and spleen of the mice in each group were assessed following procedures described below.

Determination of median lethal dose (LD_{50}) of the mouse-adapted PR8 strain

Mouse-adapted PR8 (H1N1) strain was developed using previously described methods (1, 6, 7). Briefly, female BALB/c mice (6 weeks old) were intranasally inoculated with 50 μL of cell-passaged supernatant of the IAV PR8 strain under appropriate anesthesia (Zoeltil/Xylazine). Lung samples were collected at 4 days post-infection (dpi), homogenized, and then 50 μL of the centrifuged homogenate, after syringe filtration (0.2 μm), served as the inoculum for the following passage. After the ninth passage, a single clone from the lung homogenate was selected and purified through plaque purification in MDCK cells. The cloned virus was then passaged once in the allantoic cavities of 12-day-old chicken eggs for 48 hours at 37°C to produce the virus stock.

The median lethal dose (LD_{50}) of the mouse-adapted PR8 strain was determined as described previously (8-10). Four groups of 12 mice were infected with 10^1–10^4 PFU of the PR8 strain, and their survival rates and body weights were recorded daily for 15 days. The LD_{50} was calculated by generating dose-response curves using GraphPad Prism software version 10.3.1.

In vivo antiviral activity and pathogenicity

The antiviral effects of AGM-380d and AGM-380t on IAV infection were evaluated in a mouse model, with 5 groups of 16 mice each. Some mice were mock-challenged, while others were challenged once via intranasal inoculation with 10^3 PFU of the mouse-adapted PR8 strain in 50 μL PBS. Starting at two dpi, they received intraperitoneal injections of AGM-380d or AGM-380t in 50 μL of vehicle [10% PEG400 in water (vol/vol)] at doses of 0.5, 1.0, or 2.0 mg/kg/day, administered BID with a 12-hour interval, for four consecutive days (*SI Appendix*, Tables S9 and S10). Each group of animals was monitored daily for changes in body weight, clinical signs, and survival over a 15-day period.

The synergistic anti-IAV effects of AGM-380d or AGM-380t, combined with oseltamivir, were studied in 7 groups of 16 anesthetized mice. The mice were challenged once with an intranasal inoculation of 10^3 PFU of the mouse-adapted PR8 strain in 50 μL PBS. Afterwards,

they received either a vehicle, AGM-380d, or AGM-380t in 50 µL via intraperitoneal injection, or oseltamivir in 50 µL by oral gavage (using a 19-gauge needle) (*SI Appendix*, Tables S11 and S12).

The levels of viral proteins and genomic copy numbers, changes in host gene mRNA levels, and histopathological alterations in lung tissues were evaluated in four groups, each consisting of five 8-week-old female WT C57BL/6J anesthetized mice. First, the animals were intranasally inoculated with PBS or 10^3 PFU of the mouse-adapted IAV PR8 strain. Afterward, they received either the vehicle AGM-380d or AGM-380t (*SI Appendix*, Tables S13 and S14).

During necropsy, lung tissues from the mice were prepared for further analysis. The left cranial lobe was halved and fixed in 10% neutral formalin for histopathology. The remaining left lobes were combined, thoroughly minced, and homogenized using a Precellys 23 tissue homogenizer (Bertin Technologies, France). After centrifugation at 4°C, the resulting supernatants were prepared in separate buffers or reagents according to the manufacturer's instructions or previous methods (1), including determination of infectious virus titer, viral and cellular target protein expression levels, and viral genome and cellular mRNA expression levels.

Plaque assay

As previously reported, the effects of AGM-380d and AGM-380t, or siRNA transfection, on IAV or IBV infectivity were evaluated using a plaque assay (8, 11). Briefly, test supernatants were serially diluted tenfold in DMEM and inoculated into each well of 24-well plates containing confluent MDCK cells. After a 1-hour adsorption period at 37°C, each well was washed three times with DMEM, then filled with 4 mL of agar overlay medium containing 1× DMEM, 1 µg/mL TPCK-treated trypsin, and 0.5% ultrapure agarose (Thermo Scientific). Plates were then incubated for 3 days at 37°C. Following fixation with 4% buffered paraformaldehyde (PFA) for 10 minutes and removal of the overlay medium, the cells were rinsed with PBS and stained with 0.1 mg/mL crystal violet for 30 minutes at 20°C. Plaque numbers were counted using the endpoint dilution method. Subsequently, the 50% inhibitory concentration (IC_{50}) and selectivity index were calculated using GraphPad Prism software version 10.3.1.

Median Tissue culture infectious dose ($TCID_{50}$) assay

The SARS-CoV-2 titer in the samples was measured using a $TCID_{50}$ assay as previously described (2). In brief, Vero E6 cells in 96-well plates were infected with 10-fold serial dilutions of the test samples for 1 hour. The medium was then replaced with DMEM containing 1 μg/mL TPCK-treated trypsin. After 4 days of incubation at 37°C, virus titers were calculated using the Reed and Muench method (12).

Immunofluorescence assay (IFA)

In multiple in vitro experiments, we performed IFA to characterize the expression of cellular or viral proteins in the presence of inhibitor treatment or siRNA transfection, as described above (13). Each permissive cell line was seeded in an 8-well chamber slide, transfected with specific or scrambled siRNA, infected with or without the target virus, and treated with each inhibitor or vehicle. After incubation, the cells were fixed in 4% PFA and permeabilized with 0.2% Triton X-100. Antibodies against each target protein in PBS (pH 7.4) with 5% BSA were applied overnight at 4°C. Subsequently, each secondary antibody conjugated with AF488, AF594, or AF647 was incubated for 1–2 hours at 25°C. Nuclei were stained with SlowFade Gold antifade reagent containing 1× DAPI solution (Molecular Probes). The slides were examined using an LSM 800 confocal microscope with Zen Blue Software 2.6 (Carl Zeiss; Jena, Germany). The data were analyzed using ZEN 2.6 (blue edition) and ZEN 2.3 SP1 (black edition) software. NIH ImageJ Fiji was used for statistical analysis of the images.

To determine the titer of each target virus in the samples under various conditions, such as the application of each inhibitor or siRNA, we prepared pre-cleared supernatants from cells or tissue homogenates in a 10-fold serial dilution in virus growth medium. Each diluted sample was transferred into the corresponding wells of 96-well plates and incubated for 1 hour. After virus adsorption, the cells were replaced with fresh virus growth medium and further incubated at 37°C in a CO_2 incubator until the scheduled time. Subsequently, the cells were fixed in 4% PFA and permeabilized with 0.2% Triton X-100. Primary antibodies specific to IAV M2, SARS-CoV-2 N, BCoV S, PEDV N, RVA VP6, PRRSV M, or PSaV VPg proteins were applied to the corresponding cells. AF488-conjugated secondary antibodies, compatible with the

primary antibodies, were added for 1–2 hours at 25°C. The plates were examined using inverted fluorescence microscopy after washing and mounting with 60% glycerol. The endpoint dilution method counted the number of cells showing a positive signal for viral antigens. A dose-response curve was generated to determine IC_{50} and SI using GraphPad Prism software version 10.3.1.

Western blot analysis

Western blot analysis was performed to evaluate the expression levels of the target protein from in vitro and in vivo experiments, as previously described (2, 5, 13). In addition, the distribution of IAV and IBV NPs and NCL in the cytoplasm or nucleus was examined. Individual cytoplasmic and nuclear fractions and lysates from cells or lung tissues were prepared using RIPA buffer containing 10 mM Tris/HCl (pH 7.4), 100 mM NaCl, 1 mM EDTA, 1 mM EGTA, 1 mM NaF, 20 mM Na2P2O7, 2 mM Na3VO4, 1% Triton X-100, 10% glycerol, 0.1% SDS, and 0.5% deoxycholate (Invitrogen) on ice. After centrifugation at 12,000 × g for 10 min at 4°C, the total protein content in the clarified supernatant was measured using a BCA protein assay kit (Thermo Scientific). Protein samples were normalized and separated via SDS-PAGE at 90 mA/gel. Subsequently, proteins were transferred onto nitrocellulose membranes (GE Healthcare Life Sciences) at 150 mA/blot. The membranes containing the transferred proteins were incubated for 1 hour at 25°C with Tris-buffered saline containing 5% skim milk and 0.1% Tween-20. They were then incubated overnight at 4°C on a shaker with primary antibodies against each target protein. After incubation with horseradish peroxidase (HRP)-conjugated secondary antibody for 1 hour at 25°C, target proteins were visualized using an ECL detection reagent (Dogen, Seoul, South Korea). Visualization was performed with a Davinch-K Imaging System (Youngwha Scientific, Seoul, South Korea). The levels of each target protein were normalized to internal controls such as β-actin or GAPDH. Relative expression values were calculated independently in three separate experiments and presented as the mean fold change.

Quantitative real-time PCR

As previously described, quantitative real-time PCR was used to detect viral RNAs or host

mRNAs in cultured cells or lung tissue (11, 14). Total RNA was extracted from cells or tissue with the RNeasy kit (Qiagen), and cDNA was synthesized using the TOPscript™ cDNA Synthesis kit (Enzynomics). Then, real-time PCR was performed using TOPreal™ qPCR 2X PreMix from Enzynomics, along with a specific primer pair (*SI Appendix*, Table S15). The LineGene 9600 Plus Real-time PCR detection system (Bioer, Hangzhou, China) was used with these conditions: for viral genome copy numbers, DNA polymerase was activated at 95°C for 10 minutes, followed by 40 cycles of three steps: 95°C for 15 seconds, 60°C for 20 seconds, and 72°C for 20 seconds. For cellular target gene mRNA, DNA polymerase was activated at 95°C for 10 minutes, followed by 40 cycles of three steps: 95°C for 15 seconds, 55°C for 20 seconds, and 72°C for 20 seconds.

The cycle threshold (Ct) values were automatically determined based on the initial exponential phase and the maximum amplification rate. RNA levels were quantified relative to a reference using the comparative Ct method (15). Cellular mRNA was compared to a control group that was neither inoculated nor treated, and the results were reported as fold change. The reduction in viral load was expressed as relative values, comparing the viral genome copy number in the virus-infected, inhibitor-treated, or siRNA-transfected groups to that in the virus-infected, mock-treated, or scrambled siRNA-transfected controls.

Histopathology

Histopathological analysis was performed to assess the antiviral effect of peptide inhibitors in the lungs of mice, as previously described (16). Tissue samples from the experimental animals were fixed in 10% neutral formalin at 25°C for 3–4 days. After washing with tap water, the trimmed tissues were gradually dehydrated through 60%–100% ethanol, cleared with xylene, and embedded in a paraffin block using an automatic embedding machine. The paraffin block was then sectioned at 3 μm using a microtome and stained with H&E or IHC as previously described. H&E sections were examined under light microscopy, with morphological changes in each group reviewed in a blinded manner, as previously described (16).

IHC

We detected IAV protein in lung tissue from each experimental group to assess antiviral activity as described elsewhere (14). In summary, a 3-μm-thick tissue section was prepared on poly-L-lysine-coated slides and processed as previously detailed. After deparaffinization, the tissue sections were rehydrated and heated in 10 mM citrate buffer (pH 8.0) for antigen retrieval in an autoclave. Next, 3% H_2O_2 was applied for 30 minutes at 25°C to quench endogenous peroxidase activity. The slides were then incubated overnight at 4°C with primary antibodies against IAV virions, followed by 1–2 hours at the same temperature with HRP-conjugated secondary antibodies. Staining was performed using the Dako REAL™ EnVision™ Detection System (DakoCytomation, Denmark) with 3,3'-diaminobenzidine (DAB) to visualize antigens, following the manufacturer's instructions. Finally, the slides were lightly counterstained with hematoxylin, dehydrated, cleared in xylene, and mounted. The staining intensity of IHC slides was quantitatively evaluated using the IHC Profiler plugin in NIH ImageJ, which performs automated analysis of digital IHC images stained for cytoplasmic IAV virions or NCL. IHC Profiler generates a four-tier score (1–4), where 1 = negative, 2 = low positive, 3 = positive, and 4 = high positive. Statistical analyses were performed using GraphPad Prism software (version 10.3.1).

Immunoprecipitation assay

Each target protein was immunoprecipitated as previously described (5, 17). Briefly, A549 cells grown in 6-well plates were independently infected with different strains of IAV and IBV at an MOI of 10 FFU per cell, or mock-infected. They were treated with either AGM-380d or AGM-380t at 10 μM, then incubated for 12 or 24 hours at 37°C. Afterward, the cells were washed and lysed as previously described. The cell lysates were pre-cleared by incubating with protein A- or G-agarose beads for 30 minutes at 4°C. Next, the pre-cleared lysates were incubated overnight at 4°C with antibodies against NCL or IAV NP, or with an irrelevant antibody targeting the Na^+/K^+-ATPase β1 protein. The immune complexes were captured by incubating with A- or G-agarose beads for 1 hour at 4°C. The immunoprecipitated proteins were then analyzed by western blot using antibodies against IAV NP, IBV NP, or NCL, as previously described. Instead of using A- or G-agarose beads for capture, secondary antibodies specific to each primary antibody were added to each reaction. The immune complexes were

captured again with A- or G-agarose beads, and the resulting immunoprecipitated proteins were analyzed via western blot, as previously described.

Flow cytometry

To measure the expression levels of NCL on the surface of MDCK or A549 cells, which were either infected with the IAV PR8 strain at 3 MOI FFU/cell or left uninfected, we performed a flow cytometry assay as previously described (1). Briefly, mock-infected or virus-infected cells were harvested at 12 hpi by incubating them at 37°C for 5 minutes with Accutase™ Cell Detachment Solution (BD Biosciences, Franklin Lakes, NJ, USA), then washed with PBS and pelleted. After fixing with 4% PFA for 10 minutes, the cells were left unpermeabilized to assess surface NCL expression. The cells were incubated at 4°C for 15 hours with a 1:200 dilution of rabbit Mab against NCL (Cell Signaling Technology, Seoul, South Korea). Following two washes with PBS, the samples were stained with a goat Alexa Fluor 488-conjugated anti-rabbit IgG antibody (Invitrogen) at a 1:200 dilution as a secondary antibody and incubated at 37°C for 1 hour. Twenty thousand single cells from resuspended MDCK or A549 cells in 100 μL of cold PBS were initially examined based on size (Forward Scatter - FSC), granularity, or internal complexity (Side Scatter - SSC). Only cells with the appropriate properties were analyzed for fluorescence activity at 488 nm/530 nm (Excitation/Emission). All procedures were performed using the Attune™ NxT flow cytometer (Thermo Scientific), and data from each sample were digitized under the same measurement conditions with Attune™ NxT software v3.1.2.

Supplementary Figures and Their Legends

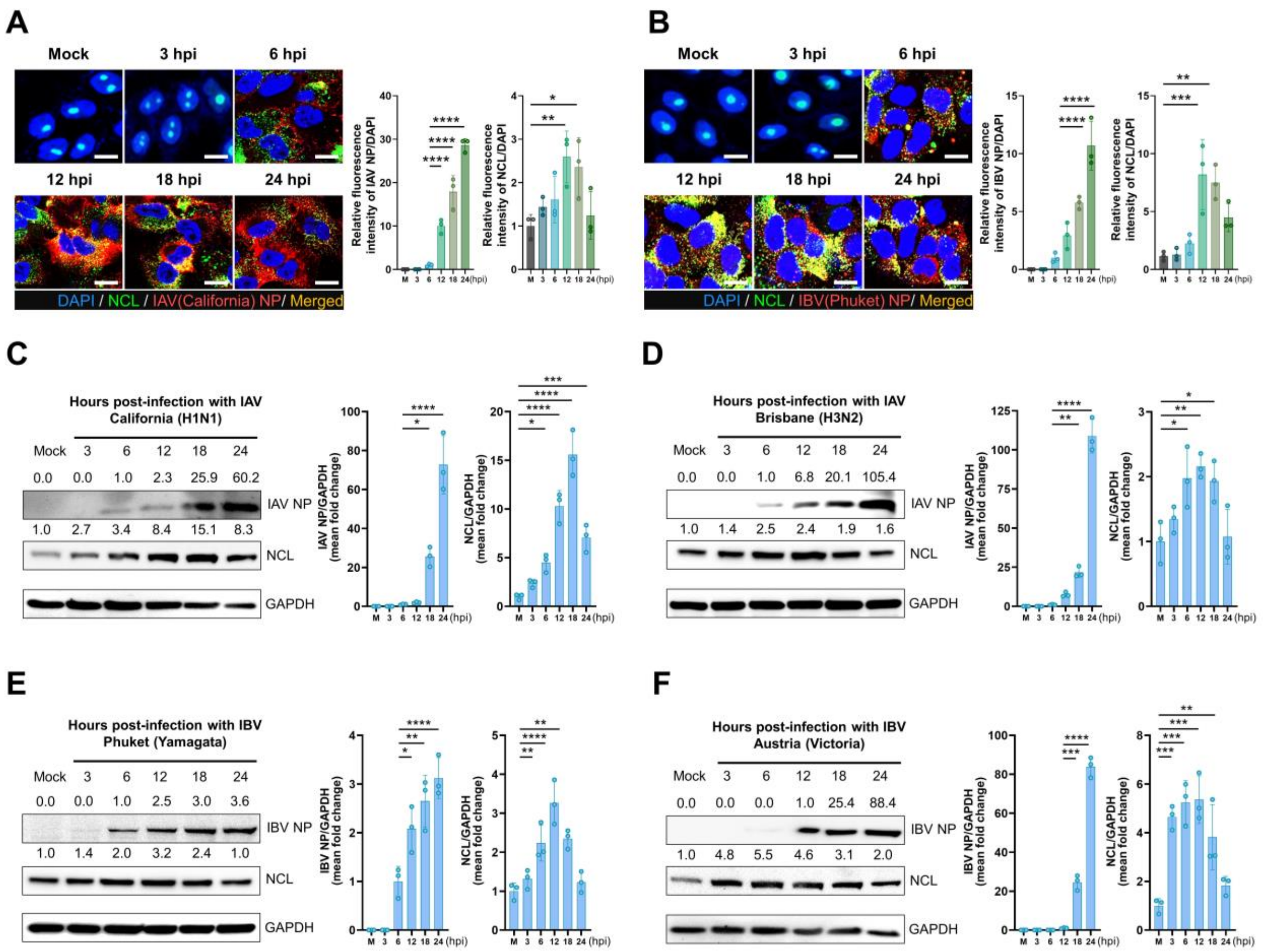

Fig. S1. Dynamics of nucleolin (NCL) expression in response to infection with different strains of influenza A virus (IAV) and influenza B virus (IBV). (A, B) Representative confocal images showing NCL (green) colocalized with the nucleoprotein (NP, red) of IAV (A) or IBV (B) in human adenocarcinoma A549 cells infected with the human pandemic influenza A/California/2009 (H1N1) strain or human influenza B/Phuket/3073/2013 (Yamagata) strain (MOI = 1 FFU) at various time points. The results in each graph are expressed as relative fluorescence intensity, calculated as the mean pixel intensity of positively stained areas in virus-infected groups normalized to the mock-treated, mock-infected control. Bar = 5 µm. (C-F) Representative western blot images. The levels of NCL in A549 cells infected with A/California/2009 (H1N1), A/Brisbane/10/07 (H3N2), B/Phuket/3073/2013 (Yamagata), or B/Austria/1359417/2021 (Victoria) strains increased up to 12 or 18 hours post-infection and then decreased. IAV NP levels were also measured in the same samples to examine the

correlation between NCL and IAV replication. The intensity of each target protein band was normalized to GAPDH as an internal loading control, and the relative values are presented as mean fold changes.

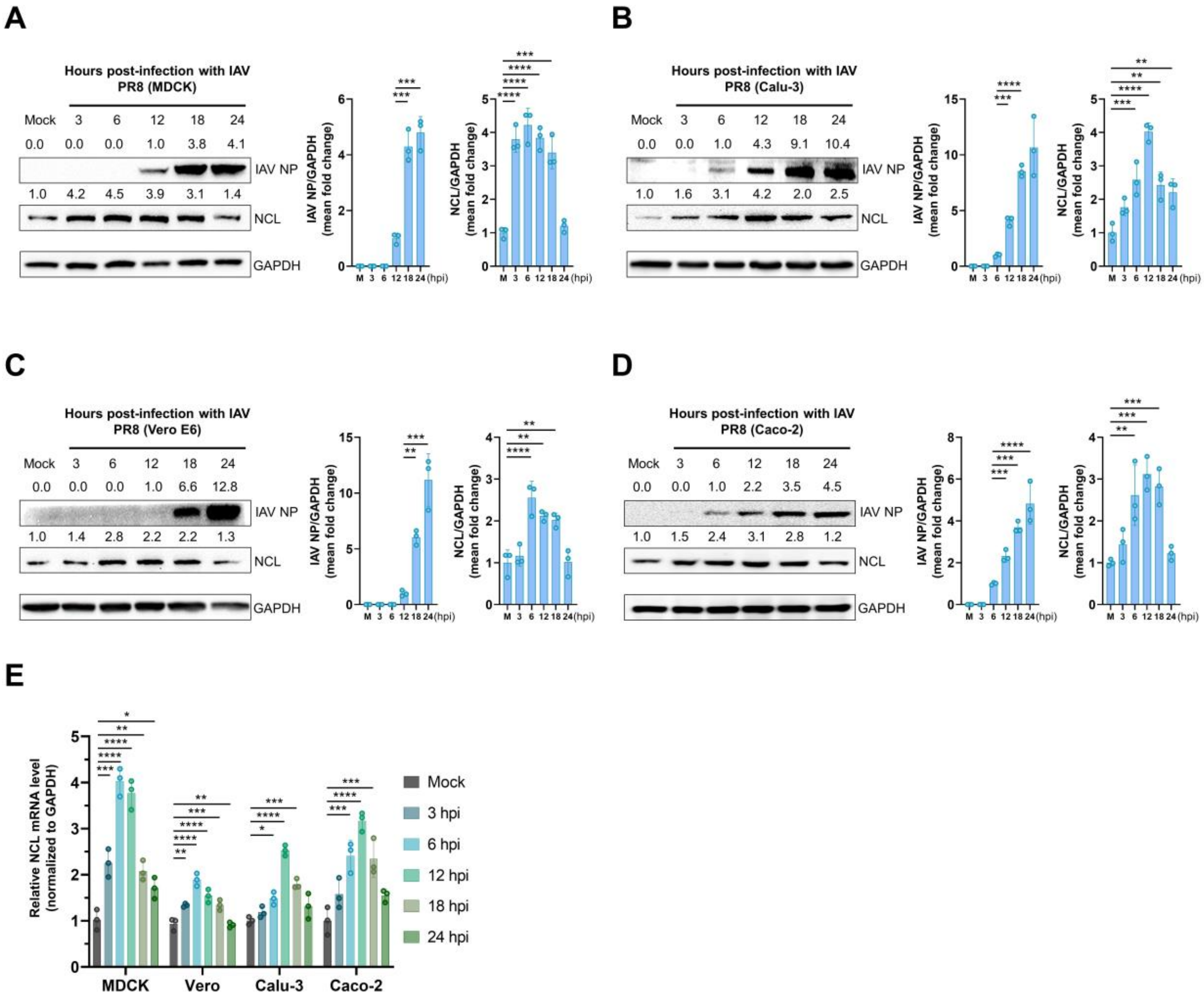

Fig. S2. ***In vitro*** **and** ***in vivo*** **dynamics of nucleolin (NCL) expression in the different cells infected with influenza A virus (IAV) Puerto Rico/8 (PR8) (H1N1) strain.** (A-D) Representative western blot images showing the sequential changes of NCL at various time points in response to infection with IAV PR8 (H1N1) strain (MOI = 10 FFU) in canine kidney MDCK (A), human lung adenocarcinoma Calu-3 (B), African green monkey kidney Vero E6 (C), and human colorectal adenocarcinoma Caco-2 (D) cells. The expression level of IAV NP was also detected in the same samples to assess the correlation between NCL and IAV replication. The intensity of each target protein band was normalized to GAPDH as an internal loading control, and the relative values are presented as mean fold changes. (E) Graphical representation showing relative NCL mRNA levels at various time points in different cell lines infected with IAV PR8 (H1N1) strain. RT-qPCR results were normalized to the housekeeping gene GAPDH, and relative changes in IAV-infected groups were compared with the mock-

infected control. Results in the graph are presented as arithmetic means ± standard deviation. *$P < 0.05$, **$P < 0.01$, ***$P < 0.001$, one-way ANOVA with Tukey's correction for multiple comparisons.

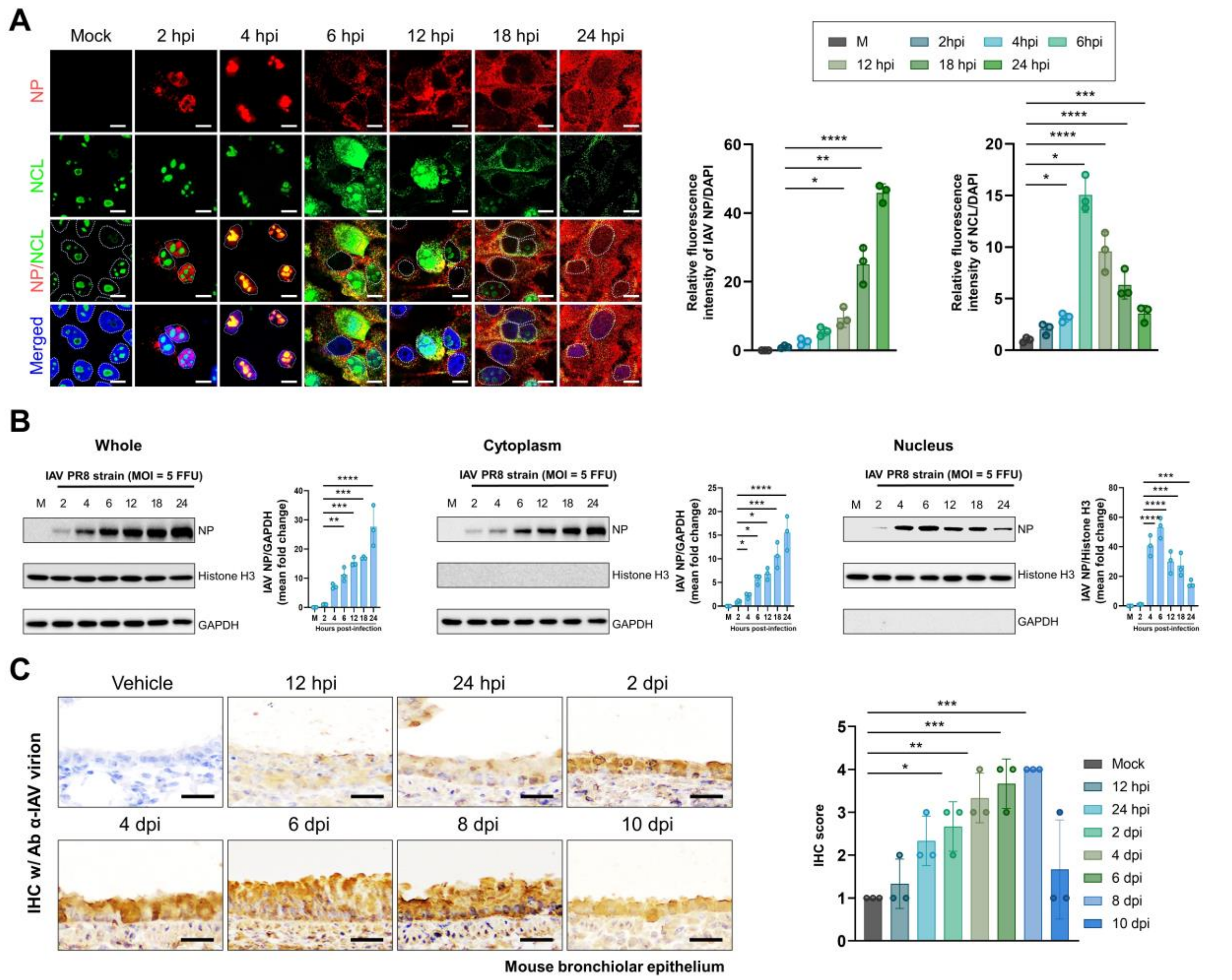

Fig. S3. Dynamics of nucleoprotein (NP) and nucleolin (NCL) in A549 cells infected with influenza A (IAV) Puerto Rico (PR8) (H1N1) strain and in IAV-infected mice. (A) Representative confocal image (left) and quantification (right) of sequential changes in NCL (green) colocalized with IAV nucleoprotein (NP, red) in human adenocarcinoma A549 cells infected with the IAV PR8 strain (MOI = 5 FFU) at different time points. Dashed circles indicate nuclei. In the merged images, nuclei are stained with DAPI. Bar = 7 μm. All images are representative of three independent experiments. The results in the graph (right) were expressed as relative fluorescent intensity, calculated as the mean pixel intensity of positively stained areas in virus-infected groups normalized to the mock-treated, mock-infected control. (B) Representative western blot images. A549 cells infected with IAV PR8 (H1N1) (MOI = 5 FFU) were harvested at various time points as indicated. Western blot analysis was used to evaluate the levels of IAV nucleoprotein (NP) and nucleolin (NCL) in whole cells and in the nuclear and cytoplasmic fractions. Histone H3 and GAPDH served as loading controls for

nuclear and cytoplasmic fractionation. Western blot data for IAV NP protein levels in whole-cell, nuclear, and cytoplasmic fractions are presented as graphs. The levels of IAV NP were normalized to GAPDH for the whole cell and cytoplasmic fractions, and to histone H3 for the nuclear fraction. (C) IAV virion expression in the bronchiolar epithelium of mice infected (n = 3) with 10^3 PFU of the mouse-adapted IAV PR8 strain at different days post-inoculation (dpi), detected by immunohistochemistry using an antibody against IAV virion with hematoxylin counterstaining. The results in the graph (right) are expressed as IHC scores, calculated as the mean pixel intensity of positively stained areas in virus-infected groups normalized to the mock-treated, mock-infected control. Bar = 50 μm.

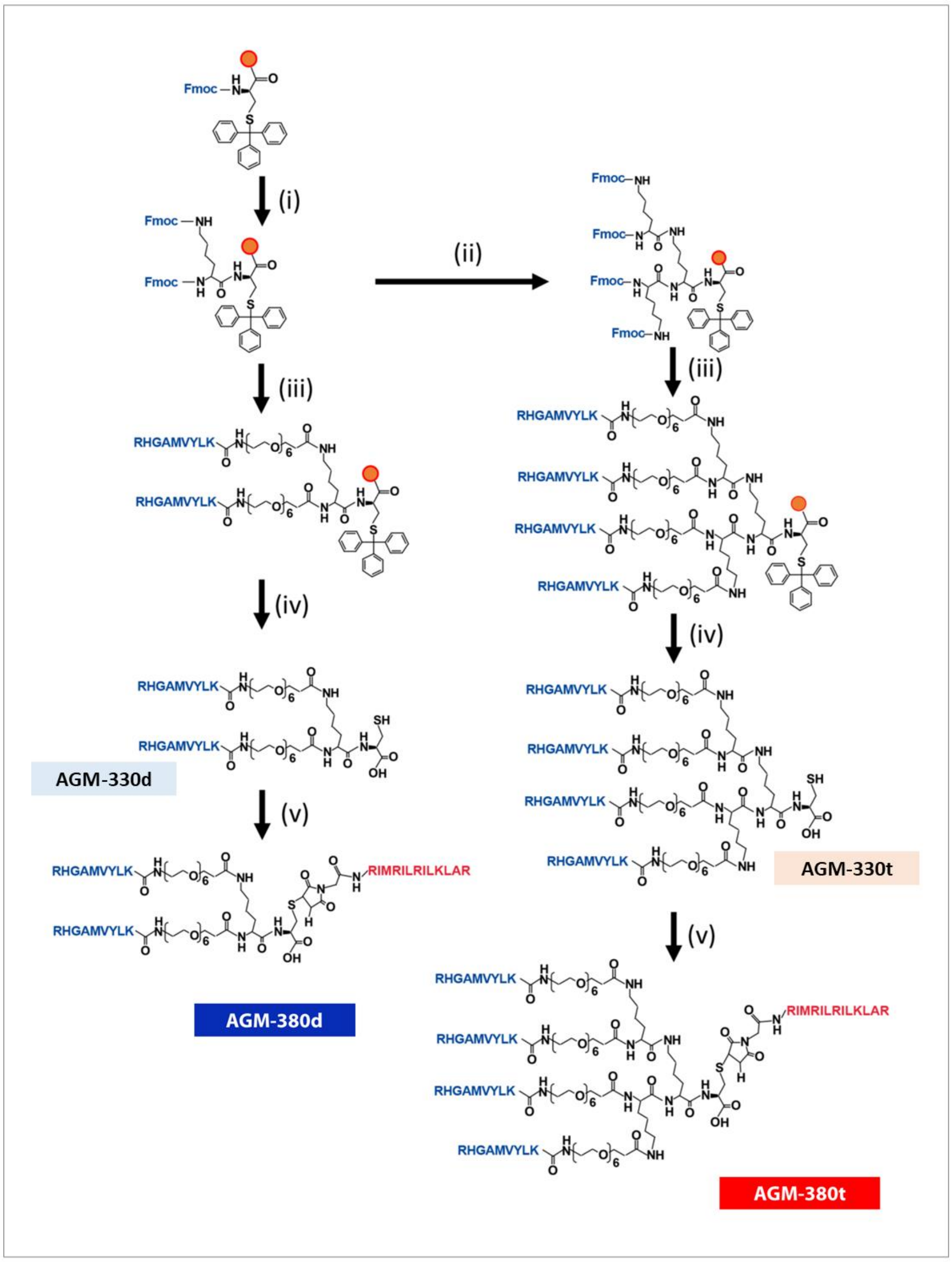

Fig. S4. Schematic diagram for the synthesis of AGM-380d and AGM-380t. Procedure for synthesizing both AGM-380d and AGM-380t. Note on reagents and conditions: (i) Fmoc-Lys-(Fmoc)-OH, piperidine, DMF; (ii) Fmoc-Lys-(Fmoc)-OH, piperidine, DMF; (iii) RHGAMVYLK-OH, piperidine; (iv) reagent K; (v) maleimide-RIMRILRILKLAR, DMF. Fmoc = 9-fluorenylmethoxycarbonyl, DMF = dimethylformamide. The blue letters indicate nucleolin-binding peptides, and the red letters indicate cell-penetrating peptides (CPP).

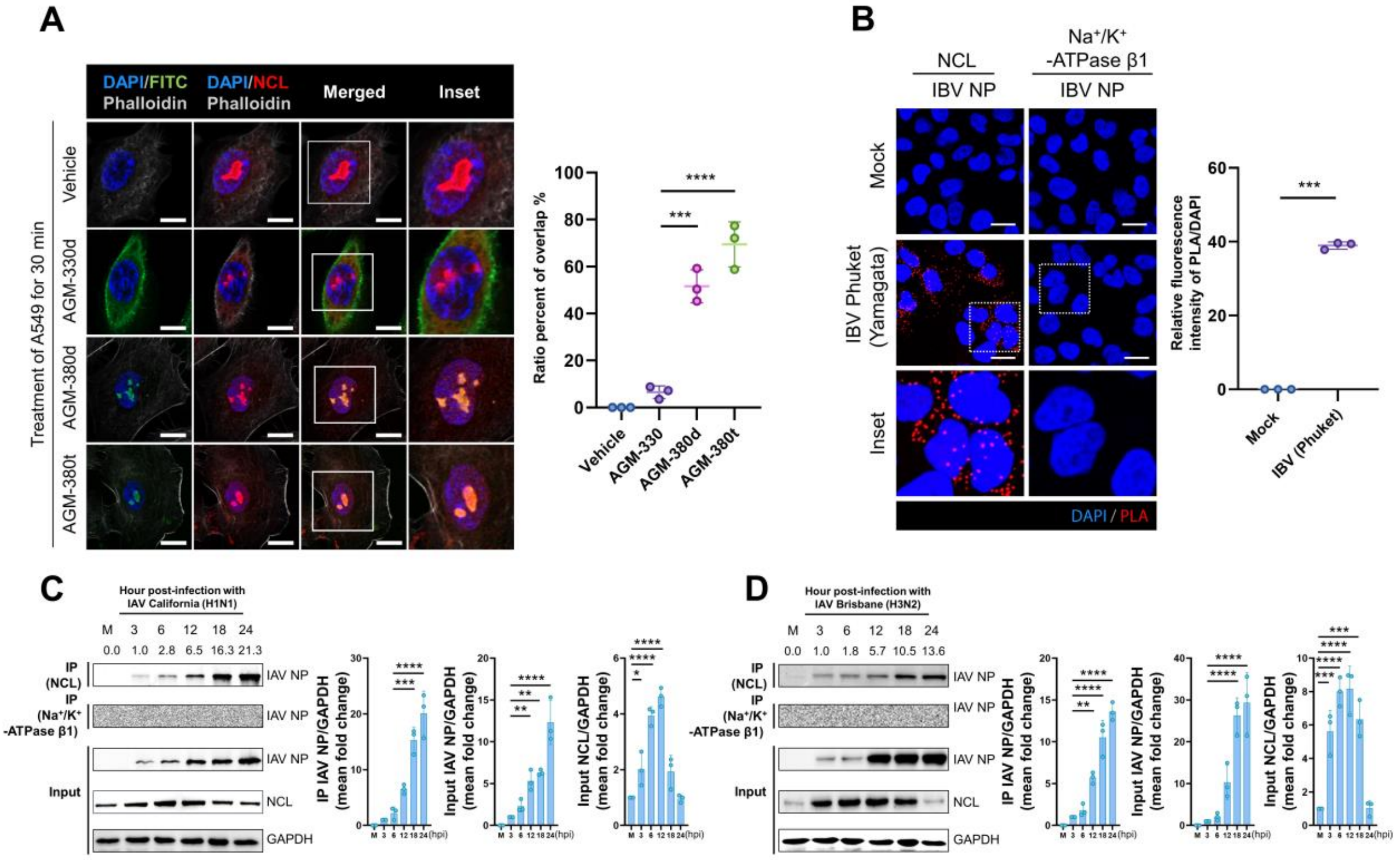

Fig. S5. Direct interaction of nucleolin (NCL) with IAV and IBV nucleoprotein (NP). (A) Representative images showing FITC-conjugated AGM-330, AGM-380d, and AGM-380t in A549 cells. Each peptide was used at a concentration of 2.5 µM and incubated with A549 cells for 30 minutes. The actin cytoskeleton (white) was stained with Alexa Fluor 488-labeled phalloidin, and nuclear DNA was stained with DAPI (blue). The results in the graph (right) are expressed as the percent overlap ratio between intranuclear NCL and each FITC-conjugated peptide, calculated as the mean pixel intensity of positively stained areas in virus-infected groups, normalized to the mock-treated, mock-infected control. Bar = 5 µm. (B) Representative images illustrating the direct interaction of NCL with IBV NP [Phuket/3073/2013 (Yamagata lineage)] (MOI = 1 FFU) or an irrelevant Na^+/K^+-ATPase β1 protein as a negative control at 12 hours post-infection using the Duolink proximity ligation assay. Positive signals appear as red dots. The results in the graph are expressed as relative fluorescence intensity, calculated as the mean pixel intensity of positively stained areas in virus-infected groups normalized to the mock-treated, mock-infected control. Bar = 10 µm. (C, D) Representative results of immunoprecipitation assays. To determine the interaction between NCL and NP of IAV, A549 cells infected with human pandemic IAV California/04/2009 (H1N1) or human epidemic IAV Brisbane/10/07 (H3N2) strains (MOI = 10 FFU) are harvested at 3, 6, 12, 18, and 24 hours post-infection (hpi) and immunoprecipitated with an antibody against NCL or with an

irrelevant antibody targeting the Na^+/K^+-ATPase β1 protein as a negative control. IAV NP, NCL, and GAPDH, either immunoprecipitated or present in each input, are detected using their respective antibodies. The intensity of each target protein band is normalized to GAPDH as an internal loading control, and the relative values are expressed as mean fold changes. All data in the graphs are expressed as mean ± standard deviation from three independent experiments. *$P < 0.05$, **$P < 0.01$, ***$P < 0.001$, and ****$P < 0.0001$; one-way ANOVA with Tukey's correction for multiple comparisons.

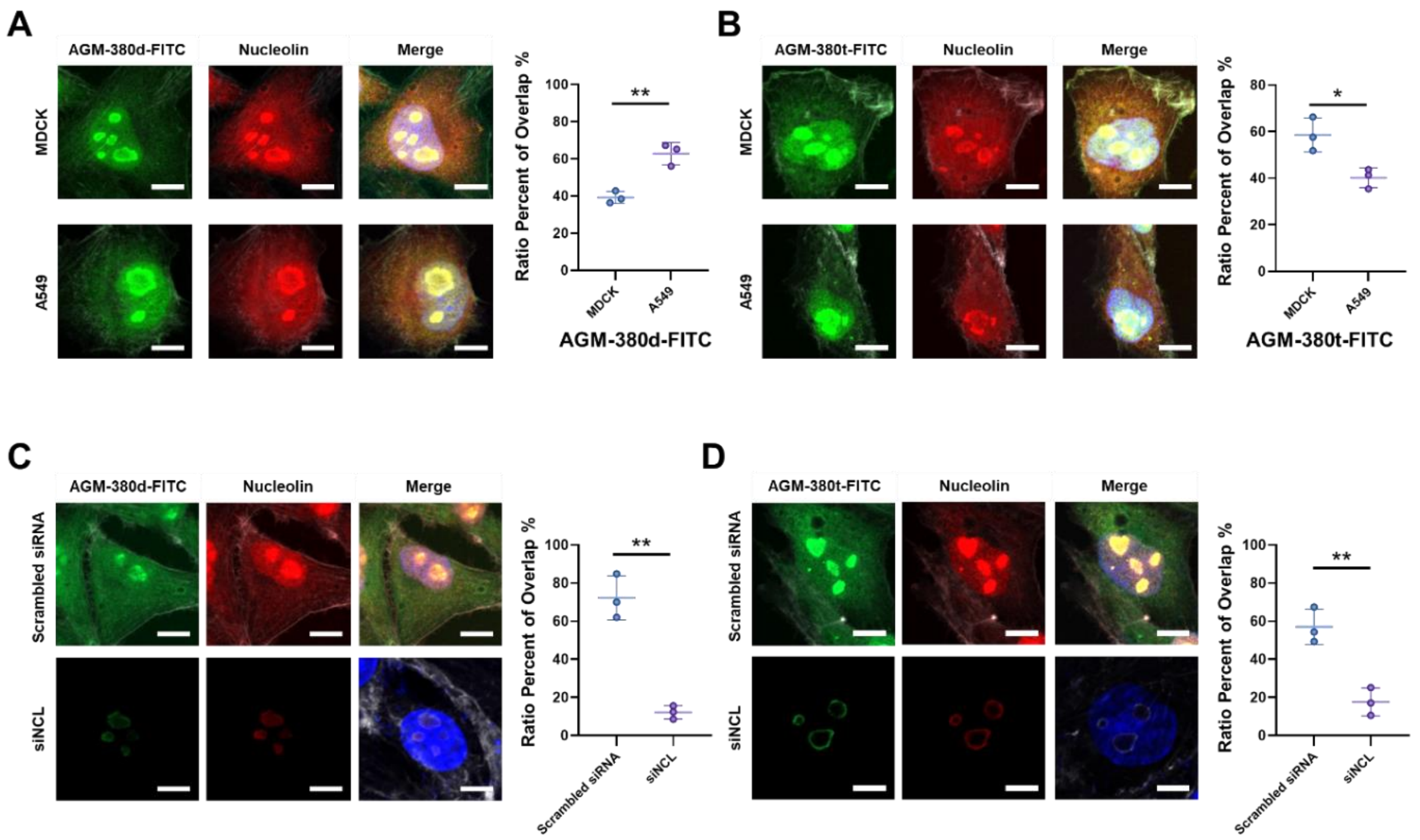

Fig. S6. Binding of FITC-conjugated AGM-380d or AGM-380t with nucleolin (NCL) in the cytoplasm and nucleus of MDCK or A549 cells. (A) Representative images showing FITC-conjugated AGM-380d in MDCK or A549 cells. Each peptide was used at a concentration of 2.5 μM and incubated with each cell line for 30 minutes. The actin cytoskeleton was stained with Alexa Fluor 488-labeled phalloidin (white), and nuclear DNA was stained with DAPI (blue). (B) Representative images showing FITC-conjugated AGM-380t in MDCK or A549 cells. Cells were treated as described in panels A and B. (C) Representative confocal images show colocalization of NCL (red) with FITC-conjugated AGM-380d (green) in A549 cells transfected with scrambled siRNAs or siRNAs against NCL, repeated twice before treatment with FITC-conjugated AGM-380d. (D) Representative confocal images display colocalization of NCL (red) with FITC-conjugated AGM-380t (green) in A549 cells transfected with scrambled siRNAs or siRNAs against NCL, repeated twice before treatment with FITC-conjugated AGM-380t. NCL, shown in red, was detected using a primary rabbit polyclonal antibody followed by a secondary Alexa Fluor 488-labeled donkey anti-rabbit antibody. Actin filaments (white) were stained with Alexa Fluor 488-labeled phalloidin, and nuclear DNA was stained with DAPI (blue). The results in the graphs (right) are expressed as a ratio percent of overlap between intranuclear NCL and each FITC-conjugated peptide, calculated as the mean pixel intensity of positively stained areas in virus-infected groups normalized to the mock-treated, mock-infected control. Scale bars = 5 μm.

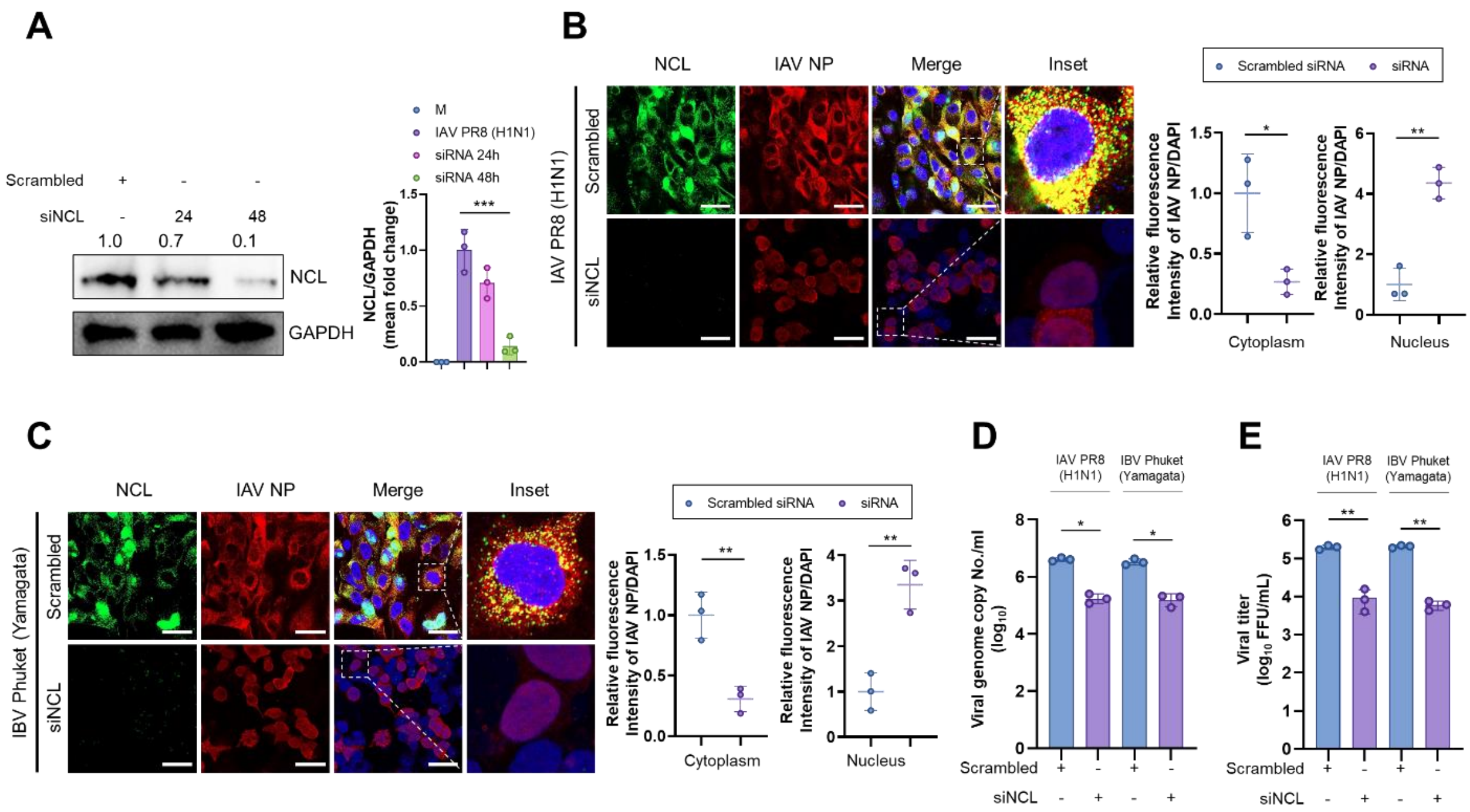

Fig. S7. Knockdown of nucleolin (NCL) inhibits the nuclear transport of influenza A virus (IAV) and influenza B virus (IBV) nucleoprotein (NP). (A) Representative western blot showing decreased NCL protein levels in A549 cells following NCL knockdown at 24 and 48 hours post-transfection. The intensity of each target protein band was normalized to GAPDH as an internal loading control, and the relative values are presented as mean fold changes. (B, C) Representative confocal images illustrating NCL (green) colocalized with viral nucleoprotein (NP, red) in A549 cells infected with IAV PR8 (H1N1) (B) and IBV Phuket/3073/2013 (Yamagata lineage) (C) strains at an MOI of 1 FFU, 12 hours post-infection. The results in the graph are expressed as relative fluorescence intensity, calculated as the mean pixel intensity of positively stained areas in virus-infected groups normalized to the mock-treated, mock-infected control. Scale bar = 2 µm. (D, E) Graphs depicting reduction of viral genome copies (D) and titers (E) in A549 cells transfected with siRNA against NCL or scrambled siRNA and then infected with either IAV PR8 (H1N1) or IBV Phuket/3073/2013 (Yamagata) strains at an MOI of 1 FFU, 24 hours post-infection. Data are shown as means ± standard deviation. *P < 0.05, **P < 0.01, ***P < 0.001, using one-way ANOVA with Tukey's correction for multiple comparisons.

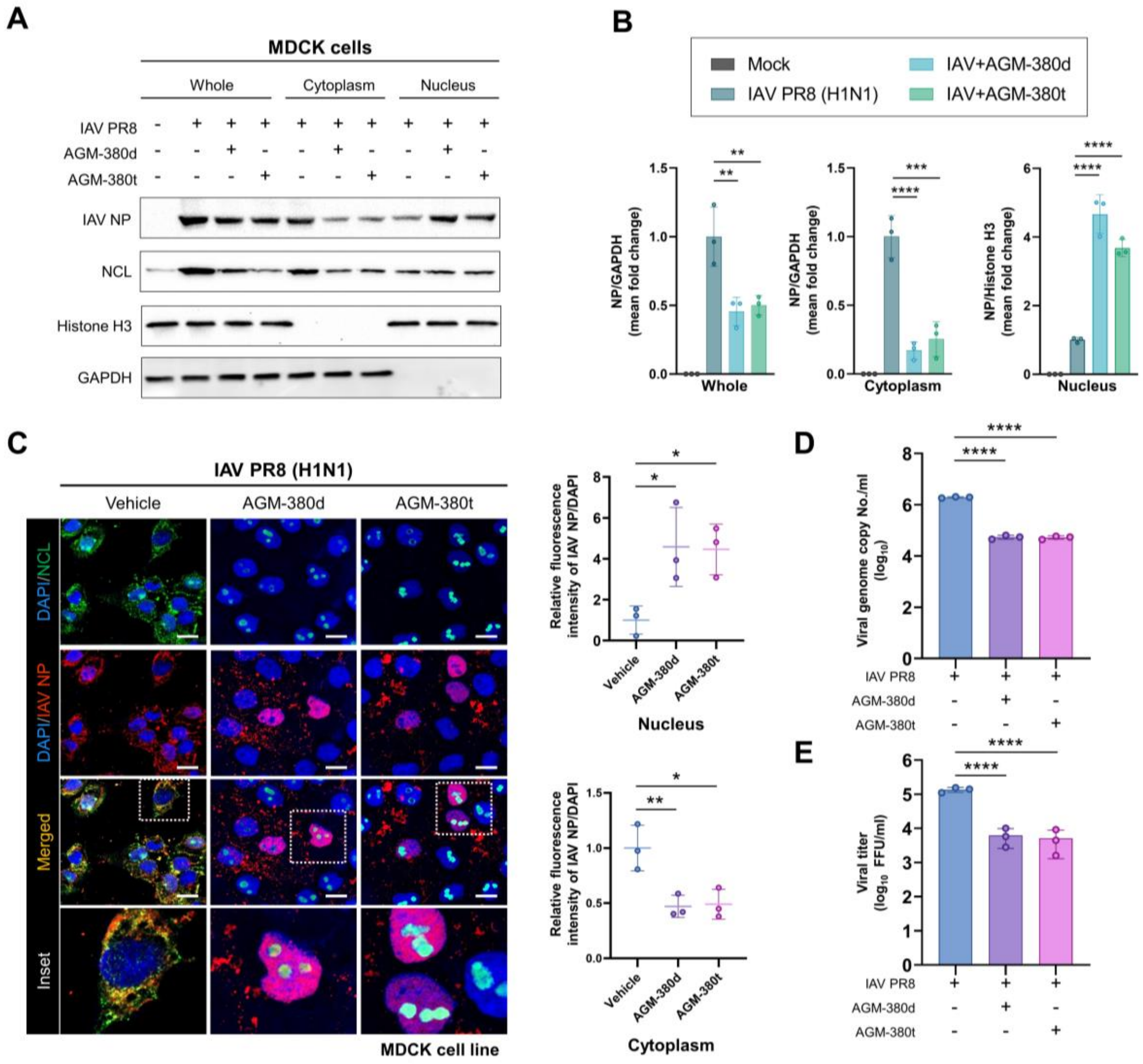

Fig. S8. Inhibitory effects of AGM-380d and AGM-380t on IAV by blocking nuclear trafficking of viral nucleoprotein (NP). (A, B) Representative western blot images and graphical summaries. MDCK cells infected with IAV PR8 (H1N1) strain (MOI = 1 FFU) were treated with 10 μM of either AGM-380d or AGM-380t and harvested after 12 hours post-infection. Western blot analysis was used to evaluate the levels of IAV nucleoprotein (NP) and nucleolin (NCL) in whole cells and in the nuclear and cytoplasmic fractions. Histone H3 and GAPDH served as loading controls for nuclear and cytoplasmic fractionation. Western blot data for IAV NP protein levels in whole-cell, nuclear, and cytoplasmic fractions are presented as graphs. The levels of IAV NP in the IAV-infected, mock-treated control, IAV-infected, AGM-380d-treated group, and IAV-infected, AGM-380t-treated group were normalized to GAPDH for the whole cell and cytoplasmic fractions, and to histone H3 for the nuclear fraction. In each

graph, the reduction in NP protein level from treatment with AGM380d or AGM-380t was compared with the NP level in IAV-infected, mock-treated controls, and the relative reduction was shown. (C) Confocal images showing IAV NP in MDCK cells, with or without treatment with AGM-380d or AGM-380t. Treatment of infected cells with 10 μM of AGM-380d or AGM-380t resulted in the retention of IAV NP in the nucleus, compared to vehicle-treated controls. IAV NP (red) is a component of vRNP complexes, while NCL (green) facilitates their nuclear trafficking. The results in the graph are expressed as relative fluorescence intensity, calculated as the mean pixel intensity of positively stained areas in virus-infected groups normalized to the mock-treated, mock-infected control. Bar = 10 μm. (D, E) Graphs showing changes in relative viral genome copies (D) and titers (E) of IAV PR8 (H1N1) strain (MOI = 1) at 24 hours post-infection. Data are expressed as means ± standard deviation. *$P < 0.05$, **$P < 0.01$, ***$P < 0.001$, via one-way ANOVA with Tukey's correction.

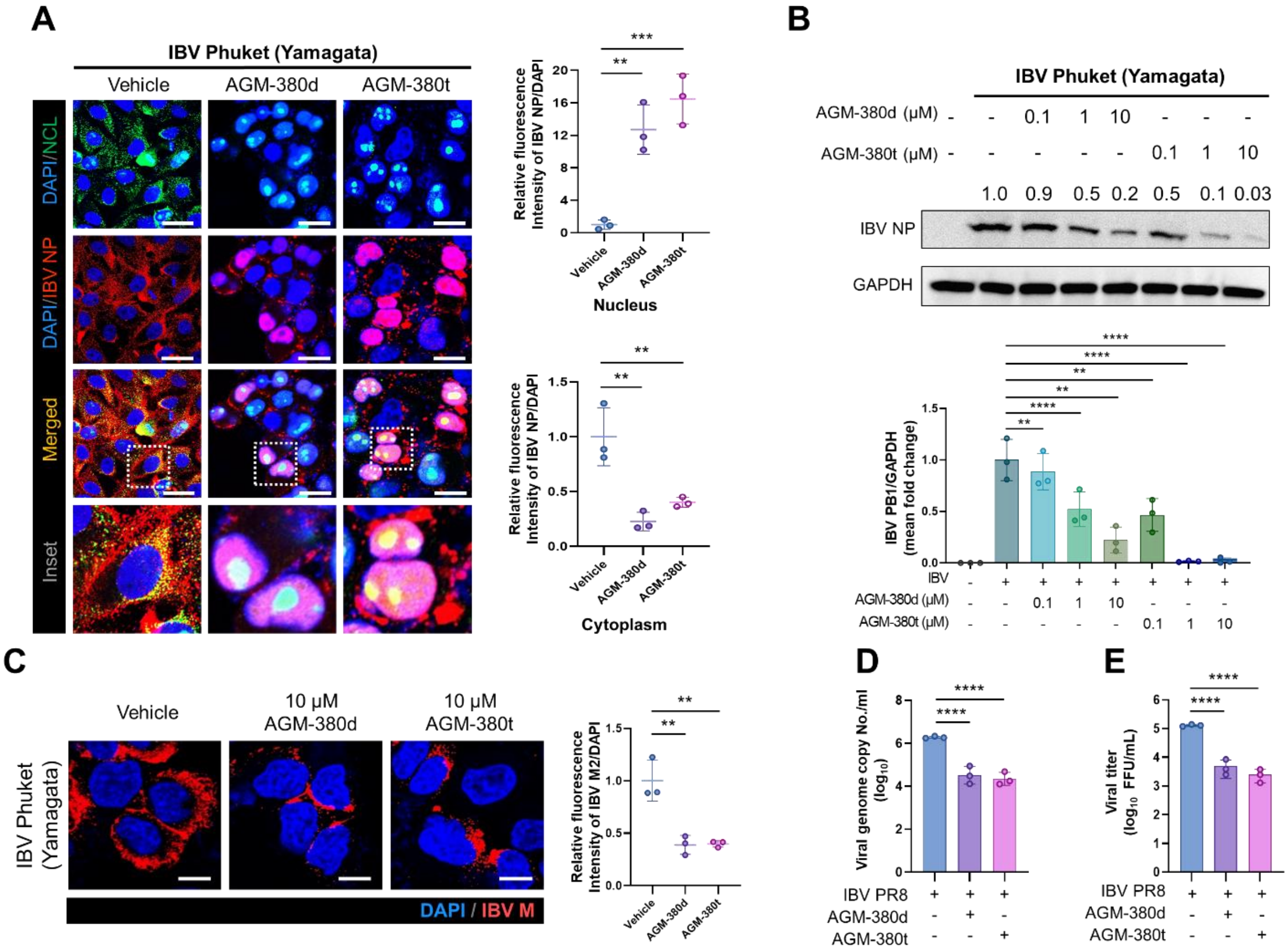

Fig. S9. Inhibition of nuclear trafficking of influenza B virus nucleoprotein (NP) by AGM-380d and AGM-380t. (A) Representative confocal image showing IBV NP in A549 cells, with or without treatment with AGM-380d or AGM-380t. Treatment of A549 cells infected with IBV Phuket/3073/2013 (Yamagata lineage) with AGM-380d or AGM-380t at a concentration of 10 µM resulted in retention of IBV NP in the nuclei compared to virus-infected, vehicle-treated controls. IBV NP (nucleoprotein), shown in red, is a viral component of IBV vRNP complexes, while NCL (nucleolin), shown in green, facilitates the nuclear trafficking of IBV vRNP complexes. The results in the graph are expressed as relative fluorescence intensity, calculated as the mean pixel intensity of positively stained areas in virus-infected groups normalized to the mock-treated, mock-infected control. Bar = 10 µm. (B, C) Representative western blot and confocal images illustrating the antiviral effects of AGM-380d and AGM-380t. A549 cells infected with IBV Phuket/3073/2013 (Yamagata) strain (MOI = 0.1) or mock-infected were treated with various concentrations of AGM-380d or AGM-380t as indicated. The inhibitory effects were assessed using antibodies specific for IBV NP in western blot (B) or matrix-2 ion channel protein (M2, shown in red) in immunofluorescence (C) assays. The

results in the graph are expressed as relative fluorescence intensity, calculated as the mean pixel intensity of positively stained areas in virus-infected groups normalized to the mock-treated, mock-infected control. Bar = 5 μm. (D, E) Graphical representations showing sequential changes in relative viral genome copies (D) and titers (E) of Phuket/3073/2013 (Yamagata) strain (MOI = 0.1) at 24 hours post-infection. Results in the graphs are presented as means ± standard deviation. *$P < 0.05$, **$P < 0.01$, ***$P < 0.001$, one-way ANOVA with Tukey's correction for multiple comparisons.

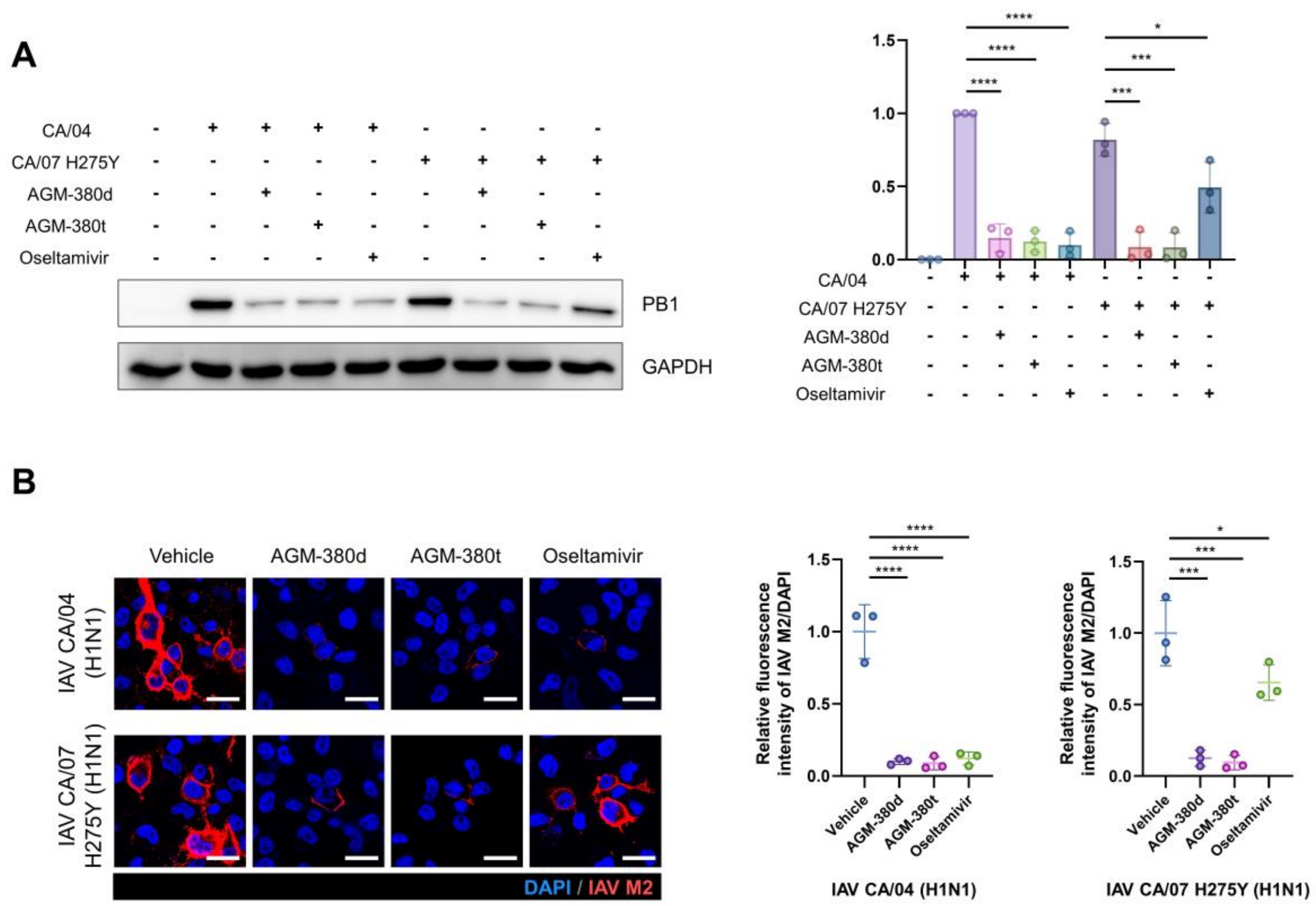

Fig. S10. Inhibition of oseltamivir-susceptible and -resistant influenza A virus by AGM-380d and AGM-380t. (A) Representative western blot images (left) and their graphical representation (right) illustrating the antiviral effects of AGM-380d, AGM-380t, or oseltamivir. A549 cells infected with either oseltamivir-susceptible A/California/04/2009 (H1N1) or oseltamivir-resistant A/California/07/2009 (H1N1) strains (MOI = 0.1) or mock-infected were treated with AGM-380d, AGM-380t, or oseltamivir carboxylate at a concentration of 10 µM. The inhibitory effects were assessed using an antibody specific for IAV NP. Western blot data for IAV NP protein levels are presented as graphs. The levels of IAV NP in the IAV-infected, mock-treated control, IAV-infected, AGM-380d-treated group, IAV-infected, AGM-380t-treated group, and IAV-infected, oseltamivir-treated group were normalized to GAPDH. The reduction in NP protein level from treatment with AGM380d, AGM-380t, or oseltamivir was compared with the NP level in IAV-infected, mock-treated controls, and the relative reduction was shown. (B) Representative confocal images (left) and their graphical representation (right) showing the antiviral effects of AGM-380d, AGM-380t, or oseltamivir. The experimental conditions are the same as the above western blot analysis. The results in the graph are

expressed as relative fluorescence intensity, calculated as the mean pixel intensity of positively stained areas in virus-infected groups normalized to the mock-treated, mock-infected control. Bar = 10 μm. Results in the graphs are presented as means ± standard deviation. *$P < 0.05$, **$P < 0.01$, ***$P < 0.001$, one-way ANOVA with Tukey's correction for multiple comparisons.

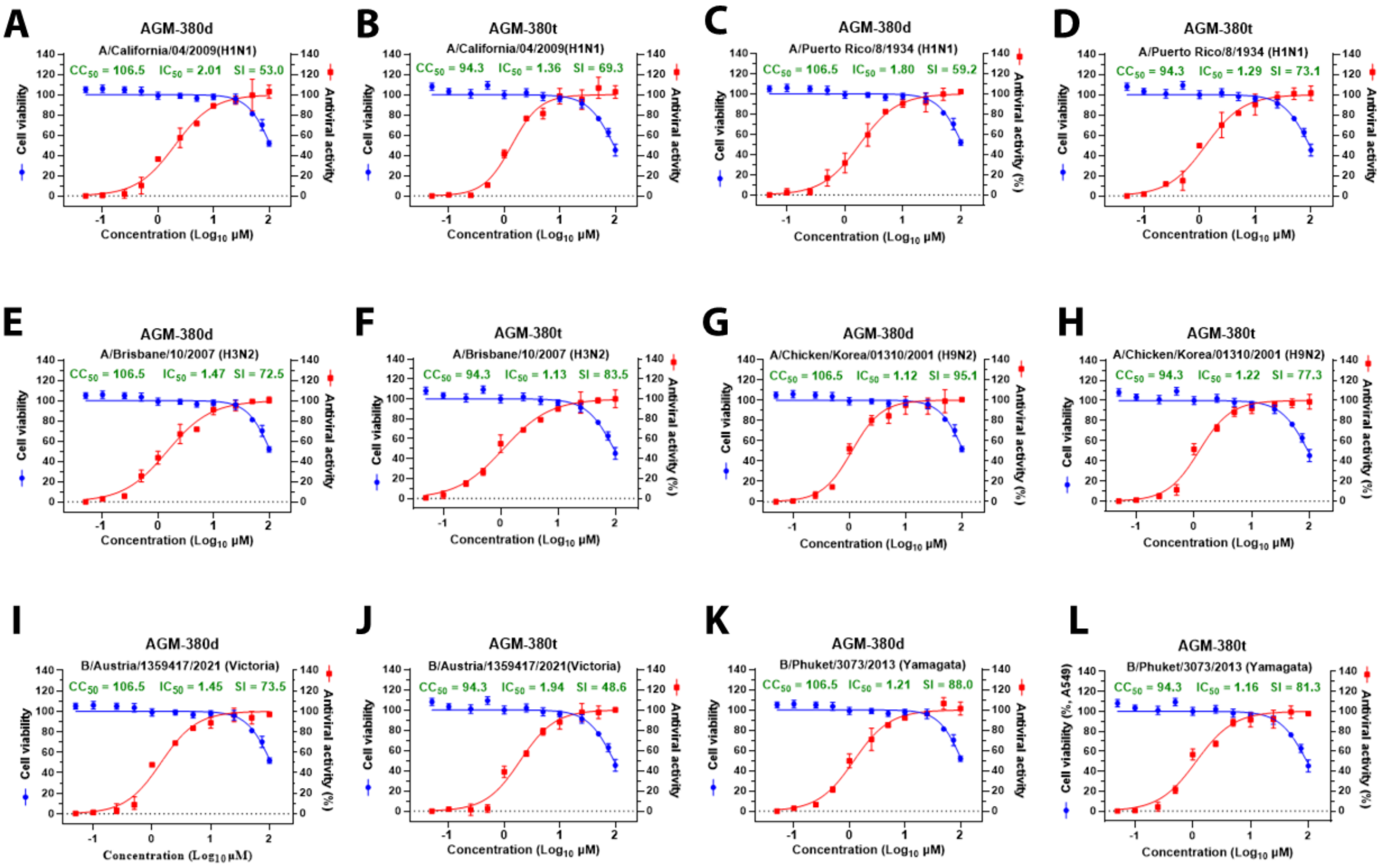

Fig. S11. *In vitro* broad-spectrum safety and antiviral effects of AGM-380d and AGM-380t against various strains of influenza A virus (IAV) and influenza B virus (IBV). (A-L) Dose-response curves showing the half-maximal inhibitory concentration (IC_{50}) and half-maximal cytotoxicity concentration (CC_{50}), along with the selective index (SI) of AGM-380d and AGM-380t against different IAV and IBV strains: A/California/04/2009 (H1N1) pandemic flu (A, B), A/Puerto Rico/8/1934 (H1N1) seasonal flu (C, D), A/Brisbane/10/2007 (H3N2) seasonal flu (E, F), A/Chicken/Korea/01310/2001 (H9N2) low pathogenic (G, H), B/Austria/1359417/2021 (Victoria lineage) seasonal flu (I, J), and B/Phuket/3073/2013 (Yamagata lineage) seasonal flu (K, L) strains. Viral yield in the cell supernatant was measured using a cell-culture immunofluorescence assay. Cytotoxicity of AGM-380d and AGM-380t to each cell line was assessed with an MTT assay. Data in the graphs are presented as the arithmetic mean ± standard deviation from three independent experiments.

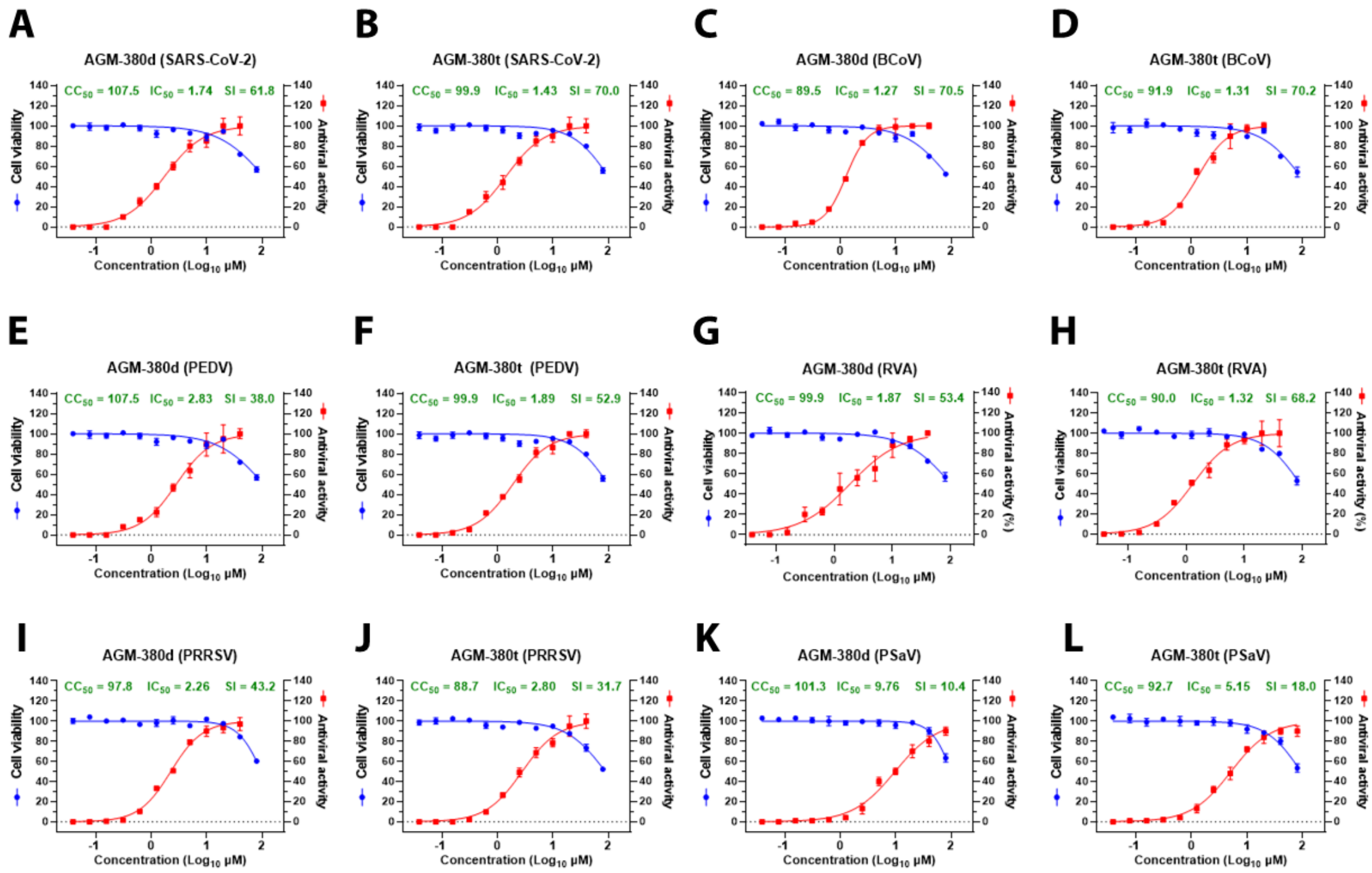

Fig. S12. *In vitro* broad-spectrum safety and antiviral activity of AGM-380d and AGM-380t against various RNA viruses. (A-L) Dose-response curves showing the half-maximal inhibitory concentration (IC_{50}), half-maximal cytotoxicity concentration (CC_{50}), and selectivity index (SI) of AGM-380d and AGM-380t against six different viruses: severe acute respiratory coronavirus 2 (SARS-CoV-2) (A, B), KCDC03; bovine coronavirus (BCoV) (C, D), KWD20; porcine epidemic diarrhea coronavirus (PEDV) (E, F), QIAP1401; bovine species A rotavirus (RVA) (G, H), NCDV; porcine reproductive and respiratory syndrome virus (PRRSV) (I, J), LMY; and porcine sapovirus (PSaV) (K, L), Cowden strains. Viral yield in cell supernatants was measured using a cell-culture immunofluorescence assay. The cytotoxicity of AGM-380d and AGM-380t to each cell line was assessed with an MTT assay. Data are shown as mean ± standard deviation from three independent experiments.

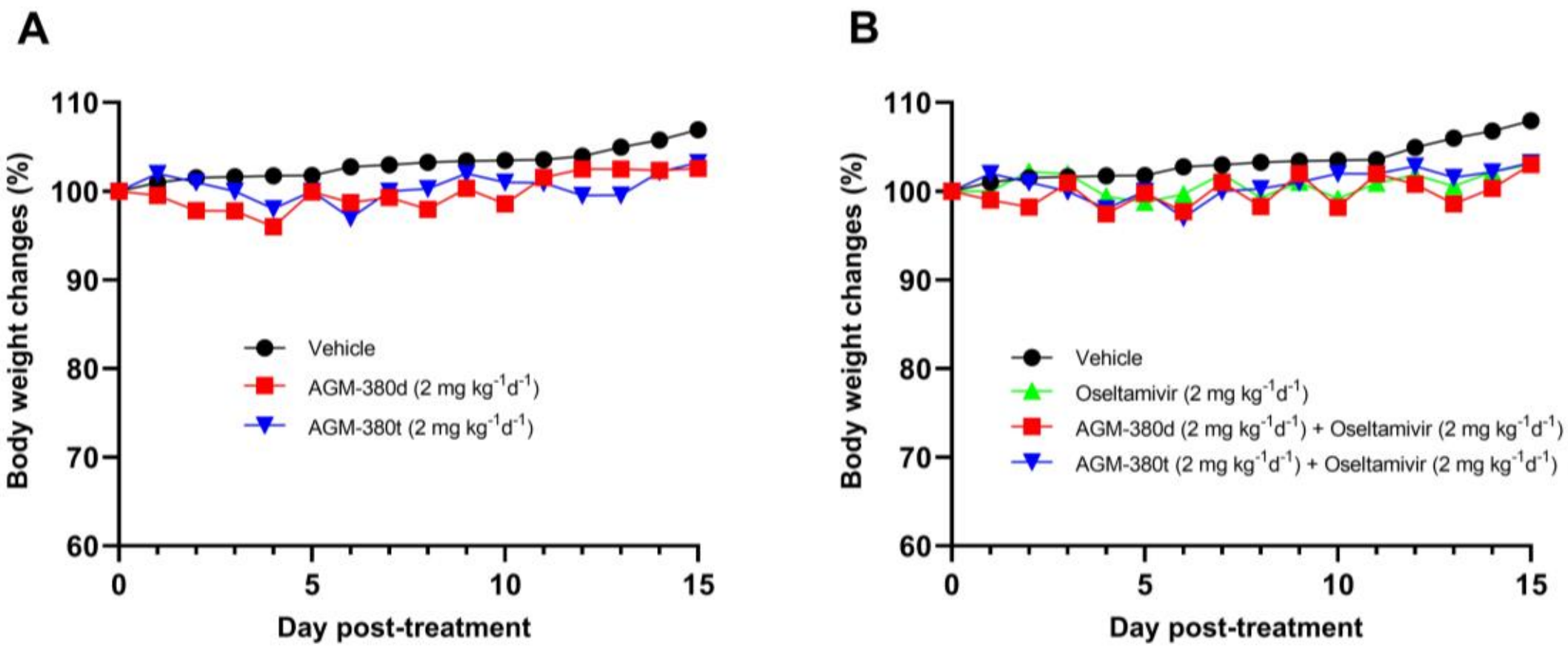

Fig. S13. *In vivo* toxicity of AGM-380d and AGM-380t in mice. (A) Sixteen mice per group received intraperitoneal injections of either 2 mg kg^{-1} d^{-1} AGM-380d, 2 mg kg^{-1} d^{-1} AGM-380t, or vehicle [10% PEG400 in water (vol/vol)] twice daily for four consecutive days; body weight was measured daily for 15 days. (B) Sixteen mice per group were injected intraperitoneally with either 2 mg kg^{-1} d^{-1} AGM-380d, 2 mg kg^{-1} d^{-1} AGM-380t, 2 mg kg^{-1} d^{-1} oseltamivir, a combination of 2 mg kg^{-1} d^{-1} AGM-380d or AGM-380t with 2 mg kg^{-1} d^{-1} oseltamivir, or vehicle [10% PEG400 in water (vol/vol)] for 15 days. The graph illustrates the changes in body weight for each group over the 15 days. Results are expressed as the mean daily body weight ± standard deviation.

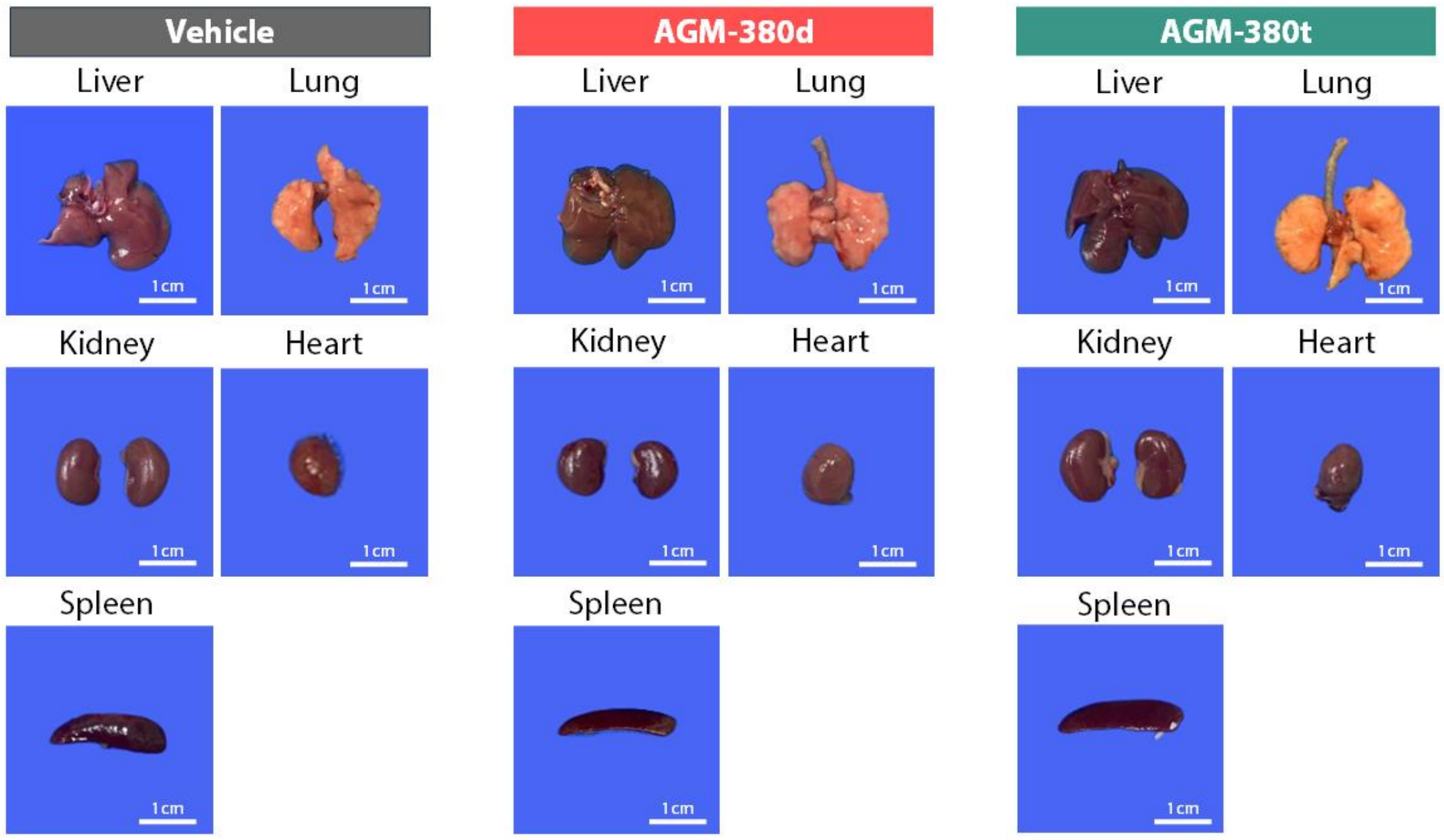

Fig. S14. *In vivo* organ-specific toxicity of AGM-380d and AGM-380t. Representative images showing gross findings of the liver, lung, kidney, heart, and spleen in mice treated with 2 mg kg^{-1} d^{-1} of AGM-380d or AGM-380t, or with vehicle. For the toxicological assessment of AGM-380d and AGM-380t in wild-type C57BL/6J mice, six groups of three anesthetized mice each were treated intraperitoneally with either AGM-380d or AGM-380t in 50 µL of vehicle [10% PEG400 in water (vol/vol)] at a dose of 2.0 mg kg^{-1} d^{-1}, twice daily (Bid), with a 12-hour interval for 4 consecutive days. One control group of three anesthetized mice received intraperitoneal injections of 50 µL of vehicle Bid at 12-hour intervals for 4 days. Scale bars = 1 cm.

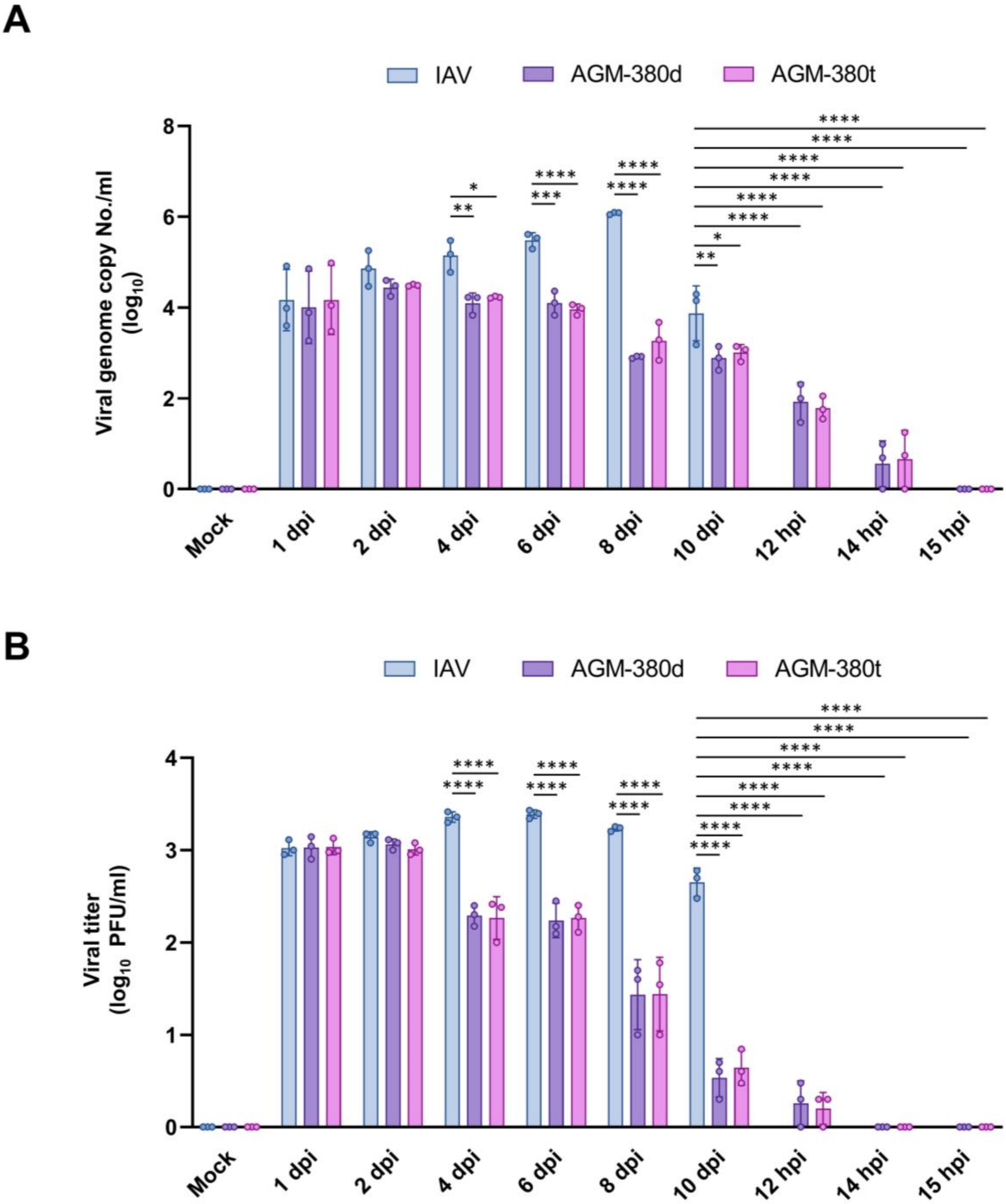

Fig. S15. *In vivo* antiviral effects of AGM-380d and AGM-380t against influenza A virus (IAV). (A) Graphical representation showing changes in viral genome copy number in lungs sampled from 1 day post-infection (dpi) to 15 dpi at 2-day intervals (from 2 dpi) from IAV-challenged mice (n = 3) with 10^3 PFU of the mouse-adapted IAV PR8 strain and treated with AGM-380d or AGM-380t. Viral genome copy number determined by RT-qPCR is normalized

to GAPDH, and changes in the treated groups are compared with those in the mock-treated control. (B) Graphical representation showing changes in viral titers in lungs sampled from mice as described above. Viral titers determined by a plaque-forming assay showed relative changes in the infected groups compared with mock-infected controls. Since all IAV-challenged, mock-treated mice died at 10 dpi, the relative viral genome copy number in the treated groups from 12 dpi to 15 dpi is compared with that sampled at 10 dpi in the mock-treated control. Results are expressed as means ± standard deviation. *$P < 0.05$, **$P < 0.01$, ***$P < 0.001$, and ****$P < 0.0001$; one-way ANOVA with Tukey's correction for multiple comparisons.

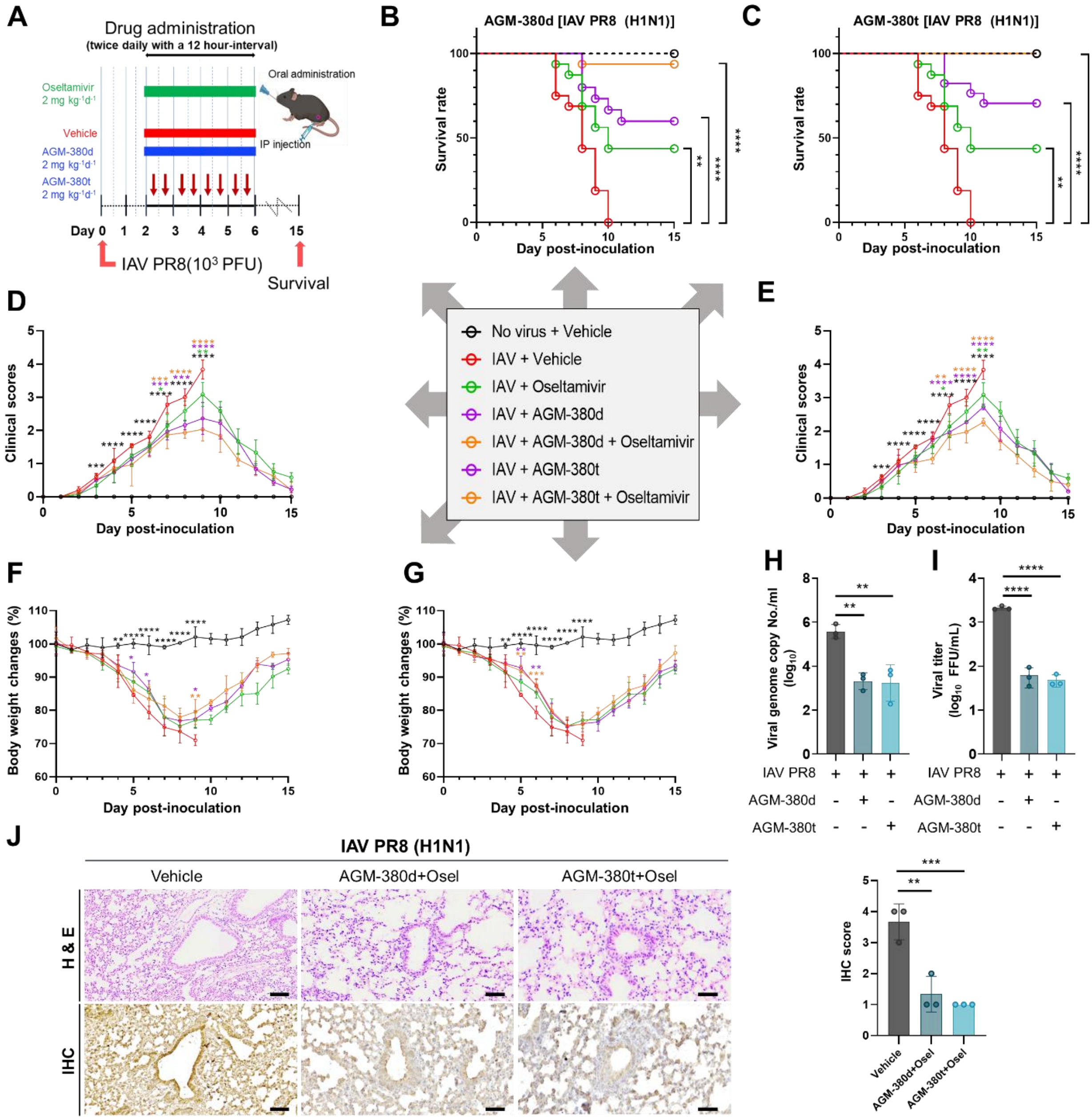

Fig. S16. Synergistic *in vivo* antiviral effects of combination therapy. (A) The administration scheme involved giving mice (n = 16) either 2 mg kg^{-1} d^{-1} of AGM-380d, 2 mg kg^{-1} d^{-1} of AGM-380t, or 2 mg kg^{-1} d^{-1} of oseltamivir. These treatments were administered individually or in combination, twice daily, for four consecutive days, starting two days after the mice were challenged with 10^3 PFU of the mouse-adapted influenza A virus (IAV) PR8 strain. (B, C) Survival rates (shown as percentages) of IAV-challenged mice after treatment with AGM-380d (B) or AGM-380t (C), either alone or combined with oseltamivir. (D, E) Effect of treatments with AGM-380d (D) or AGM-380t (E), alone or with oseltamivir, on reducing

clinical scores. (F, G) Effect of treatments with AGM-380d (F) or AGM-380t (G), individually or in combination with oseltamivir, on body weight recovery. (H, I) Graphical representation showing changes in viral genome copy number (H) and titers (I) at various time points after challenging mice (n = 3) with 10^3 PFU of the mouse-adapted IAV PR8 strain and treated with AGM-380d or AGM-380t combined with oseltamivir. Viral genome copy number determined by RT-qPCR was normalized to GAPDH, and relative changes in the treated groups were compared with those in the mock-treated control. Viral titers determined by a plaque-forming assay showed relative changes in the infected groups compared with mock-infected controls. Representative images showing histological lesions and antigen distribution in the lung. Amelioration of histological lung lesions (upper panels) and inhibition of IAV replication (lower panels) observed with AGM-380d or AGM-380t. The results in the graph (right) are expressed as IHC scores, calculated as the mean pixel intensity of positively stained areas in virus-infected groups normalized to the mock-treated, mock-infected control. Scale bar = 200 μm. Results are shown as means ± standard deviation. *P < 0.05; **P < 0.01; ***P < 0.001, using one-way ANOVA

Supplementary Tables

Table S1. The median cytotoxic concentration (CC_{50}) of AGM-380d and AGM-380t in different cell lines determined by measuring the cellular NAD(P)H-dependent cellular oxidoreductase enzymes (MTT assay)

Cell line	CC_{50}	
	**AGM-380d**	**AGM-380t**
MDCK	106.5 ± 2.0	94.3 ± 2.0
Vero E6	107.5 ± 2.0	99.9 ± 2.0
HRT-18G	89.5 ± 1.9	91.9 ± 1.9
MA104	99.9 ± 2.0	90.0 ± 1.9
MARC-145	97.8 ± 2.0	88.7 ± 1.9
LLC-PK	101.3 ± 2.0	92.7 ± 1.9

Table S2. The half maximal (50%) inhibitory concentration (IC_{50}) and selectivity index (SI) of AGM-380d and AGM-380t for RNA viruses within eight virus genera

Virus [a]	Strain	AGM-380d		AGM-380t	
		IC_{50} (µM)	SI (CC_{50}/IC_{50})	IC_{50} (µM)	SI (CC_{50}/IC_{50})
IAV	California/04/2009 (H1N1)	2.01 ± 0.30	53.0	1.36 ± 0.13	69.3
	Puerto Rico/8/1934 (H1N1)	1.80 ± 0.25	59.2	1.29 ± 0.11	73.1
	Brisbane/10/07 (H3N2)	1.47 ± 0.17	72.5	1.13 ± 0.05	83.5
	Chicken/Korea/01310/2001 (H9N2)	1.12 ± 0.05	95.1	1.22 ± 0.09	77.3
IBV	Austria/1359417/2021 (B/Victoria)	1.45 ± 0.16	73.5	1.94 ± 0.29	48.6
	Phuket/3073/2013 (B/Yamagata)	1.21 ± 0.08	88.8	1.16 ± 0.06	81.3
SARS-CoV-2	KCDC03	1.74 ± 0.24	61.8	1.43 ± 0.15	70.0
BCoV	KWD20	1.27 ± 0.10	70.5	1.31 ± 0.12	70.2
PEDV	QIAP1401	2.83 ± 0.45	38.0	1.89 ± 0.28	52.8
RVA	NCDV	1.89 ± 0.28	53.0	1.32 ± 0.12	68.2
PRRSV	LMY	2.26 ± 0.35	43.2	2.80 ± 0.45	31.7
PSaV	Cowden	9.76 ± 0.98	10.4	5.15 ± 0.71	18.0

[a]IAV, influenza A virus; IBV, influenza B virus; SARS-CoV-2, severe acute respiratory syndrome coronavirus 2; BCoV, bovine coronavirus; PEDV, porcine epidemic diarrhea coronavirus; RVA, species A rotavirus; PRRSV, porcine reproductive and respiratory syndrome virus.

Table S3. Cell culture information

Cell line	Origin	Medium	Supplementation	Application	Source	Catalog number
A549	Human lung carcinoma	DMEM	10% FBS, 1% P/S	IAV and IBV culture	ATCC	CRM-CCL-185
MDCK	Canine kidney epithelium	EMEM	10% FBS, 1% P/S	IAV and IBV culture	ATCC	CRL-2935
MDBK	Bovine kidney epithelium	EMEM	10% FBS, 1% P/S	IAV and IBV culture	ATCC	CCL-22
Calu-3	Human lung adenocarcinoma	EMEM	10% FBS, 1% P/S	IAV and IBV culture	ATCC	HTB-55
Caco-2	Human colorectal adenocarcinoma	EMEM	10% FBS, 1% P/S	IAV and IBV culture	ATCC	HTB-37
Vero E6	African green monkey kidney epithelium	EMEM	10% FBS, 1% P/S	PEDV and SARS-CoV-2 culture	ATCC	CRL-1586
HRT-18G	Human colorectal adenocarcinoma	DMEM	10% FBS, 1% P/S	BCoV culture	ATCC	CRL-3609
MA104	Rhesus monkey kidney epithelium	α-MEM	10% FBS, 1% P/S	RVA culture	ATCC	CRL-2378.1

MARC-145	African green monkey kidney epithelium	DMEM	10% FBS, 1% P/S	PRRSV culture	ATCC	CRL-12230
LLC-PK	Porcine kidney epithelium	EMEM	10% FBS, 1% P/S	PSaV culture	ATCC	CL-101

Abbreviations: ATCC, American Type Culture Collection; FBS, fetal bovine serum; DMEM, Dulbecco's modified Eagle's medium; EMEM, Eagle's Minimum Essential Medium; α-MEM, Minimal Essential Medium alpha modification; 1% P/S, 100 U/mL penicillin and 100 μg/mL streptomycin; IAV, influenza A virus; IBV, influenza B virus; PEDV, porcine epidemic diarrhea virus; SARS-CoV-2, severe acute respiratory syndrome virus 2; BCoV, bovine coronavirus; RVA, species A rotavirus; PRRSV, porcine reproductive and respiratory syndrome virus; PSaV, porcine sapovirus.

Table S4. Virus information

Viruses	Strain name (genotypes)	Source
Influenza A virus (IAV)	Puerto Rico/8 (PR8) (H1N1)	American Type Culture Collection
	California/04/2009 (H1N1)	GC Biopharma Hwasun Plant
	California/07/2009 (H1N1)	Korea Disease Control and Prevention Agency
	Brisbane/10/07 (H3N2)	GC Biopharma Hwasun Plant
	Chicken/Korea/01310/2001 (H9N2)	Green Cross Veterinary Products
Influenza B virus (IBV)	Austria/1359417/2021 (B/Victoria)	GC Biopharma Hwasun Plant
	Phuket/3073/2013 (B/Yamagata)	GC Biopharma Hwasun Plant
Severe Acute Respiratory Syndrome coronavirus 2 (SARS-CoV-2)	KCDC03 (Lineage A)	Korea Disease Control and Prevention Agency
Bovine coronavirus (BCoV)	KWD20	Isolated from fecal samples and propagated in HRT-18G cells
Porcine epidemic diarrhea coronavirus (PEDV)	QIAP1401 (G2b)	Animal and Plant Quarantine Agency, Korea
Bovine species A rotavirus (bovine RVA)	NCDV (G6P6[1])	American Type Culture Collection
Porcine Reproductive and Respiratory Syndrome virus (PRRSV)	LMY (North American type)	Animal and Plant Quarantine Agency, Korea

Porcine sapovirus (PSaV)	Cowden (GIII.1)	A kind gift from Dr. K.O. Chang, Kansas State University

Table S5. Virus culture information

Viruses	Cell lines	Activation of viruses	Medium	Supplementation*
IAV	A549	N/A	DMEM	1 μg/mL TPCK-treated trypsin, 1% P/S
	MDCK	N/A	DMEM	1 μg/mL TPCK-treated trypsin, 1% P/S
IBV	A549	N/A	DMEM	1 μg/mL TPCK-treated trypsin, 1% P/S
	MDCK	N/A	DMEM	1 μg/mL TPCK-treated trypsin, 1% P/S
SARS-CoV-2	Vero E6	N/A	EMEM	1 μg/mL TPCK-treated trypsin, 1% P/S
BCoV	HRT-18G	N/A	DMEM	5 μg/mL porcine pancreatin, 1% P/S
PEDV	Vero E6	N/A	EMEM	3 μg/mL porcine pancreatic trypsin, 1% P/S
Bovine RVA	MA104	Preactivation with 10 μg/mL porcine trypsin	α-MEM	1 μg/mL crystalized trypsin, 1% P/S
PRRSV	MARC-145	N/A	DMEM	1% P/S
PSaV	LLC-PK	N/A	EMEM	2.5% FBS, 200 μM GCDCA, 1% P/S

Abbreviations: FBS, fetal bovine serum; DMEM, Dulbecco's modified Eagle's medium; EMEM, Eagle's Minimum Essential Medium; α-MEM, alpha Minimal Essential Medium; 1% P/S, 100 U/mL penicillin, 100 μg/mL streptomycin; TPCK-treated trypsin, N-tosyl-L-phenylalanine chloromethyl ketone (TPCK)-treated trypsin; GCDCA, glycochenodeoxycholic acid.

Table S6. Chemicals, kits and siRNAs used in this study

Regents	Company/Catalogue number	City/State	Country	Solvent	Application*
**Chemicals**					
Porcine pancreatic trypsin	Sigma Aldrich/P3292	St. Louis, MO	USA	PBS	PEDV culture
Crystalized trypsin	Gibco/27250-018	Fort Worth, TX	USA	PBS	Bovine RVA
Porcine pancreatin	Gibco/J62126.30	Fort Worth, TX	USA	PBS	BCoV culture
TPCK-treated trypsin	Sigma Aldrich/T1426	St. Louis, MO	USA	PBS	IAV, IBV, and SARS-CoV-2 culture
GCDCA	Sigma Aldrich/G0759	St. Louis, MO	USA	PBS	PSaV culture
MTT	Sigma Aldrich/475989	St. Louis, MO	USA	PBS	Cytotoxicity assay
Triton X-100	Sigma Aldrich/X165	St. Louis, MO	USA	PBS	IFA
SlowFade Gold Antifade Mountant	Molecular Probes/S36936	Eugene, OR	USA	-	IFA
Ultrapure agarose	Thermo Scientific/16500500	Waltham, MA	USA	DDW	Western blotting
Paraformaldehyde	Sigma Aldrich/158127	St. Louis, MO	USA	-	IFA
Oseltamivir	Sigma Aldrich/SML1606	St. Louis, MO	USA	PBS	Anti-IAV and -IBV assay
**Kits**					
Bicinchoninic acid (BCA) protein assay	Thermo Scientific/A55864	Waltham, MA	USA	-	Western blotting
NE-PER™ Nuclear and Cytoplasmic Extraction	Thermo Scientific/78833	Waltham, MA	USA	-	Western blotting

Reagents					
RNeasy mini kit	Qiagen/74104	Hilden	Germany	-	RT-qPCR
TOPscript™ cDNA synthesis kit	Enzynomics/EZ005S	Daejeon	South Korea	-	RT-qPCR
Duolink PLA kit	Sigma-Aldrich/DUO92008	St. Louis, MO	USA		In situ interaction between NCL and NPs of IAV and IBV
**siRNAs**					
AccuTarget™ Predesigned siRNA (mixtures)	BIONEER/K-7920	Daejeon	Korea	RNAse free water	Knockdown of NCL
Lipofectamine3000	Thermo Scientific/L3000001	Waltham, MA	USA	-	Transfection of siRNAs

*PEDV, porcine epidemic diarrhea virus; RVA, species A rotavirus; BCoV, bovine coronavirus; IAV, influenza A virus; IBV, influenza B virus; SARS-CoV-2, severe acute respiratory coronavirus 2; PSaV, porcine sapovirus; IFA, immunofluorescence assay.

Table S7. Antibodies used in this study

Antibodies*	Host	Type*	Conju-gation	Company/ Catalogue No.	City/State	Country	Solvent*	Application**
**Primary antibodies**								
SARS-CoV-2 S	Rabbit	Pab	-	Prosci/3525	San Diego, CA	USA	TBST (5% BSA)	Western blotting, IFA, CCIF
SARS-CoV-2 N	Mouse	Mab	-	Prosci/10078	San Diego, CA	USA	TBST (5% BSA)	Western blotting, IFA, CCIF
IAV M2	Mouse	Mab	-	Abcam/ab5416	Cambridge	UK	TBST (5% BSA)	Western blotting, IFA
IAV NP	Mouse	Mab	-	Abcam/ab128193	Cambridge	UK	TBST (5% BSA)	Western blotting, IFA
IAV PB1	Rabbit	Pab	-	Novus Biologicals/NBP2-42877	Centennial, CO	USA		Western blotting, IFA, CCIF
Histone H3	Rabbit	Pab	-	Abcam/ab1791	Beverly, MA	USA	TBST (5% BSA)	Western blotting,
IAV virion	Goat	Pab	-	Viasat/1304 (JF024)	Carlsbad, CA	USA	TBST (5% BSA)	IHC
IBV NP	Mouse	Mab		Thermo Scientific/MA5-35900	Waltham, MA	USA	TBST (5% BSA)	Western blotting, IFA, CCIF
IBV M	Mouse	Pab		Thermo Scientific/PA5-111968	Waltham, MA	USA	TBST (5% BSA)	Western blotting
GAPDH	Mouse	Mab	-	Santa Cruz/sc-47724	Dallas, TX,	USA	TBST (5% BSA)	Western blotting
β-actin	Mouse	Mab	-	Thermo Scientific/MA1-140	Waltham, MA	USA	TBST (5% BSA)	Western blotting

NCL	Rabbit	Pab	-	Cell Signaling/D4C7O -14574S	Beverly, MA	USA	TBST (5% BSA)	Western blotting, IFA
BCoV S	Mouse	Mab	-	Native Antigen Company/ MAB12430-100	Killington	UK	TBST (5% BSA)	Western blotting, IFA, CCIF
PEDV N	Mouse	Mab	-	Medgene Labs/9191	Brookings, SD	USA	TBST (5% BSA)	Western blotting, IFA, CCIF
RVA VP6	Mouse	Mab	-	Median Diagnostic/9201	Chuncheon	Korea	TBST (5% BSA)	
PRRSV M	Rabbit	Pab	-	Bioss Antibodies/bs-4507R	Woburn, MA	USA	TBST (5% BSA)	Western blotting, IFA, CCIF
PSaV VPg	Rabbit	Anti-serum	-	Manufactured in the lab[a]			TBST (5% BSA)	Western blotting, IFA, CCIF
**Secondary antibodies**								
Rabbit IgG (H+L)	Goat	Pab	HRP	Cell Signaling/707 45	Beverly, MA	USA	1xTBST	Western blotting, IHC
Goat IgG (H+L)	Rabbit	Pab	HRP	Santa Cruz/sc-2020	Beverly, MA	USA	1xTBST	Western blotting
Mouse IgGκ	Goat	Mab	HRP	Santa Cruz/sc-516 102	Dallas, TX	USA	1xTBST	Western blotting, IHC
AF647 rabbit IgG	Donkey	Pab	AF647	Thermo Scientific/ A21244	Waltham, MA	USA	1xPBS	IFA, CCIF
AF594 rabbit IgG	Donkey	Pab	AF594	Thermo Scientific/A21207	Waltham, MA	USA	1xPBS	IFA, CCIF
AF488 rabbit IgG	Donkey	Pab	AF488	Thermo Scientific/A11008	Waltham, MA	USA	1xPBS	IFA, CCIF
AF647 mouse IgG	Goat	Pab	AF647	Thermo Scientific/A21235	Waltham, MA	USA	1xPBS	IFA, CCIF
AF594 mouse IgG	Goat	Pab	AF594	Thermo Scientific/A21203	Waltham, MA	USA	1xPBS	IFA, CCIF

AF488 mouse IgG	Goat	Pab	AF488	Thermo Scientific/A10680	Waltham, MA	USA	1xPBS	IFA, CCIF
AF488 Phalloidin			AF488	Thermo Scientific/A12379	Waltham, MA	USA	1xPBS	IFA
AF594 Phalloidin			AF594	Thermo Scientific/ab176757	Waltham, MA	USA	1xPBS	IFA
AF647 Phalloidin			AF647	Thermo Scientific/ab176759	Waltham, MA	USA	1xPBS	IFA

*Abbreviation: SARS-CoV-2 S, SARS-CoV-2 spike; SARS-CoV-2 N, SARS-CoV-2 nucleocapsid; IAV M2, IAV matrix protein 2; IAV NP, IAV nucleoprotein; IBV NP, IBV nucleoprotein; IBV M, IBV membrane; NCL, nucleolin; BCoV S, BCoV spike protein; PEDV N, PEDV nucleoprotein; RVA VP6, RVA viral structural protein 6; PRRSV M, PRRSV matrix protein; PSaV VPg, PSaV viral genome-linked protein; Mab or Pab, monoclonal or polyclonal antibodies; HRP, horseradish peroxidase; 1xTBST, 1x Tris-Buffered Saline containing 0.1% Tween® 20 Detergent; TBST (5% BSA), 1x TBST containing 5% bovine serum albumin.

[a]Generated by immunization of a New Zealand White rabbit with purified PSaV VPg (2).

**IFA, immunofluorescence assay; CCIF, cell culture immunofluorescence assay; IHC, immunohistochemistry.

Table S8. Experimental design for determining organ-specific toxicity of AGM-380d and AGM-380t in the mice

Group	Animal			Treatment				Exp. Period
	Breed	Age	No.	Chemical	Dosage	Route	Administration	(day)
1	C57BL/6J	8 wk	3	Vehicle[a]	None	IP[b]	Bid[c] for 4 days	5
2	C57BL/6J	8 wk	3	AGM-380d	2.0 mg kg^{-1} d^{-1}	IP	Bid for 4 days	5
3	C57BL/6J	8 wk	3	AGM-380t	2.0 mg kg^{-1} d^{-1}	IP	Bid for 4 days	5

[a]Vehicles for mice, 10% PEG400 in water (v/v), respectively.

[b]IP, intraperitoneal administration.

[c]Bid, twice daily with a 12-hour interval.

Table S9. Experimental design for determining antiviral effects of AGM-380d on influenza A virus infection in the mouse model

Group	Animal			Inoculum			Treatment				Ex. Period (day)
	Breed	Age	No.	Strain	Titer[a]	Route[b]	Chemical	Dosage	Route	No. of doses	
1	C57BL/6J	8 wk	16	PBS	None	IN	Vehicle[c]	None	IP[d]	Bid[e] for 4 days	15
2	C57BL/6J	8 wk	16	PR8	10^3 PFU	IN	Vehicle	None	IP	Bid for 4 days	15
3	C57BL/6J	8 wk	16	PR8	10^3 PFU	IN	AGM-380d	0.5 mg kg^{-1} d^{-1}	IP	Bid for 4 days	15
4	C57BL/6J	8 wk	16	PR8	10^3 PFU	IN	AGM-380d	1.0 mg kg^{-1} d^{-1}	IP	Bid for 4 days	15
5	C57BL/6J	8 wk	16	PR8	10^3 PFU	IN	AGM-380d	2.0 mg kg^{-1} d^{-1}	IP	Bid for 4 days	15

[a]PFU, plaque-forming unit.

[b]IN, intranasal inoculation.

[c]Vehicle, 10% PEG400 in water (v/v).

[d]IP, intraperitoneal administration.

[e]Bid, twice daily with a 12-hour interval.

Table S10. Experimental design for determining antiviral effects of AGM-380t on influenza A virus infection in the mouse model

Group	Animal			Inoculum			Treatment				Ex. Period (day)
	Breed	Age	No.	Strain	Titer[a]	Route[b]	Chemical	Dosage	Route	No. of doses	
1	C57BL/6J	8 wk	16	PBS	None	IN	Vehicle[c]	None	IP[d]	Bid[e] for 4 days	15
2	C57BL/6J	8 wk	16	PR8	10^3 PFU	IN	Vehicle	None	IP	Bid for 4 days	15
3	C57BL/6J	8 wk	16	PR8	10^3 PFU	IN	AGM-380t	0.5 mg kg^{-1} d^{-1}	IP	Bid for 4 days	15
4	C57BL/6J	8 wk	16	PR8	10^3 PFU	IN	AGM-380t	1.0 mg kg^{-1} d^{-1}	IP	Bid for 4 days	15
5	C57BL/6J	8 wk	16	PR8	10^3 PFU	IN	AGM-380t	2.0 mg kg^{-1} d^{-1}	IP	Bid for 4 days	15

[a]PFU, plaque-forming unit.

[b]IN, intranasal inoculation.

[c]Vehicle, 10% PEG400 in water (v/v).

[d]IP, intraperitoneal administration.

[e]Bid, twice daily with a 12-hour interval.

Table S11. Experimental design for determining the combination therapy of AGM-380d and oseltamivir on influenza A virus infection in the mouse model

Group	Animal			Inoculum			Treatment				Ex. Period (day)
	Breed	Age	No.	Strain	Titer[a]	Route[b]	Chemical	Dosage	Route	No. of doses	
1	C57BL/6J	8 wk	16	PBS	None	IN	Vehicle[c]	None	IP[d]	Bid[e] for 4 days	15
2	C57BL/6J	8 wk	16	PR8	10^3 PFU	IN	Vehicle	None	IP	Bid for 4 days	15
3	C57BL/6J	8 wk	16	PR8	10^3 PFU	IN	Oseltamivir	2.0 mg kg^{-1} d^{-1}	Oral	Bid for 4 days	15
4	C57BL/6J	8 wk	16	PR8	10^3 PFU	IN	AGM-380d	2.0 mg kg^{-1} d^{-1}	IP	Bid for 4 days	15
5	C57BL/6J	8 wk	16	PR8	10^3 PFU	IN	AGM-380d<br>Oseltamivir	2.0 mg kg^{-1} d^{-1}<br>2.0 mg kg^{-1} d^{-1}	IP<br>Oral	Bid for 4 days	15

[a]PFU, plaque-forming unit.

[b]IN, intranasal inoculation.

[c]Vehicle, 10% PEG400 in water (v/v).

[d]IP, intraperitoneal administration.

[e]Bid, twice daily with a 12-hour interval.

Table S12. Experimental design for determining the combination therapy of AGM-380t and oseltamivir on influenza A virus infection in the mouse model

Group	Animal			Inoculum			Treatment				Ex. Period (day)
	Breed	Age	No.	Strain	Titer[a]	Route[b]	Chemical	Dosage	Route	No. of doses	
1	C57BL/6J	8 wk	16	PBS	None	IN	Vehicle[c]	None	IP[d]	Bid[e] for 4 days	15
2	C57BL/6J	8 wk	16	PR8	10^3 PFU	IN	Vehicle	None	IP	Bid for 4 days	15
3	C57BL/6J	8 wk	16	PR8	10^3 PFU	IN	Oseltamivir	2.0 mg kg^{-1} d^{-1}	Oral	Bid for 4 days	15
4	C57BL/6J	8 wk	16	PR8	10^3 PFU	IN	AGM-380t	2.0 mg kg^{-1} d^{-1}	IP	Bid for 4 days	15
5	C57BL/6J	8 wk	16	PR8	10^3 PFU	IN	AGM-380t<br>Oseltamivir	2.0 mg kg^{-1} d^{-1}<br>2.0 mg kg^{-1} d^{-1}	IP<br>Oral	Bid for 4 days	15

[a]PFU, plaque-forming unit.

[b]IN, intranasal inoculation.

[c]Vehicle, 10% PEG400 in water (v/v).

[d]IP, intraperitoneal administration.

[e]Bid, twice daily with a 12-hour interval.

Table S13. Experimental design for determining inhibitory effects of AGM-380d and AGM-380t on virus replication and histopathological lesions in the lungs from IAV-infected mice

Group	Animal			Inoculum			Treatment				Ex.
	Breed	Age	No.	Strain	Titer[a]	Route	Chemical	Dosage	Route	No. of doses	Period (day)
1	C57BL/6J	8 wk	4	PBS	None	IN[a]	Vehicle[c]	None	IP[d]	Bid[e] for 4 days	6
2	C57BL/6J	8 wk	4	PR8	10^3 PFU	IN	Vehicle	None	IP	Bid for 4 days	6
3	C57BL/6J	8 wk	4	PR8	10^3 PFU	IN	AGM-380d	2.0 mg kg^{-1} d^{-1}	IP	Bid for 4 days	6
4	C57BL/6J	8 wk	4	PR8	10^3 PFU	IN	AGM-380t	2.0 mg kg^{-1} d^{-1}	IP	Bid for 4 days	6

[a]PFU, plaque-forming unit.

[b]IN, intranasal inoculation.

[c]Vehicle, 10% PEG400 in water (v/v).

[d]IP, intraperitoneal administration.

[e]Bid, twice daily with a 12-hour interval.

Table S14. Experimental design for determining the dynamics of host nucleolin in the lungs from IAV-infected mice

Group	Animal			Inoculum			Ex. Period
	Breed	Age	No.	Strain	Titer[a]	Route[b]	(day)
1	C57BL/6J	8 wk	4	PBS	None	IN	4
2	C57BL/6J	8 wk	4	PR8	10^3 PFU	IN	2
3	C57BL/6J	8 wk	4	PR8	10^3 PFU	IN	4
5	C57BL/6J	8 wk	4	PR8	10^3 PFU	IN	6
6	C57BL/6J	8 wk	4	PR8	10^3 PFU	IN	8

[a]PFU, plaque-forming unit.

[b]IN, intranasal inoculation.

Table S15. Primers used for the detection of viral and host genes

Name[a]	Sequence	Size of amplicon (bp)	Reference number
IAV PB1	F: 5’-CTGCCAGAAGACAATGAACC-3’	66	(18)
	R: 5’-GGCCATTGCTTCCAATACAC-3’		
SARS-CoV-2 N	F: 5’-TAATCAGACAAGGAACTGATTA-3’	109	(19)
	R: 5’-CGAAGGTGTGACTTCCATG’ -3’		
RVA VP6	F: 5’-TAGACCAAATAACGTTGAAGTTGA-3’	236	(20)
	R: 5’-GATTCACAAACTGCAGATTCA A-3’		
PEDV N	F: 5’-GCTATGCTCAGATCGCCAGT-3’	92	(21)
	R: 5’-TCTCGTAAGAGTCCGCTAGCTC-3’		
PRRSV M	F: 5’-CACCTCCAGATGCCGTTTG-3’	113	(22)
	R: 5’-ATGCGTGGTTATCATTTGCC-3’		
PSaV VPg	F: 5’-CGAAAGGGAAAAACAAACGC-3’	239	(2)
	R: 5’-TCACTCACTGTCATAGGTGTCACC-3’		
BCoV N	F: 5’-TGGATCAAGATTAGAGTTGGC-3’	236	(23]
	R: 5’-CCTTGTCCATTCTTCTGACC-3’		
Nucleolin	F: 5’-ACCCAGGGGATCACCTAATG-3	153	(24)
	R: 5’-CCTTGGAGGACCCAGTTTC-3		
Human GAPDH	F: 5’-ATTCCACCCATGGCAAATTC-3’	90	(25)
	R: 5’-CGCTCCTGGAAGATGGTGAT-3’		
Mouse GAPDH	F: 5’-AAGGTCATCCCAGAGCTGAA-3’	137	(26)
	R: 5’-CTGCTTCACCACCTTCTTGA-3’		
Porcine GAPDH	F: 5’-ACCTCCACTACATGGTCTACA-3’	90	(27)
	R: 5’-ATGACAAGCTTCCCGTTCTC-3’		
IBV PB1	F: 5’-GGGGGGAGCAGAAGCGGAGC-3’	353	(28)
	R:5’-CCGGGTTATTAGTAGAAACACGAGC-3’		

*Abbreviations: IAV PB1, influenza A virus RNA-directed RNA polymerase catalytic subunit gene; IBV PB1, influenza B virus RNA-directed RNA polymerase catalytic subunit gene; NCL, nucleolin; SARS-CoV-2 N, severe acute respiratory syndrome coronavirus 2 nucleocapsid protein

gene; RVA VP6, species A rotavirus VP6 gene; PEDV N, porcine epidemic diarrhea coronavirus nucleoprotein gene; PRRSV M, porcine reproductive and respiratory syndrome virus matrix protein gene; PSaV VPg, porcine sapovirus viral protein genome-linked; BCoV N, bovine coronavirus nucleocapsid protein gene; GAPDH, glyceraldehyde 3-phosphate dehydrogenase gene.

References

[1] Y. B. Baek *et al*., Therapeutic strategy targeting host lipolysis limits infection by SARS-CoV-2 and influenza A virus. *Signal. Transduct. Target. Ther.* **7**, 367 (2022).

[2] M. M. Alfajaro *et al*., Early porcine sapovirus infection disrupts tight junctions and uses occludin as a coreceptor. *J Virol.* **93,** e01773-18 (2019).

[3] J. van Meerloo, G. J. Kaspers, J. Cloos, Cell sensitivity assays: the MTT assay. *Methods. Mol. Biol.* **731**, 237-45 (2011).

[4] C. M. Clausson *et al*., Increasing the dynamic range of in situ PLA. *Nat. Methods.* **8**, 892–3 (2011).

[5] M. Soliman *et al*., Activation of PI3K, Akt, and ERK during early rotavirus infection leads to V-ATPase-dependent endosomal acidification required for uncoating. *PLoS. Pathog.* **14**, e1006820 (2018).

[6] E. G. Brown, Increased virulence of a mouse-adapted variant of influenza A/FM/1/47 virus is controlled by mutations in genome segments 4, 5, 7, and 8. *J. Virol.* **64**, 4523-33 (1990).

[7] N. A. Ilyushina *et al.,* Adaptation of pandemic H1N1 influenza virus in mice. *J. Virol.* **84**, 8607-16 (2010).

[8] S. Mueller *et al*., Live attenuated influenza virus vaccines by computer-aided rational design. *Nat. Biotechnol.* **28**, 723-6 (2010).

[9] M. A. Ramakrishnan, Determination of 50% endpoint titer using a simple formula. *World. J. Virol.* **5**, 85-6 (2016).

[10] M. D. Tate, A. G. Brooks, P. C. Reading, The role of neutrophils in the upper and lower respiratory tract during influenza virus infection of mice. *Respir. Res.* **9**, 1-13 (2008).

[11] Y. Song, S. L. Li, M. H. Wu, H. J. Li, P. Li, Qualitative and quantitative analysis of iridoid glycosides in the flower buds of Lonicera species by capillary high performance liquid chromatography coupled with mass spectrometric detector. *Anal. Chim. Acta.* **564**, 211-8 (2006).

[12] L. J. Reed, H. Muench, A simple method of estimating fifty percent endpoints. *Am. J. Epidemiol.* **27**, 493-7 (1938).

[13] M. Soliman *et al*., Opposite effects of apoptotic and necroptotic cellular pathways on rotavirus replication. *J. Virol.* **96**, e0122221 (2022).

[14] M. Morita *et al*., The lipid mediator protectin D1 inhibits influenza virus replication and improves severe influenza. *Cell* **153**, 112-25 (2013).

[15] T. D. Schmittgen, K.J. Livak, Analyzing real-time PCR data by the comparative C(T) method. *Nat. Protoc.* **3**, 1101-18 (2008).

[16] S. F. Sia *et al*., Pathogenesis and transmission of SARS-CoV-2 in golden hamsters. *Nature* **583**, 834-8 (2020).

[17] H. Marjuki *et al.,* Influenza A virus-induced early activation of ERK and PI3K mediates V-ATPase-dependent intracellular pH change required for fusion. *Cell. Microbiol.* **13**, 587–601 (2011).

[18] J. Wang *et al.,* Influenza virus exploits an interferon-independent lncRNA to preserve viral RNA synthesis through stabilizing viral RNA polymerase PB1. *Cell. Rep.* **27**, 3295-304.e4 (2019).

[19] N. S. Ogando *et al*., SARS-coronavirus-2 replication in Vero E6 cells: replication kinetics, rapid adaptation and cytopathology. *J. Gen. Virol.* **101**, 925-40 (2020).

[20] B. A. Schwarz, R. Bange, T. W. Vahlenkamp, R. Johne, H. Müller, Detection and quantitation of group A rotaviruses by competitive and real-time reverse transcription-polymerase chain reaction. *J. Virol. Methods.* **105**, 277-85 (2002).

[21] Y. Kim, C. Oh, V. Shivanna, R. A. Hesse, K. O. Chang, Trypsin-independent porcine epidemic diarrhea virus US strain with altered virus entry mechanism. *BMC. Vet. Res.* **13**, 1-13 (2017).

[22] S. Xiao *et al.,* Inhibition of highly pathogenic PRRSV replication in MARC-145 cells by artificial microRNAs. *J. Virol.* **8**, 1-11 (2011).

[23] H. M. Amer, FN Almajhdi, Development of a SYBR Green I based real-time RT-PCR assay for detection and quantification of bovine coronavirus. *Mol. Cell. Probes.* **25,** 101-7 (2011).

[24] Y. Yan, Y. Du, G. Wang, K. Li, Non-structural protein 1 of H3N2 influenza A virus induces

nucleolar stress via interaction with nucleolin. *Sci. Rep.* **7**, 17761 (2017).

[25] F. G. Barr *et al.,* Examination of gene fusion status in archival samples of alveolar rhabdomyosarcoma entered on the Intergroup Rhabdomyosarcoma Study-III trial: a report from the Children's Oncology Group. *J. Mol. Diagn.* **8**, 202-8 (2006).

[26] M. S. Kuefner *et al*., Secretory phospholipase A2 group IIA modulates insulin sensitivity and metabolism. *J. Lipid. Res.* **58**, 1822-33 (2017).

[27] X. Deng *et al*., Development and utilization of an infectious clone for porcine deltacoronavirus strain USA/IL/2014/026. *Virology* **553**, 35-45 (2021).

[28] X. Wang *et al*., Genome characterization of influenza A and B viruses in New South Wales, Australia, in 2019: A retrospective study using high-throughput whole genome sequencing. *Influenza. Other. Respir. Viruses.* **18**, e13252 (2024).

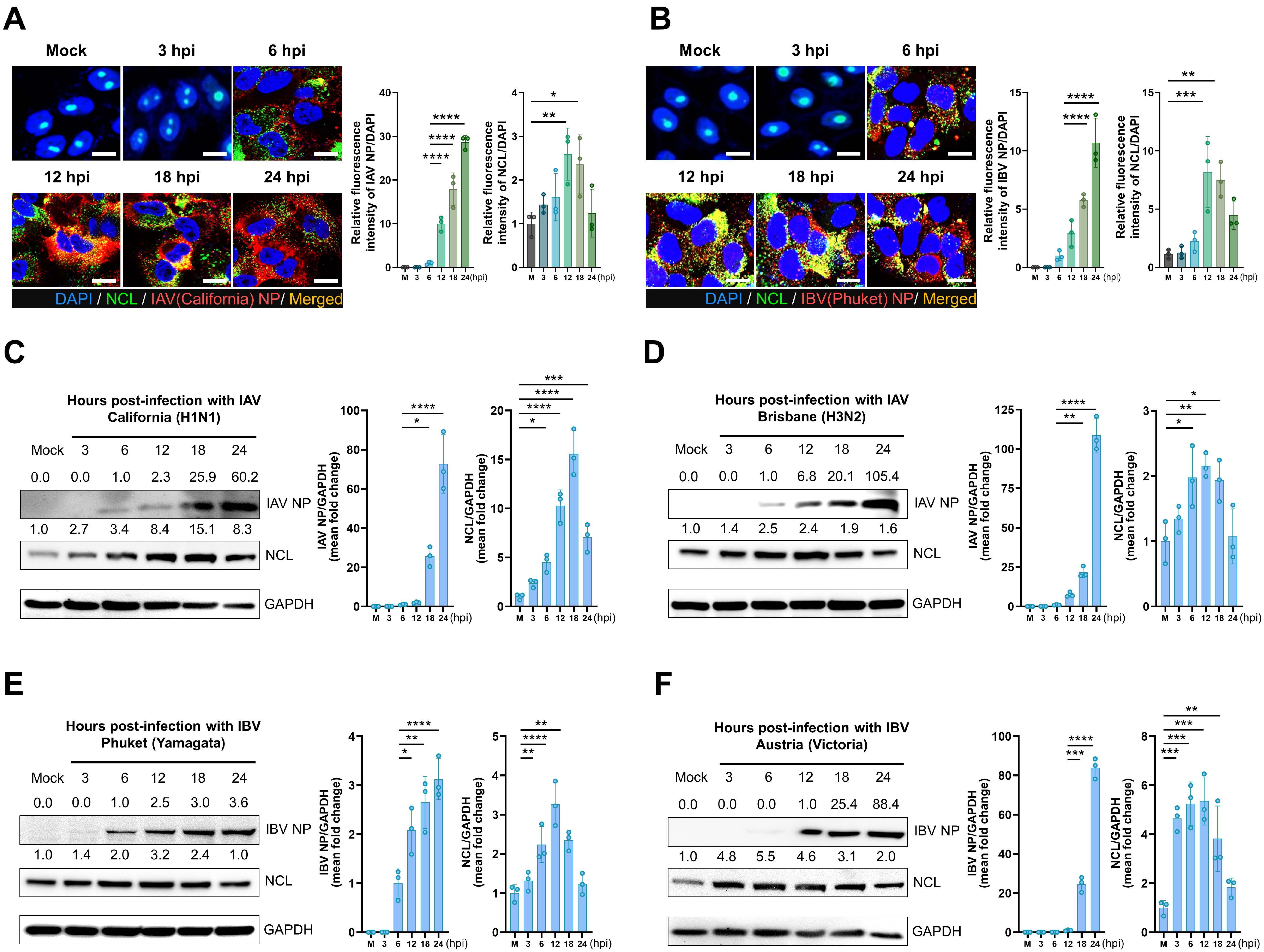
A
Mock
3 hpi
6 hpi
12 hpi
18 hpi
24 hpi
DAPI / NCL / IAV(California) NP/ Merged
Relative fluorescence intensity of IAV NP/DAPI
Relative fluorescence intensity of NCL/DAPI
B
DAPI / NCL / IBV(Phuket) NP/ Merged
Relative fluorescence intensity of IBV NP/DAPI
C
Hours post-infection with IAV California (H1N1)
Mock 3 6 12 18 24
0.0 0.0 1.0 2.3 25.9 60.2
IAV NP
1.0 2.7 3.4 8.4 15.1 8.3
NCL
GAPDH
IAV NP/GAPDH (mean fold change)
NCL/GAPDH (mean fold change)
D
Hours post-infection with IAV Brisbane (H3N2)
Mock 3 6 12 18 24
0.0 0.0 1.0 6.8 20.1 105.4
IAV NP
1.0 1.4 2.5 2.4 1.9 1.6
NCL
GAPDH
E
Hours post-infection with IBV Phuket (Yamagata)
Mock 3 6 12 18 24
0.0 0.0 1.0 2.5 3.0 3.6
IBV NP
1.0 1.4 2.0 3.2 2.4 1.0
NCL
GAPDH
IBV NP/GAPDH (mean fold change)
F
Hours post-infection with IBV Austria (Victoria)
Mock 3 6 12 18 24
0.0 0.0 0.0 1.0 25.4 88.4
IBV NP
1.0 4.8 5.5 4.6 3.1 2.0
NCL
GAPDH
M 3 6 12 18 24 (hpi)

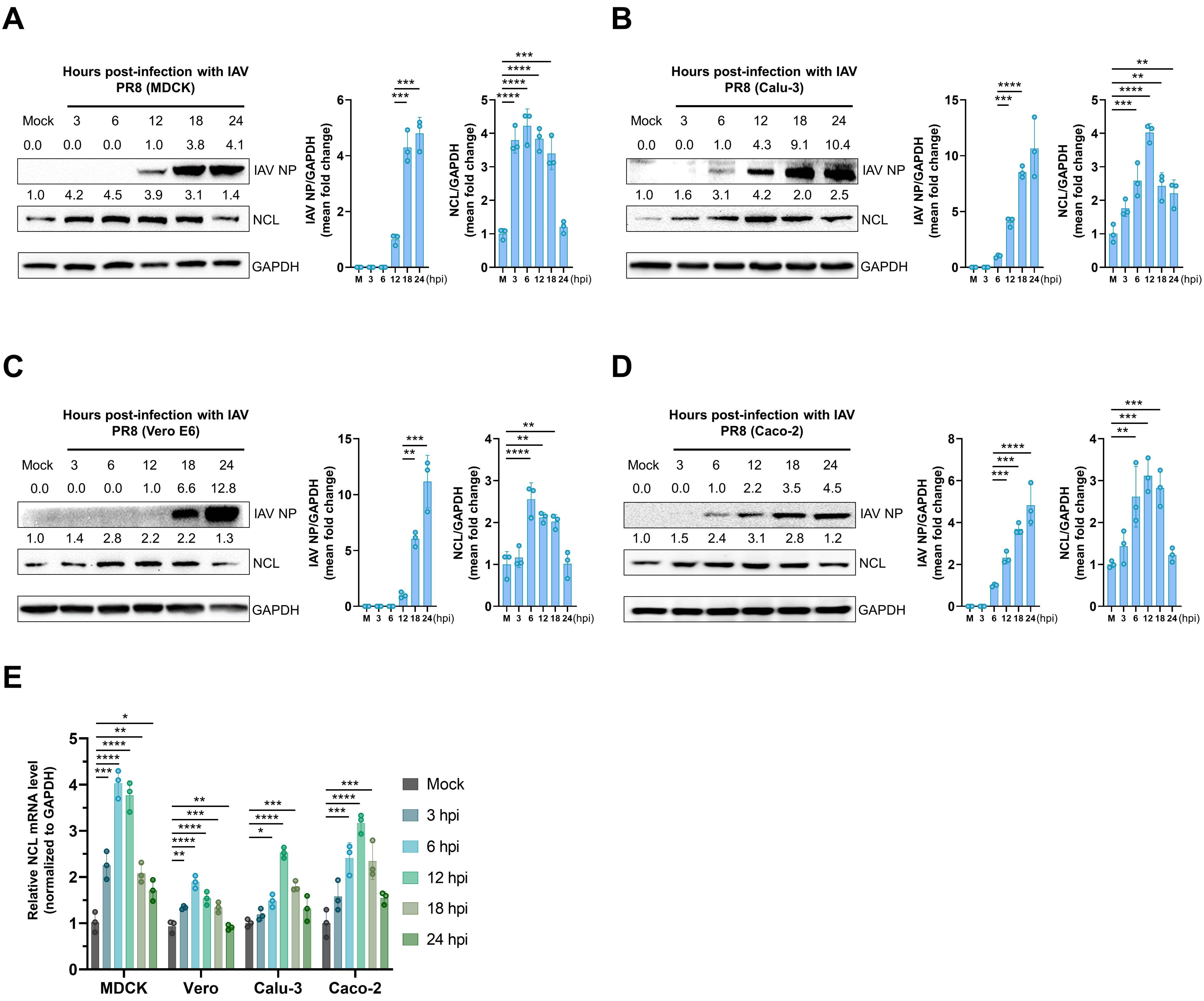
A
Hours post-infection with IAV PR8 (MDCK)
Mock 3 6 12 18 24
0.0 0.0 0.0 1.0 3.8 4.1
IAV NP
1.0 4.2 4.5 3.9 3.1 1.4
NCL
GAPDH
IAV NP/GAPDH (mean fold change)
NCL/GAPDH (mean fold change)
M 3 6 12 18 24 (hpi)
B
Hours post-infection with IAV PR8 (Calu-3)
Mock 3 6 12 18 24
0.0 0.0 1.0 4.3 9.1 10.4
IAV NP
1.0 1.6 3.1 4.2 2.0 2.5
NCL
GAPDH
IAV NP/GAPDH (mean fold change)
NCL/GAPDH (mean fold change)
M 3 6 12 18 24 (hpi)
C
Hours post-infection with IAV PR8 (Vero E6)
Mock 3 6 12 18 24
0.0 0.0 0.0 1.0 6.6 12.8
IAV NP
1.0 1.4 2.8 2.2 2.2 1.3
NCL
GAPDH
IAV NP/GAPDH (mean fold change)
NCL/GAPDH (mean fold change)
M 3 6 12 18 24 (hpi)
D
Hours post-infection with IAV PR8 (Caco-2)
Mock 3 6 12 18 24
0.0 0.0 1.0 2.2 3.5 4.5
IAV NP
1.0 1.5 2.4 3.1 2.8 1.2
NCL
GAPDH
IAV NP/GAPDH (mean fold change)
NCL/GAPDH (mean fold change)
M 3 6 12 18 24 (hpi)
E
Relative NCL mRNA level (normalized to GAPDH)
MDCK
Vero
Calu-3
Caco-2
Mock
3 hpi
6 hpi
12 hpi
18 hpi
24 hpi

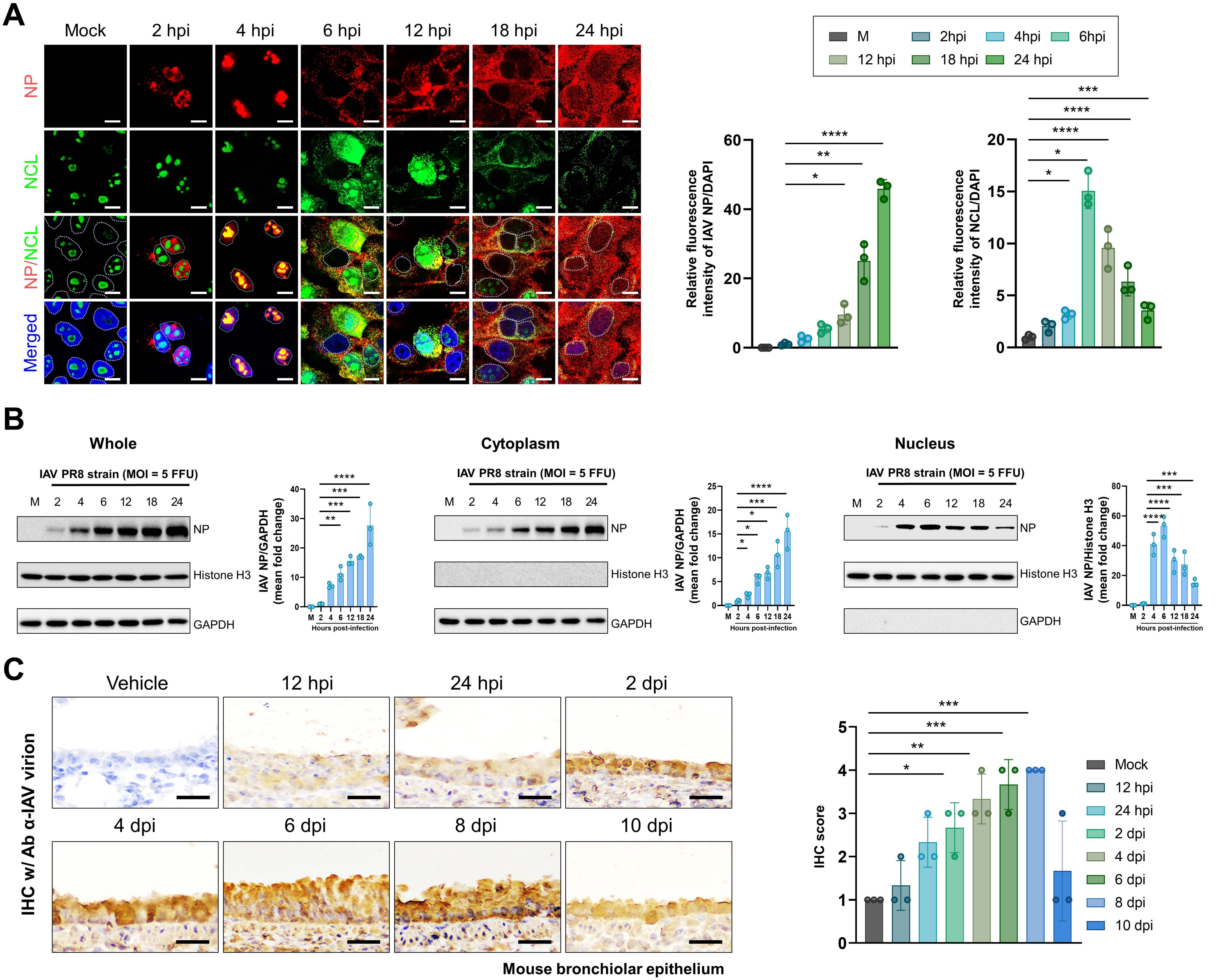

A
Mock
2 hpi
4 hpi
6 hpi
12 hpi
18 hpi
24 hpi
NP
NCL
NP/NCL
Merged
M
2hpi
4hpi
6hpi
12 hpi
18 hpi
24 hpi
Relative fluorescence intensity of IAV NP/DAPI
Relative fluorescence intensity of NCL/DAPI
B
Whole
Cytoplasm
Nucleus
IAV PR8 strain (MOI = 5 FFU)
NP
Histone H3
GAPDH
IAV NP/GAPDH (mean fold change)
IAV NP/Histone H3 (mean fold change)
Hours post-infection
C
Vehicle
12 hpi
24 hpi
2 dpi
4 dpi
6 dpi
8 dpi
10 dpi
IHC w/ Ab α-IAV virion
Mouse bronchiolar epithelium
IHC score
Mock
12 hpi
24 hpi
2 dpi
4 dpi
6 dpi
8 dpi
10 dpi

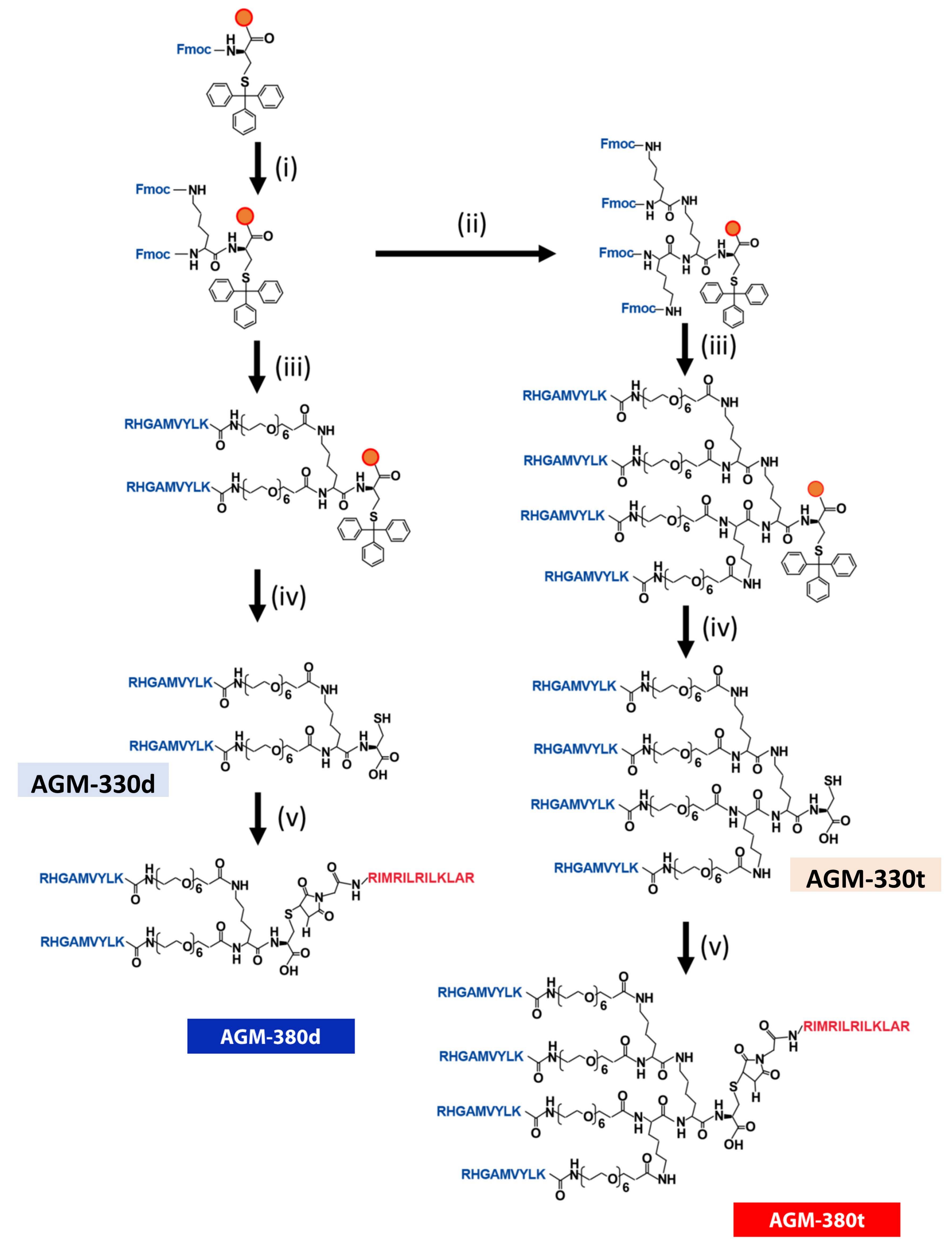

Fmoc
(i)
(ii)
(iii)
(iv)
(v)
RHGAMVYLK
RIMRILRILKLAR
AGM-330d
AGM-380d
AGM-330t
AGM-380t

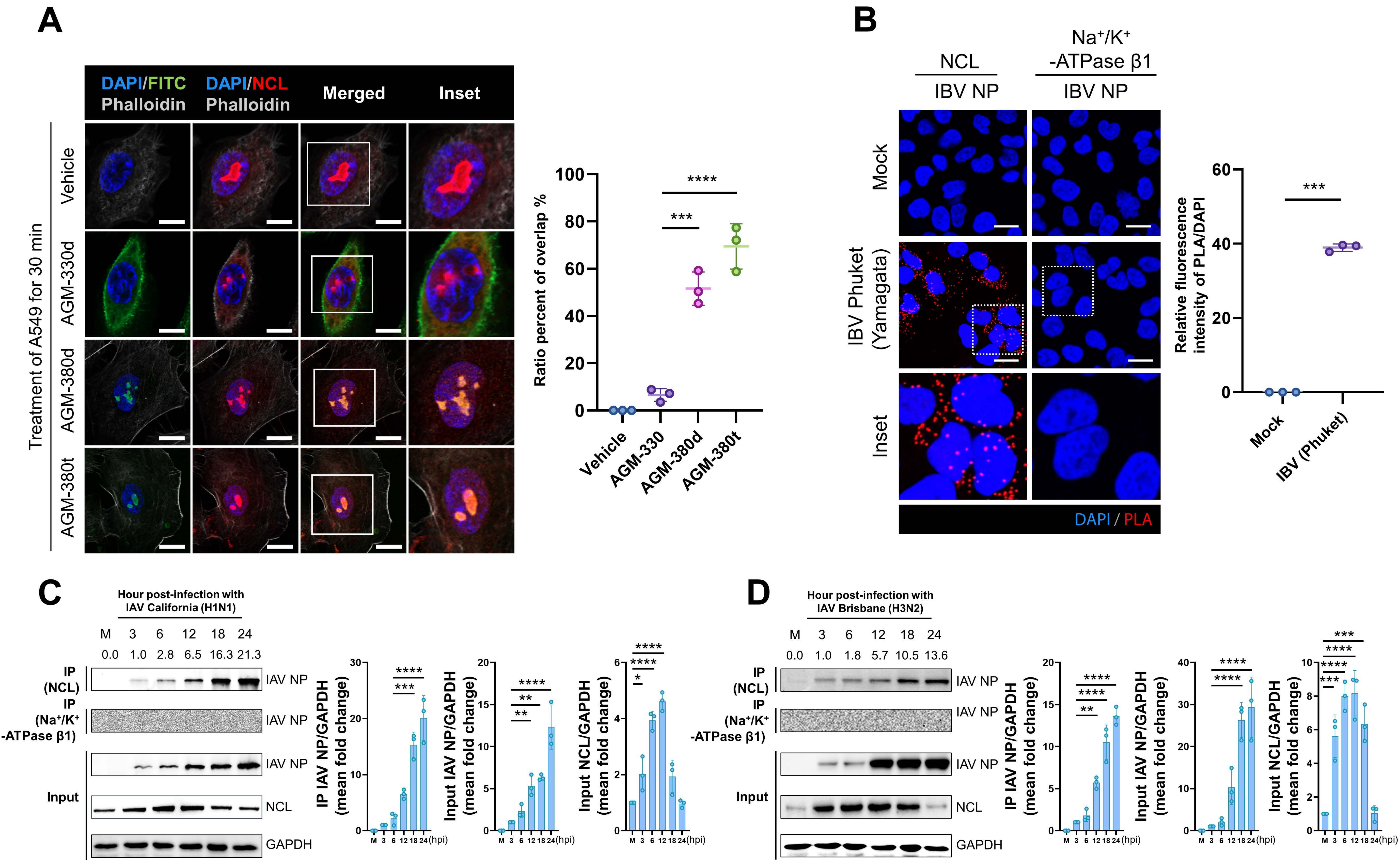
A
DAPI/FITC
Phalloidin
DAPI/NCL
Phalloidin
Merged
Inset
Treatment of A549 for 30 min
Vehicle
AGM-330d
AGM-380d
AGM-380t
Ratio percent of overlap %
Vehicle
AGM-330
AGM-380d
AGM-380t
B
NCL
IBV NP
Na+/K+
-ATPase β1
IBV NP
Mock
IBV Phuket
(Yamagata)
Inset
DAPI / PLA
Relative fluorescence
intensity of PLA/DAPI
Mock
IBV (Phuket)
C
Hour post-infection with
IAV California (H1N1)
M 3 6 12 18 24
0.0 1.0 2.8 6.5 16.3 21.3
IP
(NCL)
IP
(Na+/K+
-ATPase β1)
Input
IAV NP
IAV NP
IAV NP
NCL
GAPDH
IP IAV NP/GAPDH
(mean fold change)
Input IAV NP/GAPDH
(mean fold change)
Input NCL/GAPDH
(mean fold change)
(hpi)
D
Hour post-infection with
IAV Brisbane (H3N2)
M 3 6 12 18 24
0.0 1.0 1.8 5.7 10.5 13.6
IP
(NCL)
IP
(Na+/K+
-ATPase β1)
Input
IAV NP
IAV NP
IAV NP
NCL
GAPDH
IP IAV NP/GAPDH
(mean fold change)
Input IAV NP/GAPDH
(mean fold change)
Input NCL/GAPDH
(mean fold change)
(hpi)

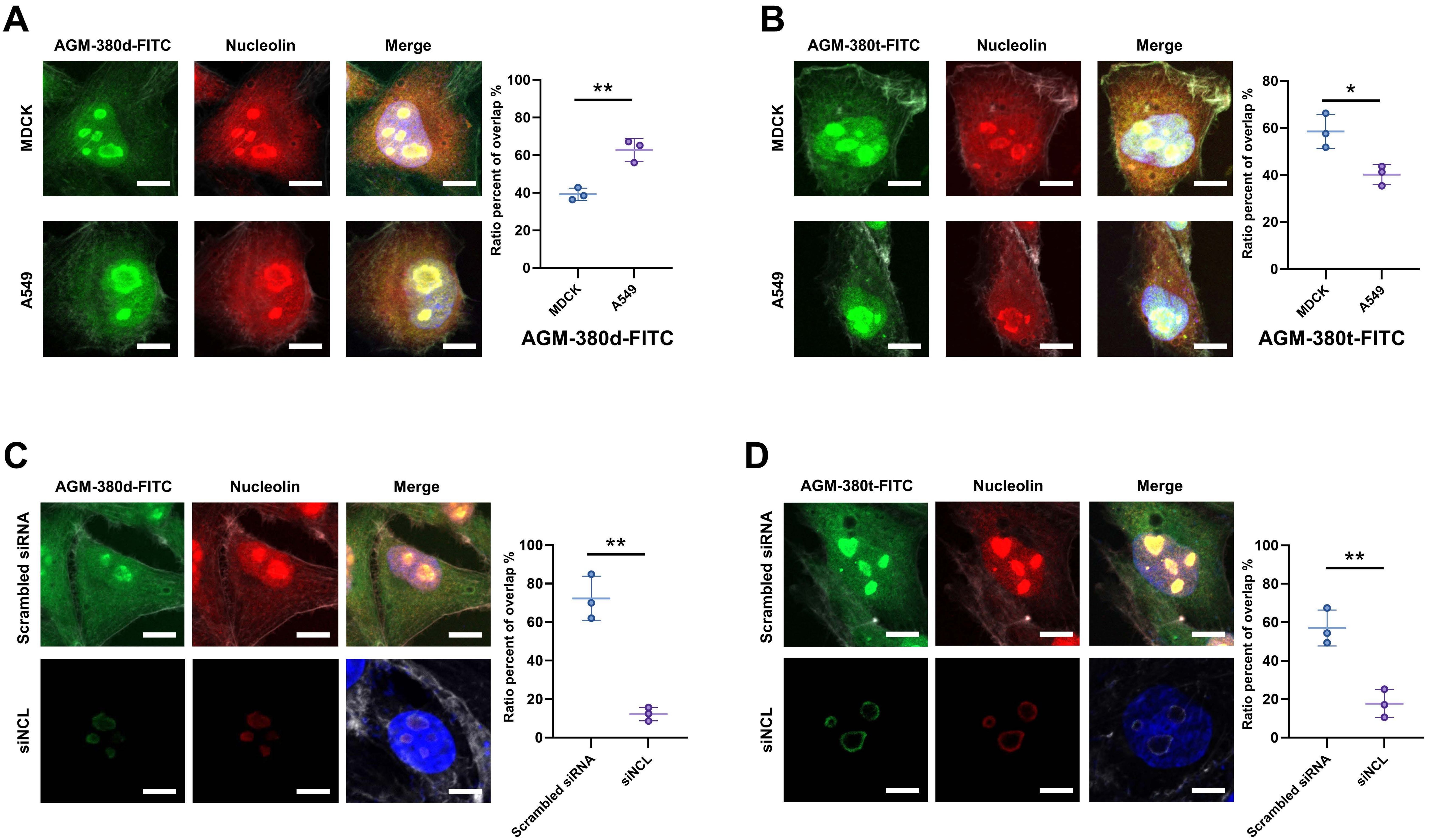

A
AGM-380d-FITC
Nucleolin
Merge
MDCK
A549
Ratio percent of overlap %
100
80
60
40
20
0
**
MDCK
A549
AGM-380d-FITC
B
AGM-380t-FITC
Nucleolin
Merge
MDCK
A549
Ratio percent of overlap %
80
60
40
20
0
*
MDCK
A549
AGM-380t-FITC
C
AGM-380d-FITC
Nucleolin
Merge
Scrambled siRNA
siNCL
Ratio percent of overlap %
100
80
60
40
20
0
**
Scrambled siRNA
siNCL
D
AGM-380t-FITC
Nucleolin
Merge
Scrambled siRNA
siNCL
Ratio percent of overlap %
100
80
60
40
20
0
**
Scrambled siRNA
siNCL

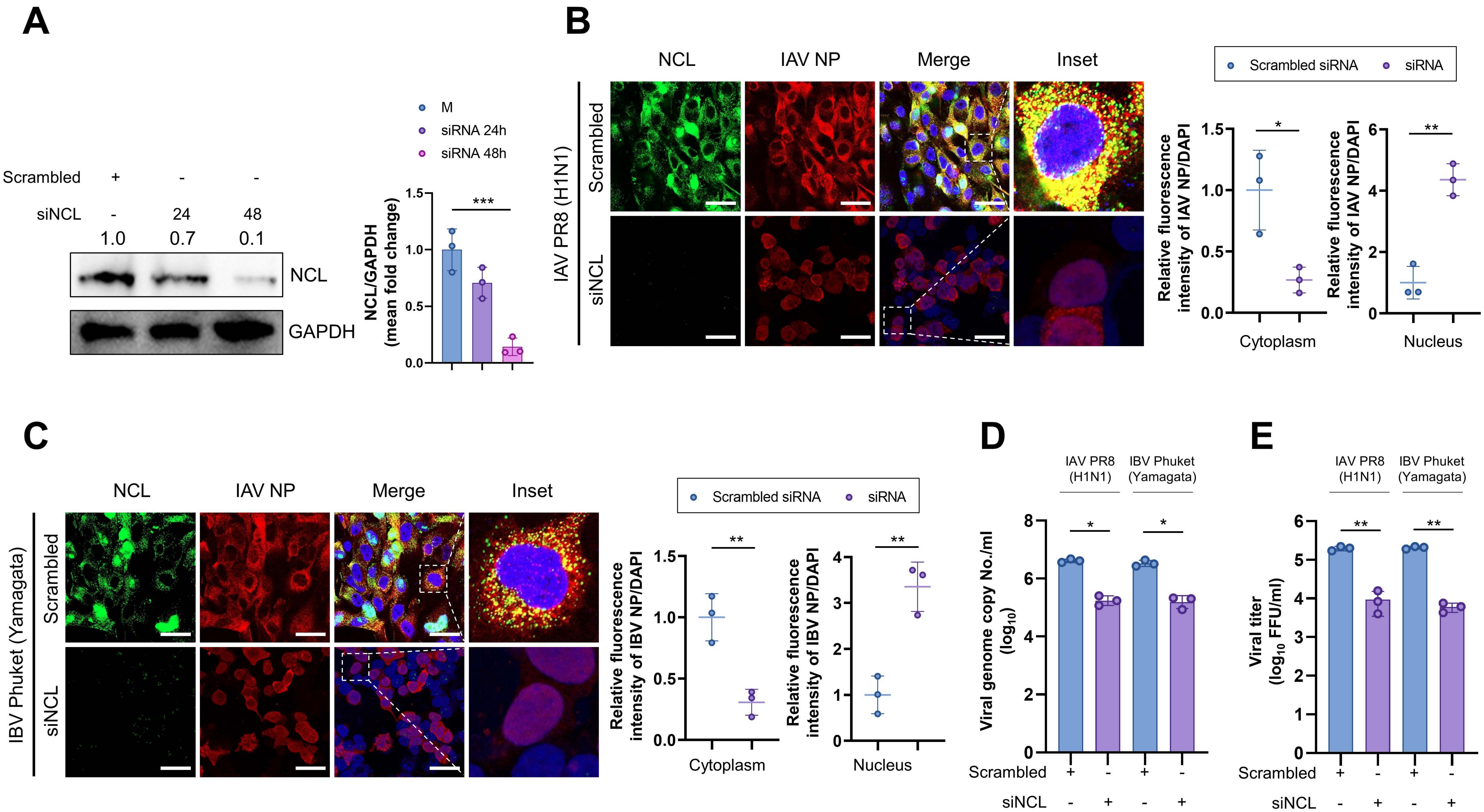
A
Scrambled + - -
siNCL - 24 48
1.0 0.7 0.1
NCL
GAPDH
M
siRNA 24h
siRNA 48h
NCL/GAPDH (mean fold change)
***
B
NCL
IAV NP
Merge
Inset
IAV PR8 (H1N1)
Scrambled
siNCL
Scrambled siRNA
siRNA
Relative fluorescence intensity of IAV NP/DAPI
Cytoplasm
Nucleus
C
NCL
IAV NP
Merge
Inset
IBV Phuket (Yamagata)
Scrambled
siNCL
Scrambled siRNA
siRNA
Relative fluorescence intensity of IBV NP/DAPI
Cytoplasm
Nucleus
D
IAV PR8 (H1N1)
IBV Phuket (Yamagata)
Viral genome copy No./ml (log10)
Scrambled + - + -
siNCL - + - +
E
IAV PR8 (H1N1)
IBV Phuket (Yamagata)
Viral titer (log10 FFU/ml)
Scrambled + - + -
siNCL - + - +

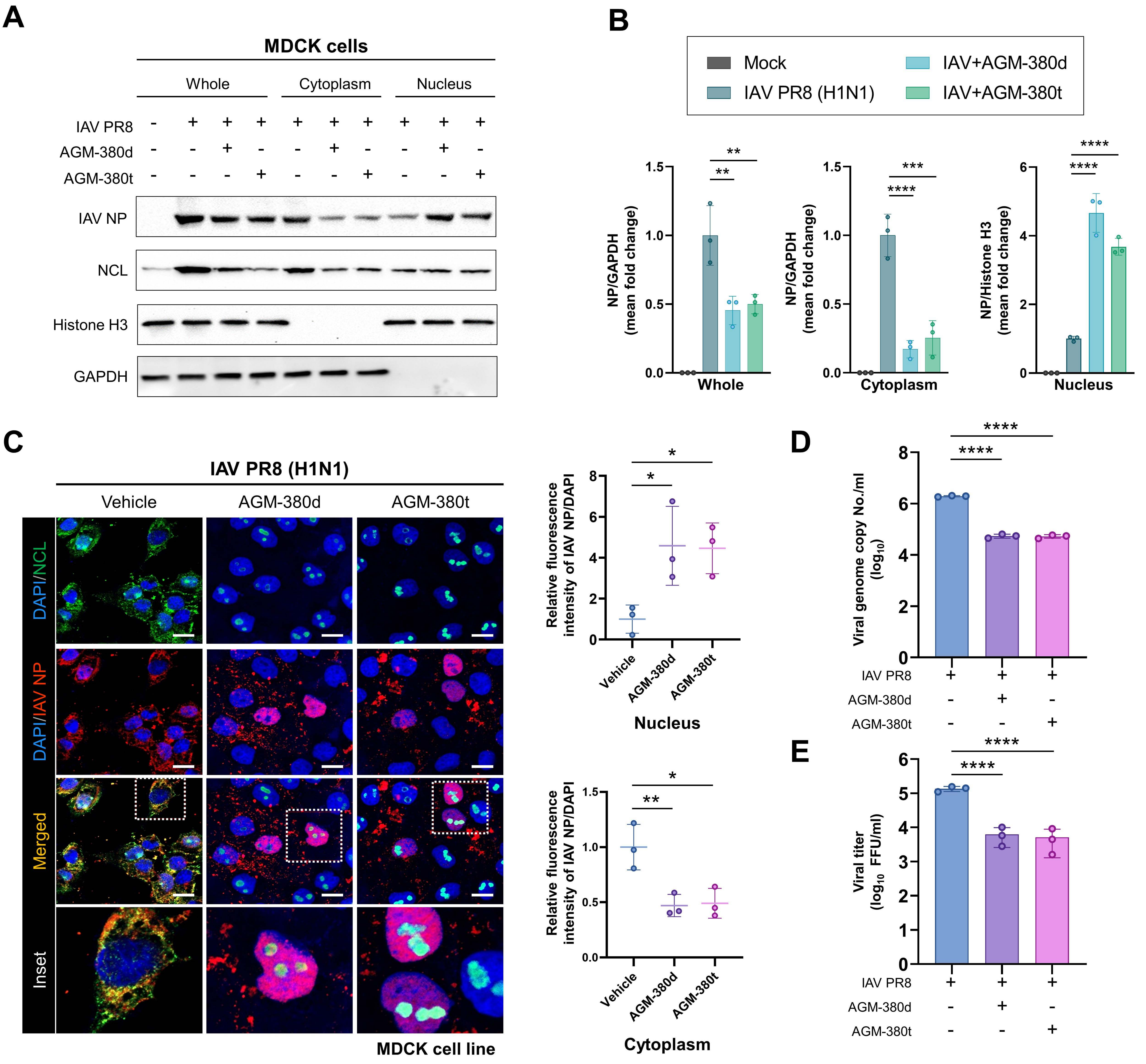
A
MDCK cells
Whole
Cytoplasm
Nucleus
IAV PR8
AGM-380d
AGM-380t
IAV NP
NCL
Histone H3
GAPDH
B
Mock
IAV PR8 (H1N1)
IAV+AGM-380d
IAV+AGM-380t
NP/GAPDH (mean fold change)
NP/Histone H3 (mean fold change)
Whole
Cytoplasm
Nucleus
C
IAV PR8 (H1N1)
Vehicle
AGM-380d
AGM-380t
DAPI/NCL
DAPI/IAV NP
Merged
Inset
MDCK cell line
Relative fluorescence intensity of IAV NP/DAPI
Nucleus
Cytoplasm
D
Viral genome copy No./ml (log10)
E
Viral titer (log10 FFU/ml)

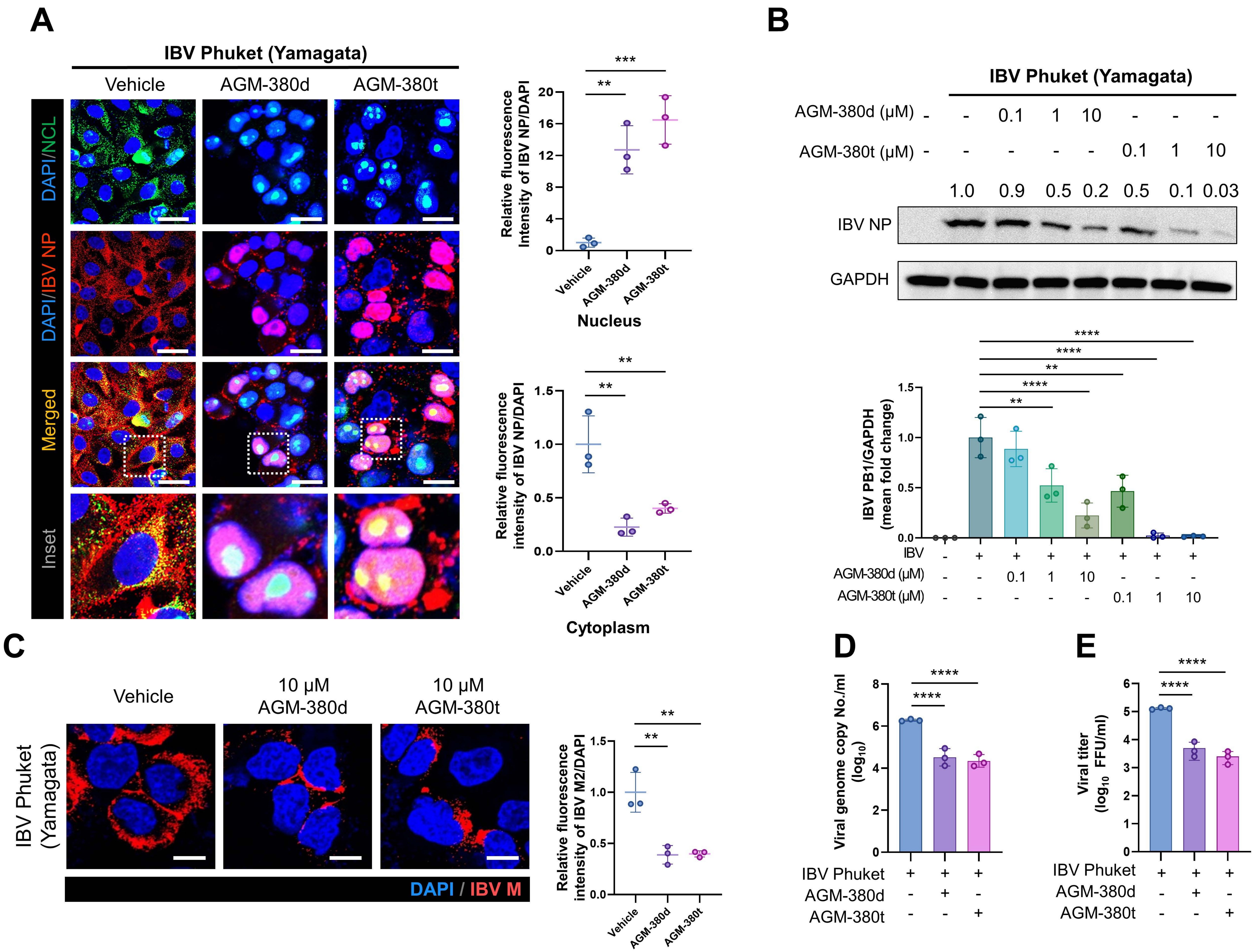

A
IBV Phuket (Yamagata)
Vehicle
AGM-380d
AGM-380t
DAPI/NCL
DAPI/IBV NP
Merged
Inset
Relative fluorescence intensity of IBV NP/DAPI
Nucleus
Cytoplasm
B
IBV Phuket (Yamagata)
AGM-380d (μM)
AGM-380t (μM)
1.0 0.9 0.5 0.2 0.5 0.1 0.03
IBV NP
GAPDH
IBV PB1/GAPDH (mean fold change)
IBV
C
Vehicle
10 μM AGM-380d
10 μM AGM-380t
IBV Phuket (Yamagata)
DAPI / IBV M
Relative fluorescence intensity of IBV M2/DAPI
D
Viral genome copy No./ml (log_{10})
IBV Phuket
E
Viral titer (log_{10} FFU/ml)

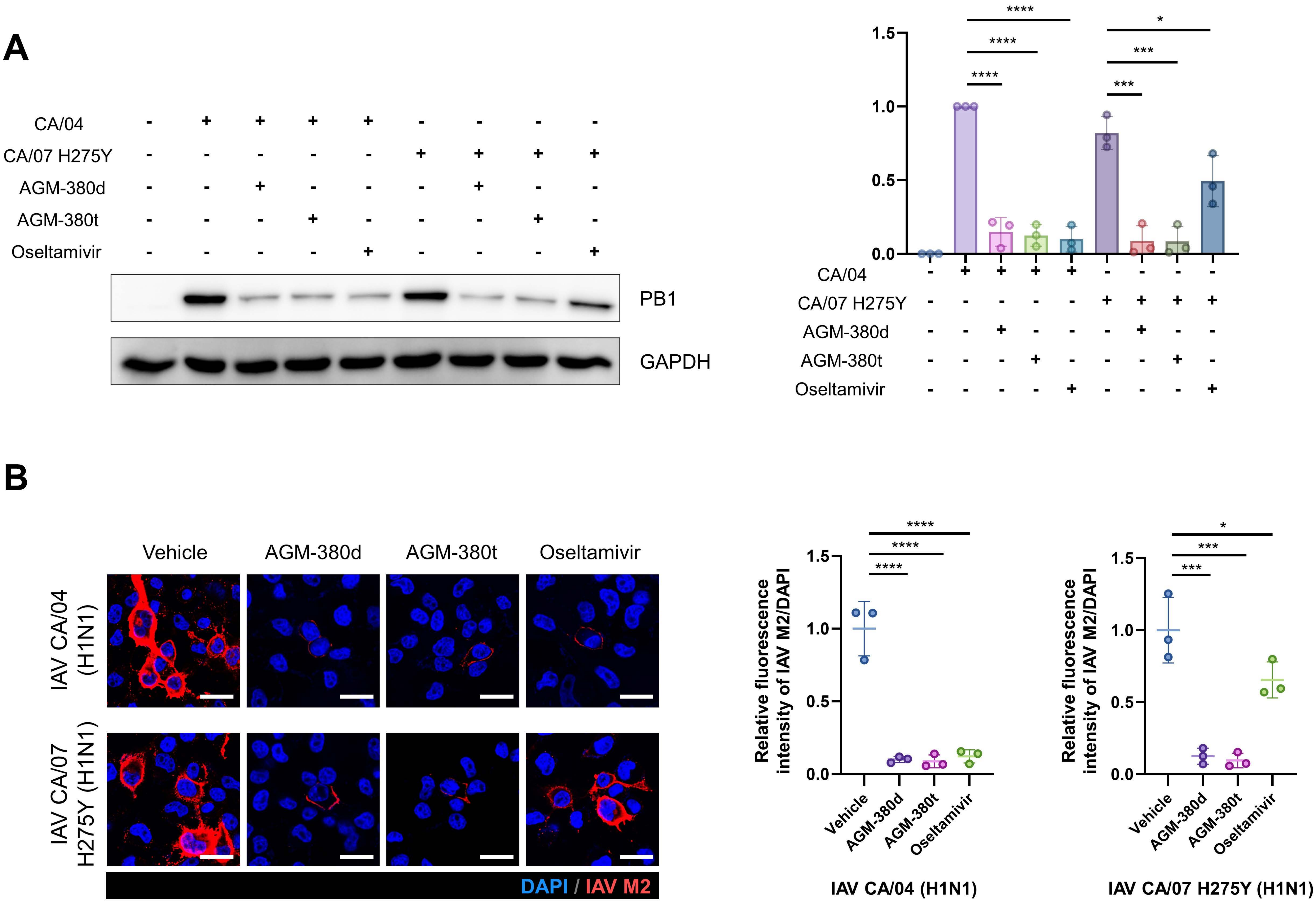
A
CA/04
CA/07 H275Y
AGM-380d
AGM-380t
Oseltamivir
PB1
GAPDH
1.5
1.0
0.5
0.0
****
***
*
B
Vehicle
AGM-380d
AGM-380t
Oseltamivir
IAV CA/04 (H1N1)
IAV CA/07 H275Y (H1N1)
DAPI / IAV M2
Relative fluorescence intensity of IAV M2/DAPI
IAV CA/04 (H1N1)
IAV CA/07 H275Y (H1N1)

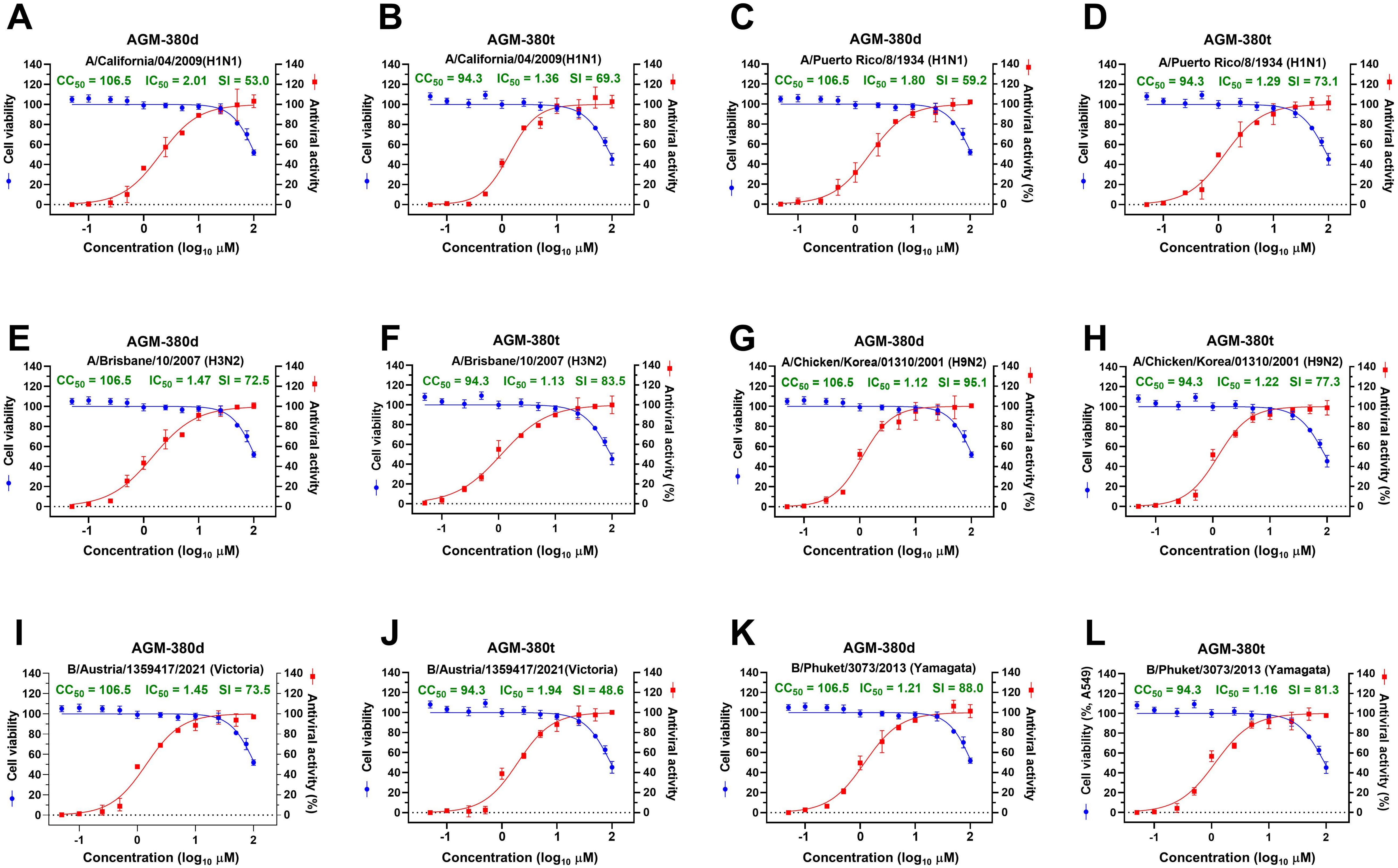
A
AGM-380d
A/California/04/2009(H1N1)
CC50 = 106.5 IC50 = 2.01 SI = 53.0
B
AGM-380t
A/California/04/2009(H1N1)
CC50 = 94.3 IC50 = 1.36 SI = 69.3
C
AGM-380d
A/Puerto Rico/8/1934 (H1N1)
CC50 = 106.5 IC50 = 1.80 SI = 59.2
D
AGM-380t
A/Puerto Rico/8/1934 (H1N1)
CC50 = 94.3 IC50 = 1.29 SI = 73.1
E
AGM-380d
A/Brisbane/10/2007 (H3N2)
CC50 = 106.5 IC50 = 1.47 SI = 72.5
F
AGM-380t
A/Brisbane/10/2007 (H3N2)
CC50 = 94.3 IC50 = 1.13 SI = 83.5
G
AGM-380d
A/Chicken/Korea/01310/2001 (H9N2)
CC50 = 106.5 IC50 = 1.12 SI = 95.1
H
AGM-380t
A/Chicken/Korea/01310/2001 (H9N2)
CC50 = 94.3 IC50 = 1.22 SI = 77.3
I
AGM-380d
B/Austria/1359417/2021 (Victoria)
CC50 = 106.5 IC50 = 1.45 SI = 73.5
J
AGM-380t
B/Austria/1359417/2021(Victoria)
CC50 = 94.3 IC50 = 1.94 SI = 48.6
K
AGM-380d
B/Phuket/3073/2013 (Yamagata)
CC50 = 106.5 IC50 = 1.21 SI = 88.0
L
AGM-380t
B/Phuket/3073/2013 (Yamagata)
CC50 = 94.3 IC50 = 1.16 SI = 81.3
Cell viability
Cell viability (%, A549)
Antiviral activity
Antiviral activity (%)
Concentration (log10 μM)

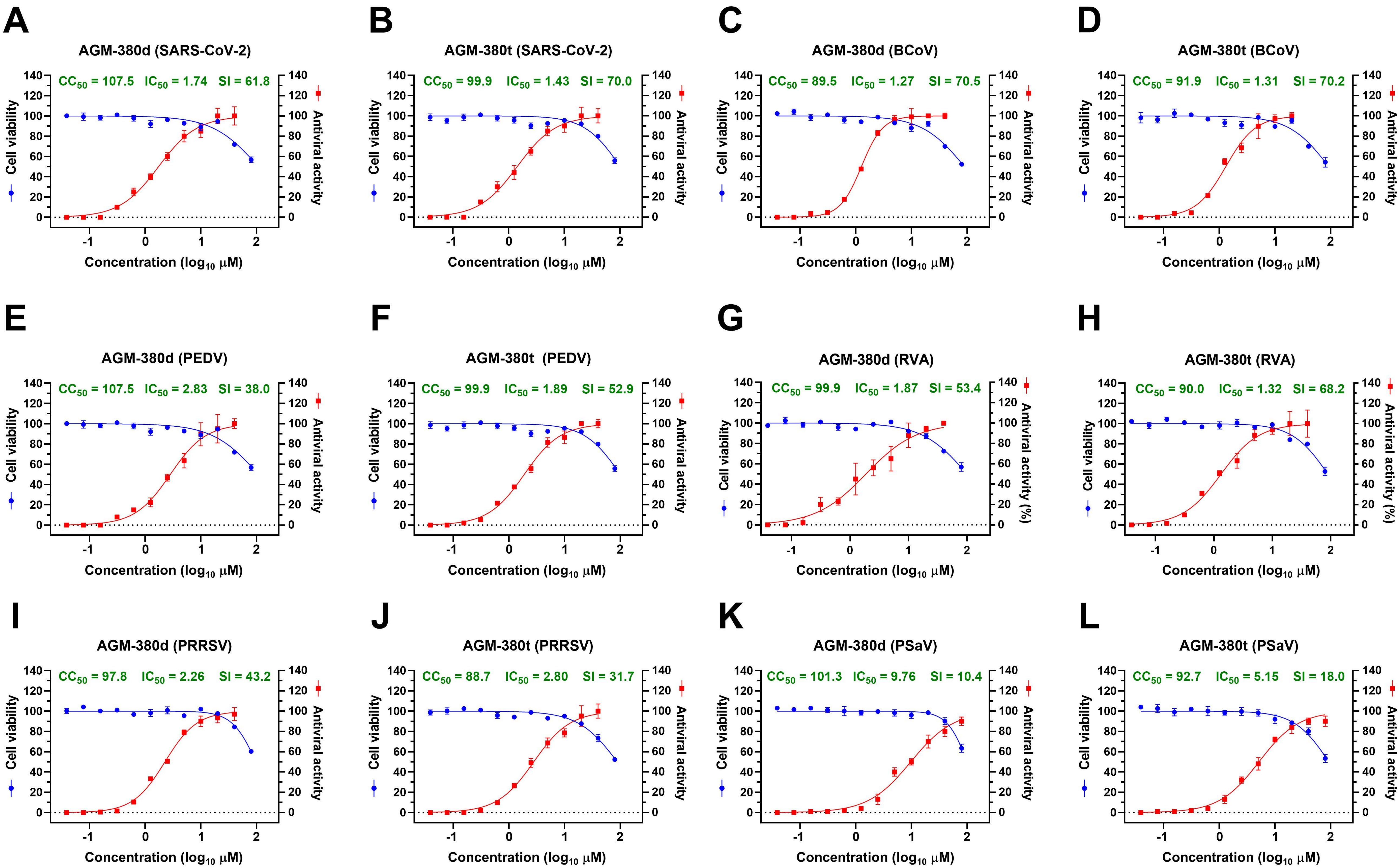
A
AGM-380d (SARS-CoV-2)
CC50 = 107.5 IC50 = 1.74 SI = 61.8
B
AGM-380t (SARS-CoV-2)
CC50 = 99.9 IC50 = 1.43 SI = 70.0
C
AGM-380d (BCoV)
CC50 = 89.5 IC50 = 1.27 SI = 70.5
D
AGM-380t (BCoV)
CC50 = 91.9 IC50 = 1.31 SI = 70.2
E
AGM-380d (PEDV)
CC50 = 107.5 IC50 = 2.83 SI = 38.0
F
AGM-380t (PEDV)
CC50 = 99.9 IC50 = 1.89 SI = 52.9
G
AGM-380d (RVA)
CC50 = 99.9 IC50 = 1.87 SI = 53.4
H
AGM-380t (RVA)
CC50 = 90.0 IC50 = 1.32 SI = 68.2
I
AGM-380d (PRRSV)
CC50 = 97.8 IC50 = 2.26 SI = 43.2
J
AGM-380t (PRRSV)
CC50 = 88.7 IC50 = 2.80 SI = 31.7
K
AGM-380d (PSaV)
CC50 = 101.3 IC50 = 9.76 SI = 10.4
L
AGM-380t (PSaV)
CC50 = 92.7 IC50 = 5.15 SI = 18.0
Cell viability
Antiviral activity
Antiviral activity (%)
Concentration (log10 μM)

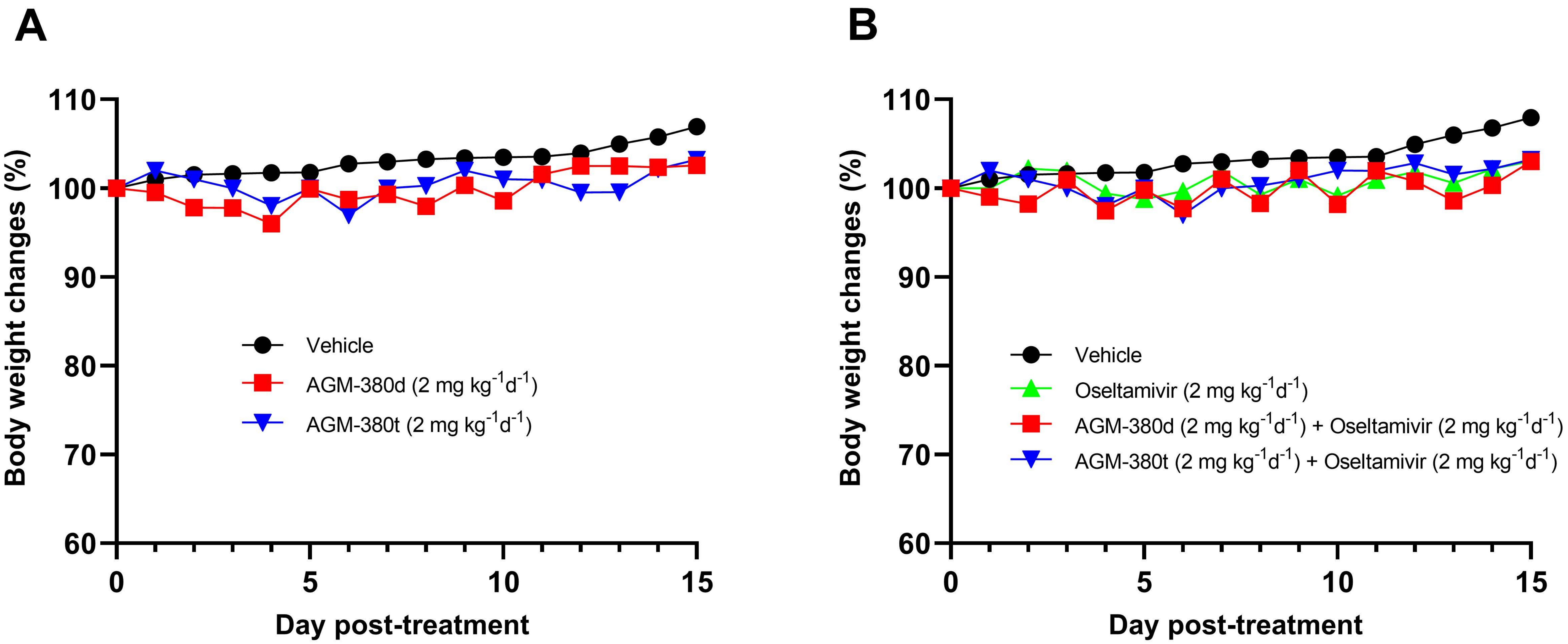

A
B
Body weight changes (%)
110
100
90
80
70
60
0
5
10
15
Day post-treatment
Vehicle
AGM-380d (2 mg kg-1d-1)
AGM-380t (2 mg kg-1d-1)
Oseltamivir (2 mg kg-1d-1)
AGM-380d (2 mg kg-1d-1) + Oseltamivir (2 mg kg-1d-1)
AGM-380t (2 mg kg-1d-1) + Oseltamivir (2 mg kg-1d-1)

Gross findings

Mice

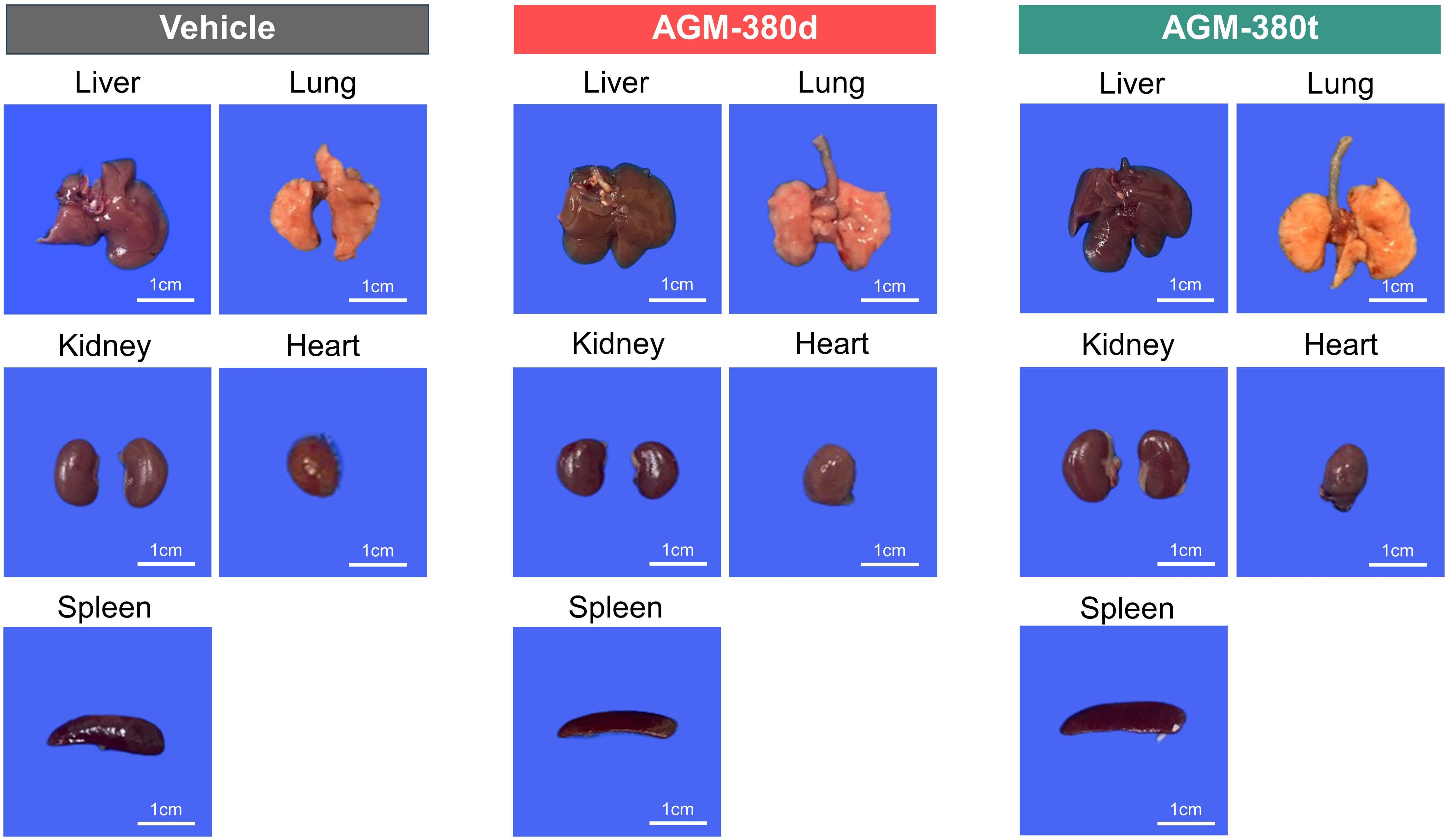

A

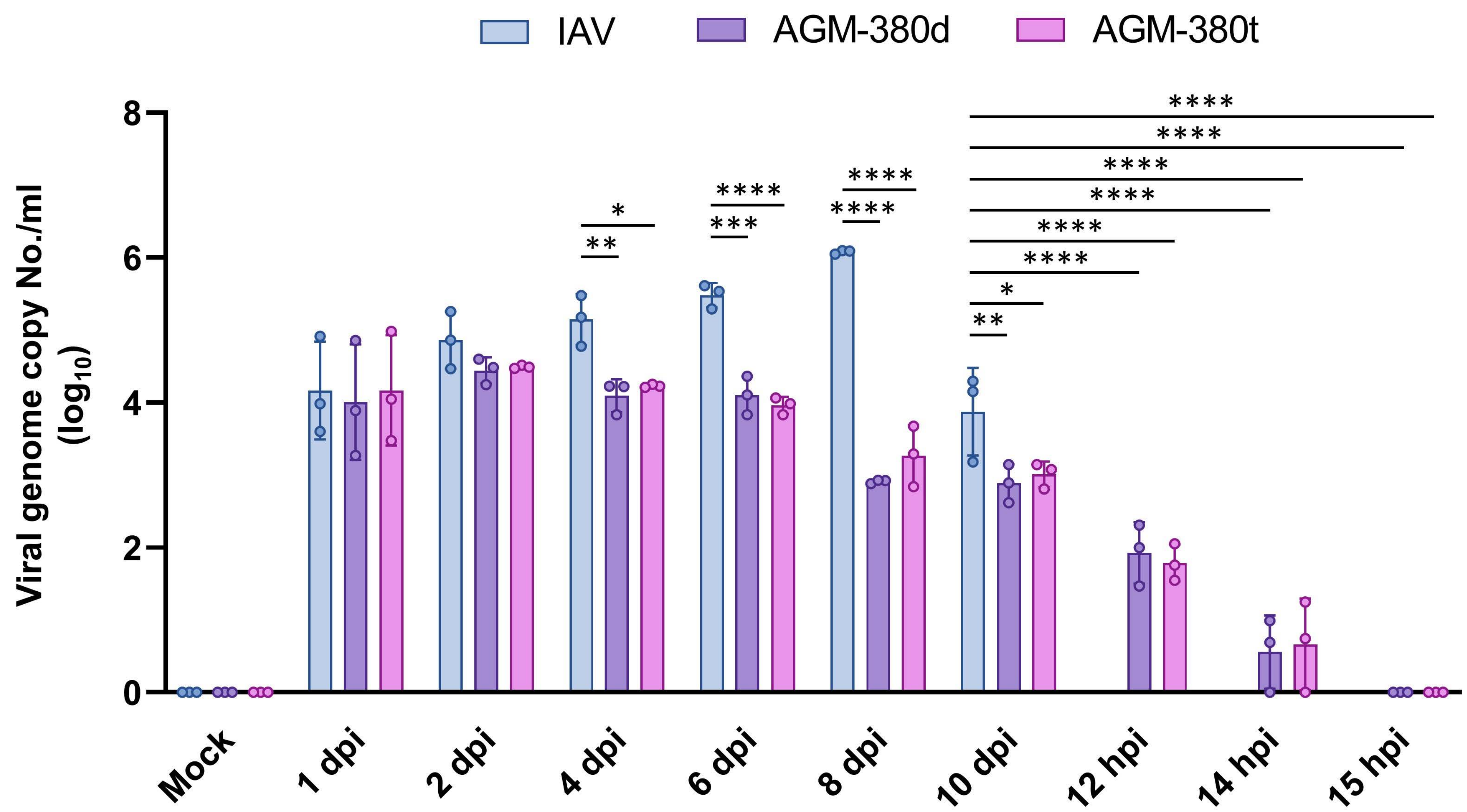
IAV
AGM-380d
AGM-380t
Viral genome copy No./ml (log10)
Mock
1 dpi
2 dpi
4 dpi
6 dpi
8 dpi
10 dpi
12 hpi
14 hpi
15 hpi

B

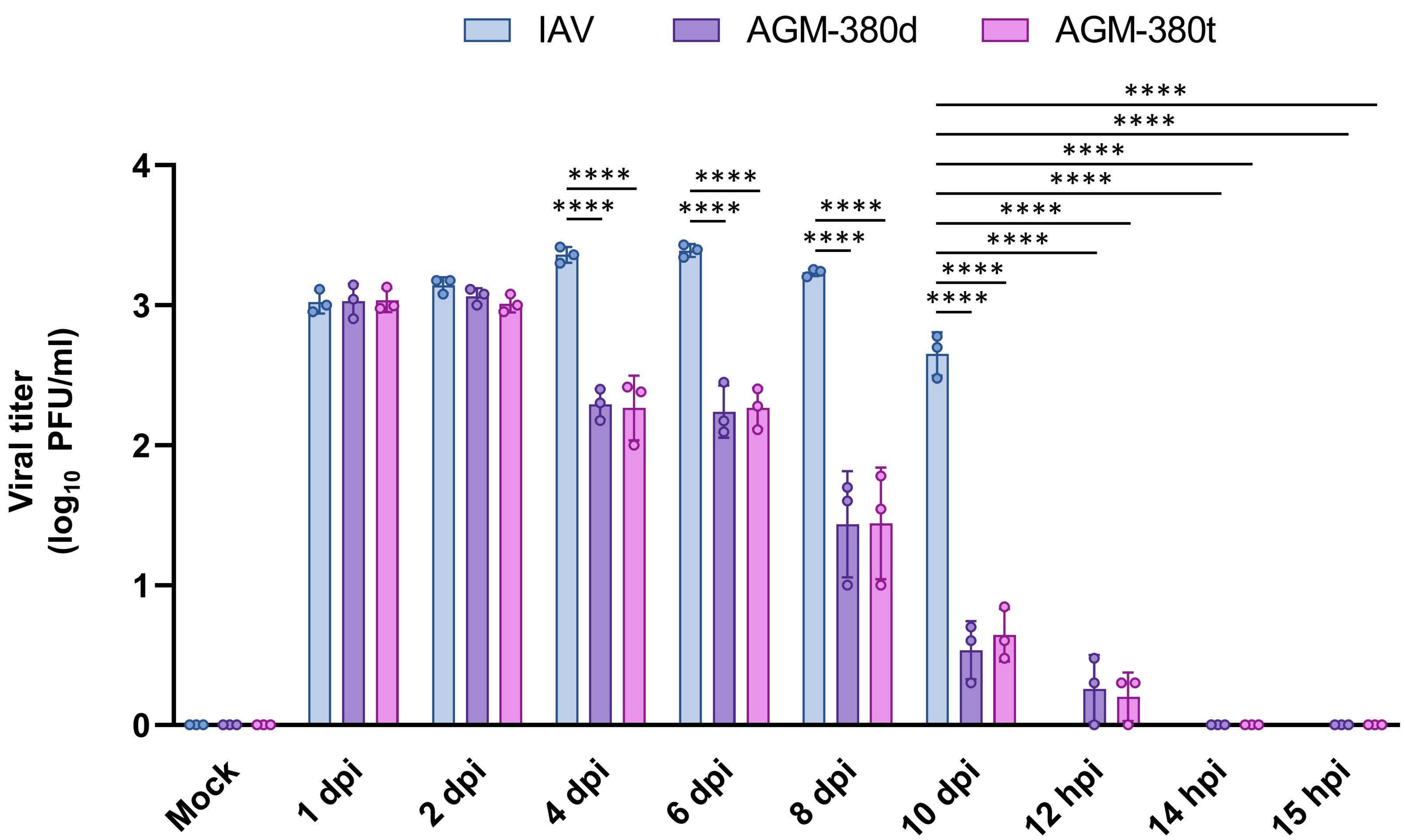
IAV
AGM-380d
AGM-380t
Viral titer (log10 PFU/ml)
Mock
1 dpi
2 dpi
4 dpi
6 dpi
8 dpi
10 dpi
12 hpi
14 hpi
15 hpi

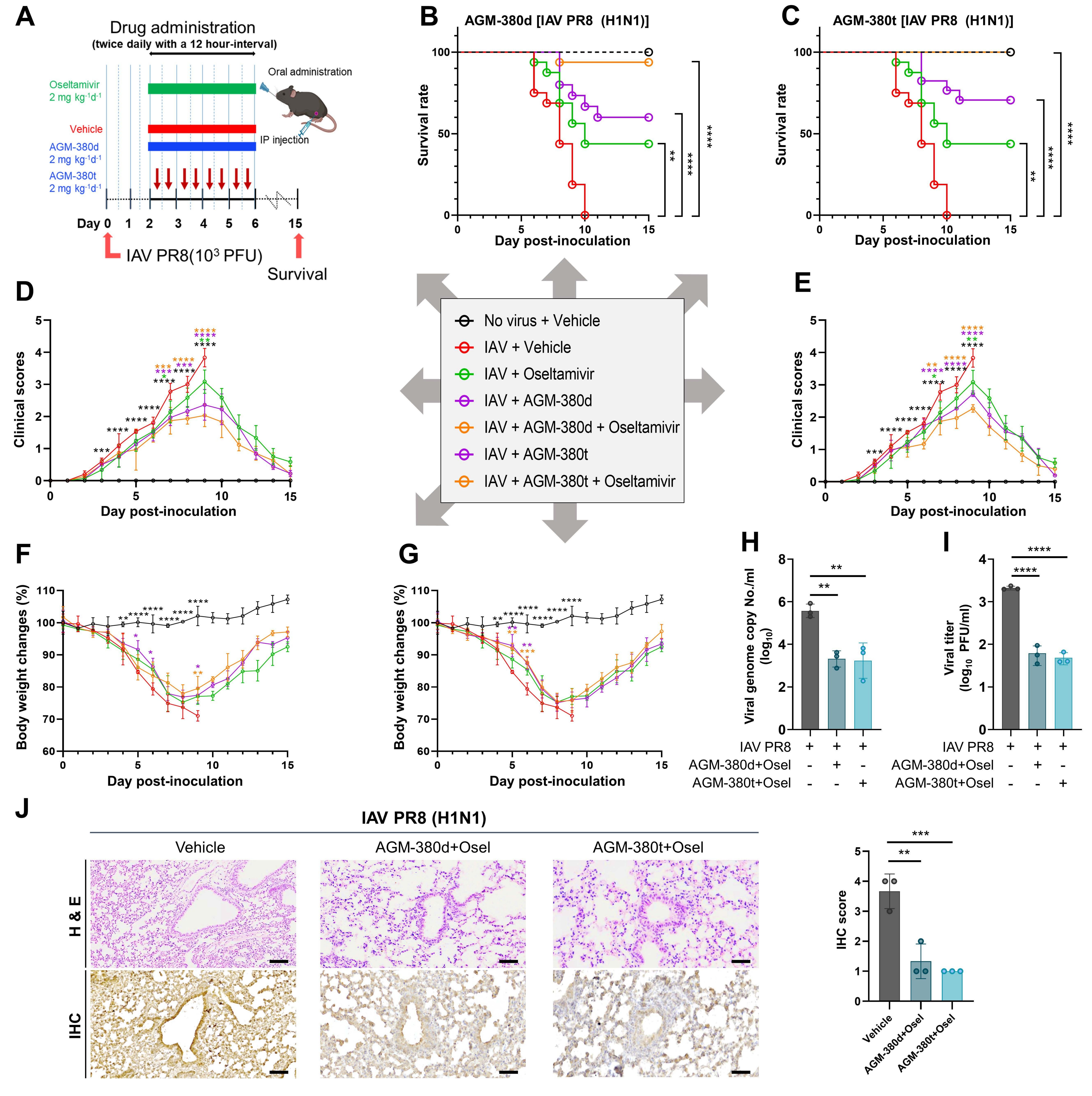

A
Drug administration
(twice daily with a 12 hour-interval)
Oseltamivir 2 mg kg-1d-1
Vehicle
AGM-380d 2 mg kg-1d-1
AGM-380t 2 mg kg-1d-1
Oral administration
IP injection
Day 0 1 2 3 4 5 6 15
IAV PR8(10³ PFU)
Survival
B
AGM-380d [IAV PR8 (H1N1)]
Survival rate
Day post-inoculation
C
AGM-380t [IAV PR8 (H1N1)]
Survival rate
Day post-inoculation
D
Clinical scores
Day post-inoculation
E
Clinical scores
Day post-inoculation
No virus + Vehicle
IAV + Vehicle
IAV + Oseltamivir
IAV + AGM-380d
IAV + AGM-380d + Oseltamivir
IAV + AGM-380t
IAV + AGM-380t + Oseltamivir
F
Body weight changes (%)
Day post-inoculation
G
Body weight changes (%)
Day post-inoculation
H
Viral genome copy No./ml (log10)
IAV PR8
AGM-380d+Osel
AGM-380t+Osel
I
Viral titer (log10 PFU/ml)
IAV PR8
AGM-380d+Osel
AGM-380t+Osel
J
IAV PR8 (H1N1)
Vehicle
AGM-380d+Osel
AGM-380t+Osel
H & E
IHC
IHC score
Vehicle
AGM-380d+Osel
AGM-380t+Osel
